# Goal-directed haemodynamic therapy during general anaesthesia for noncardiac surgery: a systematic review and meta-analysis

**DOI:** 10.1016/j.bja.2021.10.046

**Published:** 2021-12-13

**Authors:** Marie K. Jessen, Mikael F. Vallentin, Mathias J. Holmberg, Maria Bolther, Frederik B. Hansen, Johanne M. Holst, Andreas Magnussen, Niklas S. Hansen, Cecilie M. Johannsen, Johannes Enevoldsen, Thomas H. Jensen, Lara L. Roessler, Peter C. Lind, Maibritt P. Klitholm, Mark A. Eggertsen, Philip Caap, Caroline Boye, Karol M. Dabrowski, Lasse Vormfenne, Maria Høybye, Jeppe Henriksen, Carl M. Karlsson, Ida R. Balleby, Marie S. Rasmussen, Kim Pælestik, Asger Granfeldt, Lars W. Andersen

**Affiliations:** 1 Research Center for Emergency Medicine, Aarhus University and Aarhus University Hospital, Aarhus, Denmark; 2 Department of Clinical Medicine, Aarhus University, Aarhus, Denmark; 3 Prehospital Emergency Medical Services, Central Denmark Region, Aarhus, Denmark; 4 Department of Cardiology, Viborg Regional Hospital, Viborg, Denmark; 5 Department of Anesthesiology and Intensive Care, Aarhus University Hospital, Aarhus, Denmark; 6 Department of Internal Medicine, University Hospital of North Norway, Narvik, Norway; 7 Department of Emergency Medicine, Department of Clinical Research, University of Southern Denmark, Odense, Denmark; 8 Department of Surgical Gastroenterology, Aalborg University Hospital, Aalborg, Denmark; 9 Department of Anesthesiology and Intensive Care, Aalborg University Hospital, Aalborg, Denmark; 10 National Hospital of the Faroe Islands, Torshavn, Faroe Islands, Denmark; 11 Department of Anesthesiology and Intensive Care, Viborg Regional Hospital, Viborg, Denmark

**Keywords:** fluid, general anaesthesia, goal-directed haemodynamic therapy, haemodynamics, perioperative care, postoperative complications, stroke volume

## Abstract

**Background:**

During general anaesthesia for noncardiac surgery, there remain knowledge gaps regarding the effect of goal-directed haemodynamic therapy on patient-centred outcomes.

**Methods:**

Included clinical trials investigated goal-directed haemodynamic therapy during general anaesthesia in adults undergoing noncardiac surgery and reported at least one patient-centred postoperative outcome. PubMed and Embase were searched for relevant articles on March 8, 2021. Two investigators performed abstract screening, full-text review, data extraction, and bias assessment. The primary outcomes were mortality and hospital length of stay, whereas 15 postoperative complications were included based on availability. From a main pool of comparable trials, meta-analyses were performed on trials with homogenous outcome definitions. Certainty of evidence was evaluated using Grading of Recommendations, Assessment, Development, and Evaluations (GRADE).

**Results:**

The main pool consisted of 76 trials with intermediate risk of bias for most outcomes. Overall, goal-directed haemodynamic therapy might reduce mortality (odds ratio=0.84; 95% confidence interval [CI], 0.64 to 1.09) and shorten length of stay (mean difference=–0.72 days; 95% CI, –1.10 to –0.35) but with low certainty in the evidence. For both outcomes, larger effects favouring goal-directed haemodynamic therapy were seen in abdominal surgery, very high-risk surgery, and using targets based on preload variation by the respiratory cycle. However, formal tests for subgroup differences were not statistically significant. Goal-directed haemodynamic therapy decreased risk of several postoperative outcomes, but only infectious outcomes and anastomotic leakage reached moderate certainty of evidence.

**Conclusions:**

Goal-directed haemodynamic therapy during general anaesthesia might decrease mortality, hospital length of stay, and several postoperative complications. Only infectious postoperative complications and anastomotic leakage reached moderate certainty in the evidence.

**Editor's key points**

• Previous systematic reviews have shown that perioperative, goal-directed, haemodynamic therapy might reduce postoperative complications. It is not clear how patient and procedure heterogeneity or recent publications affect these findings.
• This comprehensive systematic review found that goal-directed, haemodynamic therapy during general anaesthesia for noncardiac surgery reduced postoperative pneumonia, surgical site infection, and anastomotic leakage (with moderate certainty in the evidence). The effects on mortality and hospital length of stay were unclear.
• Large clinical trials are needed to examine the effect on mortality and hospital length of stay. At least three such trials are currently ongoing.

Worldwide, more than 300 million major surgeries are conducted each year^1^ with the vast majority of these requiring general anaesthesia. Although general anaesthesia is generally considered safe, certain patients are at a higher risk of intra- and postoperative complications and mortality. Common postoperative complications include infection, bleeding, cardiac complications, pulmonary complications, acute kidney injury, and delirium.^2^, ^3^, ^4^, ^5^

To minimise these risks, clinicians provide intraoperative interventions with the aim of obtaining specific physiological, respiratory, and haemodynamic targets. Yet, little is known about which targets are optimal for which patients in which types of surgery; this limits the possibility of clear guidelines and there are differences in treatment protocols from hospital to hospital. Hence, there is a strong need for evidence-based intraoperative targets.

Goal-directed haemodynamic therapy (GDHT) – sometimes just called goal-directed therapy^6^ – is the use of a protocol to standardise haemodynamic targets and the treatments used to reach these targets. GDHT most often refers to optimisation of flow-related parameters such as cardiac output or stroke volume,^7^^,^^8^ and optimisation will most often involve fluid therapy and the term goal-directed fluid therapy is therefore also used.^9^, ^10^, ^11^ Systematic reviews have generally found that GDHT reduces hospital length of stay^9^^,^^12^, ^13^, ^14^, ^15^, ^16^, ^17^ and overall postoperative complication rate,^7^^,^^9^^,^^14^, ^15^, ^16^^,^^18^, ^19^, ^20^ whereas estimates on mortality^7^^,^^12^^,^^13^^,^^16^^,^^18^^,^^19^^,^^21^, ^22^, ^23^ and organ-specific complications^9^^,^^13^^,^^14^^,^^16^^,^^20^^,^^22^, ^23^, ^24^, ^25^, ^26^ tend to favour GDHT with varying precision. Yet, there may be problems with heterogeneity in outcome definitions: although one review did select studies for meta-analyses based on specific definitions of pulmonary outcomes,^17^ other reviews included all outcome definitions. On the contrary, a 2018 review found all included GDHT trials too heterogeneous to perform any meta-analysis on any outcome.^6^

This comprehensive systematic review aims to describe the literature on intraoperative GDHT. When deemed appropriate, meta-analyses will be performed on a wide range of patient-centred outcomes while exploring potential heterogeneity. The goal is to provide an overview for clinicians involved in patient care and for researchers to guide future work.

## Methods

### Protocol and registration

The protocol for the current review is provided in Supplementary Content S1. The protocol was prospectively uploaded to Figshare (figshare.com) on June 11, 2020 and updated on August 19, 2020. The reporting of this systematic review followed the Preferred Reporting Items for Systematic Reviews and Meta-Analyses (PRISMA) guidelines.^27^ The PRISMA checklist is provided in Supplementary Content S2, section ‘PRISMA-checklist’.

### Eligibility criteria and outcomes

This review was part of a larger review project including trials of adult patients undergoing noncardiac surgery with general anaesthesia and mechanical ventilation. The project investigates whether the use of specific intraoperative physiologic targets improves patient-centred postoperative outcomes. Trials involving very short durations of anaesthesia (e.g. for electroconvulsive therapy), Caesarean sections, or procedures with one-lung ventilation were not included. All years, but only English language publications, were included.

This particular article focuses on trials of GDHT during general anaesthesia, that is trials investigating treatment protocols designed to reach one or more specific haemodynamic targets. There were no limits on the type of haemodynamic variable, nor the type of device used to measure it. Accepted comparators were other treatment protocols or standard care. Trials comparing different fluid strategies without specific targets were not included. Trials solely focusing on blood pressure targets are traditionally not considered GDHT and were not included in this review.

Included trials had to report at least one patient-centred postoperative outcome, meaning clinical outcomes considered directly related to patient morbidity or mortality. We considered mortality and hospital length of stay as the primary outcomes. Secondary outcomes were pneumonia, pulmonary oedema, acute respiratory distress syndrome, pulmonary embolism, myocardial infarction, arrhythmia, acute kidney injury, surgical site infection, ileus, anastomotic leakage, and delirium. These postoperative outcomes were prioritised based on the available outcomes reported in the included trials. We also extracted data on combined pulmonary complications, acute lung injury, combined cardiac complications, and combined abdominal complications, but these outcomes were not considered further primarily because of incomparable outcome definitions. There were no limits on an individual trial's definitions of the outcomes, but definitions were noted for each outcome in each trial to enable assessment of heterogeneity.

### Information sources and search strategy

On July 24, 2020, and again on March 8, 2021, we searched PubMed and Embase. The search included a combination of various text and indexing search terms for general anaesthesia or surgery and various haemodynamic and respiratory targets. To identify randomised trials, the Cochrane sensitivity-maximising search strategy was used.^28^ The search strategy for each database is provided in the protocol. The reference lists of included articles and recent systematic reviews were reviewed for potential additional articles.

To identify registered ongoing or unpublished trials, we searched the International Clinical Trials Registry Platform and ClinicalTrials.gov on April 5, 2021, and again on June 28, 2021. Additional details are provided in Supplementary Content S2, section ‘Ongoing randomized clinical trials’.

### Study selection

Two reviewers independently screened all titles and abstracts retrieved from the systematic searches. The kappa values for inter-observer variance were calculated. Relevant titles and abstracts were independently assessed in full text by two reviewers. For ongoing randomised clinical trials, two reviewers independently screened titles and trial registrations for relevant articles.

In all steps, any disagreement regarding eligibility was resolved via discussion between the reviewers and a third investigator as needed.

### Data collection

Two reviewers extracted data from individual articles using a predefined standardised data extraction form. Any discrepancies in the extracted data were identified and resolved via discussion. All GDHT protocol targets and interventions were only noted if they were different from the comparator, for example if both groups used vasopressors to reach a mean arterial pressure of ≥65 mm Hg, then mean arterial pressure was not considered a GDHT target and vasopressors were not considered a GDHT intervention.

### Risk of bias in individual studies

Two reviewers independently assessed risk of bias for individual trials using version 2 of the Cochrane risk-of-bias tool for randomised trials.^29^ Disagreements were resolved via discussion. Risk of bias was assessed for each outcome within a trial but is reported at the trial level as the highest risk of bias across all outcomes. If the bias was different for different outcomes, this was noted. Additional considerations about bias assessment are provided in Supplementary Content S2, section ‘Risk of bias assessment’.

### Data synthesis

Trials were evaluated for clinical heterogeneity (i.e. population, intervention, comparator, and outcome) and methodological heterogeneity to determine whether they could be combined in meta-analyses. Additional details are provided in Supplementary Content S2, sections ‘Pooling of trials based on heterogeneity’ and ‘Outcomes: Definitions, data synthesis, and sensitivity analyses’.

To ensure comparability of the intervention and comparator, we made the following choices regarding articles considered for meta-analyses: First, we excluded GDHT protocols without a fluid therapy intervention as almost all trials had one or more targets related to fluid therapy. Second, the haemodynamic target determining fluid therapy was limited to those either directly or indirectly related to stroke volume, and the target cut-off had to be comparable with the majority of the literature. Third, we only included standard care comparators meaning that treatment was either at the discretion of the clinical team or based on standard monitoring targets such as mean arterial pressure, heart rate, central venous pressure, and/or urinary output.

For heterogeneity of outcome definitions, we did the following: when reported definitions were homogenous, all trials – including those with no reported definition – were pooled for the primary analysis; when reported definitions were heterogeneous, comparable definitions were selected from those trials that reported one. When possible, guidelines-based definitions (e.g. EPCO 2015^30^) were prioritised.

Based on data availability, several *post-hoc* subgroup analyses were performed. Subgroup analyses of abdominal *vs* non-abdominal surgery (≥50% *vs* <50%) were performed for all outcomes with ‘abdominal’ meaning any surgery within the abdominal cavity including retroperitoneal surgery. For the primary outcomes mortality and hospital length of stay, we also performed subgroup analyses by risk of surgery (‘moderate risk’, ‘high risk’, and ‘very high risk’), open *vs* laparoscopic surgery (≥50% *vs* <50%), GDHT protocol concept of preload variation (the respiratory cycle *vs* fluid challenges), GDHT protocol use of vasopressors, inotropes (none *vs* any), or both, by intraoperative fluid volume difference between the GDHT and standard care group (‘≤–500 ml’ *vs* ‘similar fluid volumes’ *vs* ‘≥500 ml’), type of device (noninvasive techniques *vs* pulse contour analysis *vs* oesophageal Doppler monitoring), and finally type of fluid (crystalloids *vs* colloids). Details on subgroup definitions are provided in Supplementary Content S2, section ‘Subgroup definitions’. For each outcome, sensitivity analyses – as described in Supplementary Content S2, section ‘Outcomes: Definitions, data synthesis, and sensitivity analyses’ – were also performed. Meta-regressions were performed for the primary outcomes mortality and hospital length of stay to evaluate effect modification by selected continuous variables. Only comparisons with at least 10 trials were considered. Selected potential modifiers were median year of patient inclusion, duration of surgery, and sample size, and control group mortality and hospital length of stay as a reflection of the illness severity in the underlying trial population. The latter two analyses should be interpreted with caution because of the potential for regression to the mean.^31^^,^^32^ Results are presented in a table and visually using bubble plots.

For binary outcomes, we conducted meta-analyses and meta-regressions using Peto's method for odds ratios (ORs) because many trials had few or zero events in one of the groups.^33^^,^^34^ Results from these analyses are reported as ORs with 95% confidence intervals (CIs) with values <1 indicating better outcomes in the GDHT group. DerSimonian and Laird random-effects meta-analyses were used for continuous outcomes. Results from these analyses are presented as mean differences with 95% CIs with values <0 indicating better outcomes in the GDHT group. To allow for meta-analyses, continuous outcomes reported as a median with a measure of variance (e.g. quartiles) were transformed to a mean and a standard deviation using the method described by Shi and colleagues.^35^ Statistical heterogeneity was assessed using forest plots and *I*^2^ statistics. To test for subgroup differences, we calculated *P*-values using Cochrane's Q statistics.^36^ For trials with multiple GDHT allocations, the number of controls was evenly spread among these groups if included in the same meta-analysis.

To assess for potential publication bias for the primary outcomes, funnel plots were created and visually interpreted.

All analyses were conducted using Stata version 17 (StataCorp LP, College Station, TX, USA).

### Confidence in cumulative evidence

The certainty of the overall evidence for a given comparison was assessed using the Grading of Recommendations Assessment, Development and Evaluation (GRADE) methodology and classified within one of four categories: very low, low, moderate, or high certainty of evidence.^37^ GRADEpro (McMaster University, 2020) was used for drafting of the GRADE tables.

## Results

### Overview

The search identified 23 454 unique records, of which 534 full-text articles were assessed for eligibility, and 95 trials were identified (eFig. 1). Six additional trials were identified in bibliographies, yielding a total of 101 trials. Three trials had two GDHT groups giving a total of 104 GDHT-allocations, which will be referred to as ‘trials’ in the following. The search for registered ongoing or unpublished trials identified 53 trials (eTable 1).

Fifteen trials compared two different GDHT protocols and did not include a standard care comparator; no meaningful meta-analyses were possible for these trials, and they are only reported descriptively (eTable 2). A further 13 trials were excluded from all meta-analyses because of incomparable interventions: non-stroke volume-related GDHT-target (*n*=7), supranormal or restrictive GDHT-targets (*n*=5), and GDHT-protocol without any fluid intervention (*n*=1) (eTable 2).

The remaining 76 trials, which compared GDHT with standard care, represented 9081 patients; from this pool, trials with comparable outcomes were included in meta-analyses. Details on the included GDHT protocols are provided in Table 1, whereas characteristics on included trials are provided in eTable 3. There was some heterogeneity among the included trials, for example in inclusion years (1988–2020), types of surgery, concepts of preload variation, and reported outcomes. Each trial's reported outcomes are presented in eTable 4.

**Table 1:** Overview of goal-directed haemodynamic therapy (GDHT) protocols. CFT, corrected flow time (s); CI, cardiac index (L min^−1^ m^−2^); CNAP, continuous noninvasive arterial pressure; CVC, central venous catheter; CVP, central venous pressure (mm Hg); DO_2_, oxygen delivery (ml min^−1^ m^−2^); E/e′ ratio, ratio between diastolic peak mitral inflow velocity and early diastolic mitral annular tissue velocity; ELWI, extravascular lung water index (ml kg^−1^); GDHT, goal-directed haemodynamic therapy; GEDWI, global end-diastolic volume index (ml m^−2^); HES, hydroxyethyl starch; HPI: Hypotension Prediction Index (score: 0–100%); HR, heart rate (min^−1^); IBP, invasive blood pressure; LiDCO, lithium dilution cardiac output; LVEDP, left ventricular end diastolic pressure (mm Hg); LVOT-VTI, left ventricular outflow tract velocity time integral (cm s^−1^); NICOM, noninvasive cardiac output monitoring; NR, not reported; O_2_ER, oxygen, extraction ratio (VO_2_ DO_2_^−1^); ODM, oesophageal Doppler monitoring; PAC, pulmonary artery catheter; PAOP, pulmonary artery occlusion pressure (mm Hg); PCA–, pulse contour analysis without calibration (devices with autocalibration included here); PCA+, pulse contour analysis with calibration (thermodilution or lithium); PI, perfusion index (%); PiCCO, pulse contour cardiac output; PPV, pulse pressure variation (%); PVI; pleth variability index (0–100%); S_cv_O_2_, mixed venous oxygen saturation (%); S_t_O_2_, oxygen saturation (%); SV, stroke volume (ml s^−1^); ΔSV, Delta stroke volume (%); SVI, stroke volume index (ml min^−1^ m^−2^); ΔSVI, Delta stroke volume index (%); SVR, systemic vascular resistance (dyn s cm^−5^); SVV, stroke volume variation (%); UO, urinary output (ml kg^−1^ or ml kg^−1^ h^−1^); VO_2_, oxygen consumption (ml min^−1^ m^−2^).

First author, year of publication	Type of device to measure target	Device brand	GDHT targets	Fluid therapy to reach target (bolus amount, type)	Vasoactive drugs to reach target
Shoemaker, 1988^38^	PAC	NR	CI 2.8–3.5 DO_2_ 400–550 VO_2_ 120–140	NR	Norepinephrine, dopamine, dobutamine
Bender, 1997^39^	PAC	NR	PAOP 8–14 CI >2.8 SVR <1100	NR, crystalloids	Dopamine, nitroprusside
Sinclair, 1997^40^	ODM	ODM2 monitor, Abbott	CFT 0.36–0.40 ΔSV <10	3 ml kg^−1^, HES	None
Conway, 2002^41^	ODM	NR	CFT >0.35 ΔSV <10	3 ml kg^−1^, HES	None
Gan, 2002^42^	ODM	Deltex	CFT >0.35 ΔSV <10	200 ml, HES	None
Venn, 2002^43^	ODM	Deltex	CFT >0.4 ΔSV <10	100–200 ml, gelatine	NR
Wakeling, 2005^44^	ODM	Cardio-Q-ODM monitor	ΔSV <10	250 ml, gelatine	None
Noblett, 2006^45^	ODM	Cardio-Q-ODM monitor	CFT >0.35 ΔSV <10	7 ml kg^−1^, gelatine	None
Lopes, 2007^46^	PCA–	IBPplus monitor	PPV <10	NR, HES	None
Buettner, 2008^47^	PCA+	PiCCO Plus monitor	SBP variation <10%	NR, crystalloid or HES	None
Harten, 2008^48^	PCA+	LiDCO plus	PPV <10	250 ml, HES	None
Senagore, 2009^49^	ODM	Deltex	ΔSV <10	200 ml, HES or 300 ml, crystalloid	None
Benes, 2010^50^	PCA–	FloTrac Vigileo 1.10, Edwards Lifesciences	SVV <10 ΔCI <10 CI >2.5	3 ml kg^−1^, HES	Dobutamine
Forget, 2010^51^	PCA–	Masimo V7.1.1.5 with Datex S/5 monitor	PVI <13	250 ml, HES	None
Mayer, 2010^52^	PCA–	FloTrac Vigileo, Edwards Lifesciences	CI >2.5 MAP >65 SVI >35 SVV <12	500 ml, crystalloid or 250 ml, colloid	Norepinephrine, dobutamine
Van der Linden, 2010^53^	PCA–	FloTrac Vigileo 1.07, Edwards Lifesciences	CI >2.5	250 ml, HES	Dobutamine
Challand, 2011^54^	ODM	Cardio-Q-ODM monitor	ΔSV <10	200 ml, HES	None
Pillai, 2011^55^	ODM	Cardio-Q-ODM monitor	ΔSV <10 CFT >0.35	3 ml kg^−1^, NR	None
Brandstrup, 2012^56^	ODM	Cardio-Q-ODM monitor	ΔSV <10	200 ml, HES	None
Zhang, 2012^57^	PCA–	Datex Ohmeda S/5 monitor	PPV <12	250 ml, crystalloid	None
Bisgaard, 2013^58^	PCA+	LIDCOplus	SVI >10	250 ml, HES	None
Bundgaard-Nielsen, 2013^59^	ODM	Cardio-Q-ODM monitor	ΔSV <10	3 ml kg^−1^, HES	None
El Sharkawy, 2013^60^	ODM	EDM	CFT >0.35 ΔSV <10	200 ml, HES	None
McKenny, 2013^61^	ODM	EDM	ΔSV <10	3 ml kg^−1^, HES	None
Ramsingh, 2012^62^	PCA–	FloTrac Vigileo 3.02, Edwards Lifesciences	SVV <13	250 ml, albumin	None
Salzwedel, 2013^63^	PCA–	ProAQT, Pulsion Medical Systems	PPV <10 CI >2.5	NR	Norepinephrine, ephedrine
Scheeren, 2013^64^	PCA–	FloTrac Vigileo, Edwards Lifesciences	SVV <10 ΔSV <10	200 ml, HES	None
Srinivasa, 2013^65^	ODM	Cardio-Q-ODM monitor	CFT 0.35–40 ΔSV <10	3–7 ml kg^−1^, colloids	None
Zakhaleva, 2013^66^	ODM	Cardio-Q-ODM monitor	CFT >0.35 ΔSV <10	3–7 ml kg^−1^, NR	None
Zheng, 2013^67^	PCA–	FloTrac Vigileo, Edwards Lifesciences	CI >2.5 SVI >35 SVV <12 MAP >65	200–250 ml colloid/500 ml crystalloid	Norepinephrine, dopamine
Pearse, 2014^68^	PCA+	LiDCO Rapid	ΔSV <10	250 ml, colloid not further specified	None
Peng, 2014^69^	PCA–	FloTrac Vigileo 3.0, Edwards Lifesciences	SVV <10	4 ml kg^−1^, HES	None
Pestaña, 2014^70^	Bioreactance	NICOM, Cheetah Medical	MAP >65 CI >2.5 ΔSV <10	250 ml, HES or gelatine	Norepinephrine, dobutamine
Phan, 2014^71^	ODM	Cardio-Q-ODM monitor	SVI >35 CFT >0.36 ΔSV <10	250 ml, HES, gelatine, or albumin	None
Shillcutt, 2014^72^	ODM	Phillips CX50	LVOT-VTI 16–25 LVEDP 5–12 E/e′ ratio 4–8 Systolic divided by diastolic pulmonary vein flow velocity >1	NR	None
Benes, 2015^73^	Noninvasive finger-pulse contour analysis	CNSystems with Ultraview SL2700 monitor, Spacelabs Healthcare	PPV <13	3 ml kg^−1^, NR	None
Colantonio, 2015^74^	PCA–	FloTrac Vigileo 1.14, Edwards Lifesciences	CI >2.5 SVI >35 SVV <15	250 ml, HES	Dopamine
Correa-Gallego, 2015^75^	PCA–	FloTrac Vigileo, Edwards Lifesciences	SVV <2 standard deviations from baseline	250 ml, albumin	None
Funk, 2015^76^	PCA–	FloTrac Vigileo, Edwards Lifesciences	SVV <13 CI >2.2 MAP >60	250 ml, HES	Norepinephrine, phenylephrine
Jammer, 2015^77^	PCA+	LiDCO rapid	ΔSV <10 SVV <10	6 ml kg^−1^, Ringer's acetate	None
Kumar, 2015^78^	PCA–	FloTrac Vigileo, Edwards Lifesciences	CI >2.5 O_2_ER ≤27 SVV <10	500 ml, crystalloid or 250 ml, HES	Norepinephrine, dopamine, dobutamine
Lai, 2015^79^	PCA+	LiDCO rapid	SVV <10 ΔSV <10	50–200 ml, gelatine	None
Broch, 2016^80^	Noninvasive finger-pulse contour analysis	Nexfin, Edwards Lifescience	PPV <10 CI >2.5	500 ml, crystalloid or colloid	Dobutamine, epinephrine, phenylephrine
Hand, 2016^81^	PCA–	FloTrac Vigileo, Edwards Lifesciences with EV-1000 monitor	MAP >75 or <10% from baseline SVV <13 CI >3 SVR >800	250 ml, NR	Dobutamine, epinephrine, phenylephrine
Kumar, 2016^82^	PCA–	FloTrac Vigileo 3.0, Edwards Lifesciences	SVV <10	NR, Lactated Ringer's, NaCl, HES	Norepinephrine
Schmid, 2016^83^	PCA+	PiCCO2, Pulsion Medical Systems	GEDVI 640–800 CI >2.5 MAP >70 ELWI <10	500 ml, HES	Norepinephrine, dobutamine
Elgendy, 2017^84^	PCA–	FloTrac Vigileo 1.14, Edwards Lifesciences	CI >2.5 SVV <12 MAP >65	3 ml kg^−1^, HES	Norepinephrine, dobutamine
Gómez-Izquierdo, 2017^85^	ODM	Cardio-Q-ODM monitor	ΔSV <10	200 ml, HES and Ringer's	None
Liang, 2017^86^	PCA–	FloTrac Vigileo, Edwards Lifesciences	SVV 8–13 DO_2_ >500	200 ml, HES	None
Luo, 2017^87^	PCA–	FloTrac Vigileo, Edwards Lifesciences	MAP >65 CI >2.5 SVV <15	200 ml, HES or gelatine	Physician's choice
Reisinger, 2017^88^	ODM	Cardio-Q-ODM monitor	ΔSVI <10	250 ml, HES	None
Stens, 2017^89^	Noninvasive finger-pulse contour analysis	ccNexfin (noninvasive)	PPV <12 CI >2.5 MAP >70	500 ml, Lactated Ringer's 250 ml, colloid subsequently	Dobutamine
Weinberg, 2017^90^	PCA–	FloTrac 4.0 with EV1000 monitor	SVV <20 MAP within 20% of baseline CI >2.0	250 ml, crystalloid	NR
Wu, 2017^91^	PCA–	FloTrac Vigileo 3.02, Edwards Lifesciences	SVV <12 CI >2.5	50 ml, HES	None
Calvo-Vecino, 2018^92^	ODM	Cardio-Q-ODM monitor	SVV <10 CI >2.5 MAP >65	250 ml, crystalloid and HES	Norepinephrine, dobutamine
Kaufmann, 2018^93^	ODM	Deltex	ΔSV <10 MAP >70 CI >2.5	200 ml, crystalloid	Norepinephrine, ephedrine
Kim, 2018^94^	PCA–	FloTrac Vigileo, Edwards Lifesciences	SVV <12 MAP ≥65 CI >2.5	200 ml, HES	Norepinephrine, ephedrine, dobutamine
Yin, 2018^95^	Bioreactance	NICOM, Cheetah Medical	CI 2.5–4.0 SVV <13	250 ml, HES	Dobutamine
Zhang, 2018^96^	PCA–	FloTrac Vigileo, Edwards Lifesciences	SVV 9–14 ΔSV <10	200 ml, HES	None
Zhao, 2018^8^	PCA–	FloTrac Vigileo, Edwards Lifesciences	SVV <13 ΔSV <10	250 ml, HES or crystalloid	None
Cesur, 2018^97^	Pleth curve analysis	Masimo Radical 7 monitor	PVI <13 MAP >65	250 ml, gelatine	Ephedrine
Davies, 2019^98^	Noninvasive finger-pulse contour analysis	ClearSight (Nexfin), Edwards Lifesciences	ΔSV <10 MAP within 30% of baseline	250 ml, Hartmanns solution or Lactated Ringer's	Phenylephrine, metaraminol, ephedrine
Godai, 2019^99^	Pleth curve analysis and PCA–	Life Scope J, Nihon Koden	PI <5 PPV <13	250 ml, HES	Phenylephrine, dobutamine
Hasanin, 2019^100^	Pleth curve analysis	GE Solar 8000 M/I monitor	PPV <13	3 ml kg^−1^, Ringer's	Ephedrine
Liu, 2019^101^	PCA–	FloTrac Vigileo, Edwards Lifesciences	SVV <13 CI 2.5–4.0	200 ml, colloid	Dobutamine
Sujatha, 2019 – SVV arm^102^	PCA–	FloTrac Vigileo 3.0, Edwards Lifesciences	SVV <13	200 ml, HES	None
Sujatha, 2019 – PVI arm^102^	Pleth curve analysis	Masimo Radical 7 monitor	PVI <13	200 ml, HES	None
Szturz, 2019^103^	ODM	Cardio-Q-ODM monitor	CI >2.5 CFT >0.33 Peak velocity >70 m^−1^	300 ml, PlasmaLyte	Norepinephrine, dobutamine
Weinberg, 2019^104^	PCA–	FloTrac 4.0 with EV1000 monitor	SVV <20 MAP within 20% of baseline CI >2.2	250 ml, crystalloid or albumin	NR
Arslan-Carlon, 2020^105^	PCA–	FloTrac Vigileo, Edwards Lifesciences	ΔSV <10 SVV <13	250 ml, crystalloid or albumin	NR
De Cassai, 2020^106^	Pleth curve analysis	MostCare-UP – Endless version	PPV <13	NR, NaCl	None
Fischer, 2020^107^	Pleth curve analysis	Masimo Radical 7 monitor	PVI <13	3 ml kg^−1^, gelatine	Ephedrine, Norepinephrine
Iwasaki, 2020^108^	PCA–	FloTrac Vigileo, Edwards Lifesciences	SVV 10–13	250–500 ml, crystalloid	None
Nicklas, 2020^109^	PCA–	ProAQT, Pulsion Medical Systems	ΔCI <15% and >baseline CI	500 ml, crystalloid or colloid	Dobutamine
Schneck, 2020^110^	PCA–	FloTrac 4.0 with EV1000 monitor	HPI <80 SVV <12 CI >baseline CI MAP <70	250 ml, HES or gelatine	Norepinephrine, dobutamine
Diaper, 2020^111^	PCA+	LiDCO	ΔSVI <10 PPV <10	250 ml, Ringerfundin or colloid	None

We were able to extract data on intraoperative fluid volume difference in 56 (74%) trials: GDHT as compared with standard care resulted in similar (within 500 ml) intraoperative fluid volumes in 35 (62%) trials, greater (>500 ml) in 10 (18%) trials, and lesser (<–500 ml) in 11 (20%) trials (eFig. 2). A total of 51 (91%) trials reported a difference within 1000 ml.

### Risk of bias

Risk of bias within the individual trials is presented in eTable 5. Risk of bias was intermediate for most trials primarily because of a lack of blinding of the clinician performing the intervention. In most trials, the risk of bias was the same across all outcomes.

### Mortality, primary result

**Fig 1:**
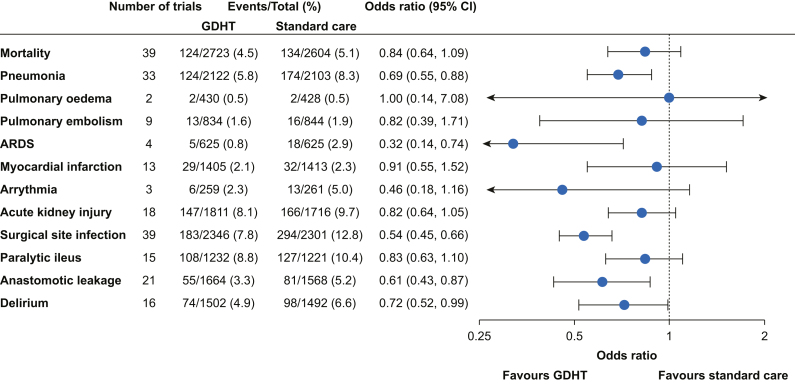
Overall results for all binary outcomes. Results from meta-analyses comparing GDHT with standard care for all binary outcomes. Estimates are odds ratios with 95% confidence intervals. Number of trials included in the analyses and patients with events *vs* totals are reported for both GDHT and standard care groups. Figures of individual forest plots are shown in eFigures 3, 6, 37, 41, 43, 45, 47, 49, 53, 55, 59, 62, and 65. ARDS, acute respiratory distress syndrome; CI, confidence interval; GDHT, goal-directed haemodynamic therapy.

Fifty (66%) of the included trials reported mortality; 39 of these also reported a time frame, of which 37 were in-hospital, 28-day, or 30-day mortality. Because of these relatively homogeneous time frames, meta-analysis was considered for all 50 trials; however, 11 trials were not included in the meta-analysis because of zero events in both groups. GDHT resulted in reduced mortality but with some imprecision (OR=0.84; 95% CI, 0.64–1.09; Fig. 1, eFig. 3). Results were similar when excluding trials with high risk of bias and when excluding two trials that only reported ICU mortality and 180-day mortality, respectively (eTable 6; eFigs 4 and 5).

### Hospital length of stay, primary result

Sixty-five (86%) of the included trials reported hospital length of stay, of which 40 had their medians and measures of variance converted to means and standard deviations. The definitions of hospital length of stay were homogeneous, and the meta-analysis thus included all 65 trials. GDHT resulted in overall shorter hospital length of stay (mean difference=–0.72 days; 95% CI, –1.10 to –0.35; eFig. 6). Results were similar when excluding trials with high risk of bias and when excluding trials with hospital length of stay >20 days in the control group (eTable 6; eFigs 7 and 8).

### Subgroup analyses, meta-regression, and funnel plots for mortality and hospital length of stay

**Fig 2:**
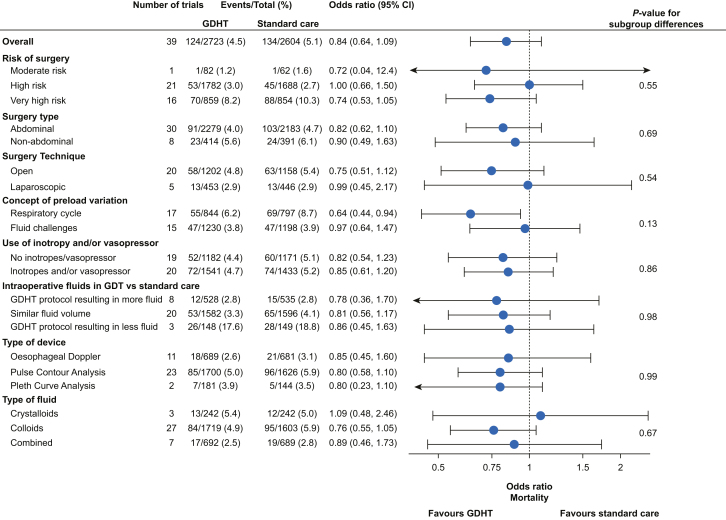
Subgroup results for mortality. Subgroup results from meta-analyses comparing GDHT with standard care for mortality. Estimates are odds ratios with 95% confidence intervals. Number of trials included in the analyses and patients with events *vs* totals are reported for both GDHT and standard care groups in all subgroups. *P*-values for formal test of subgroup differences are presented in the rightmost column. Definitions of all subgroups are provided in Supplementary Content S2. Figures of individual forest plots are shown in eFigures 9–16. CI, confidence interval; GDHT, goal-directed haemodynamic therapy.

**Fig 3:**
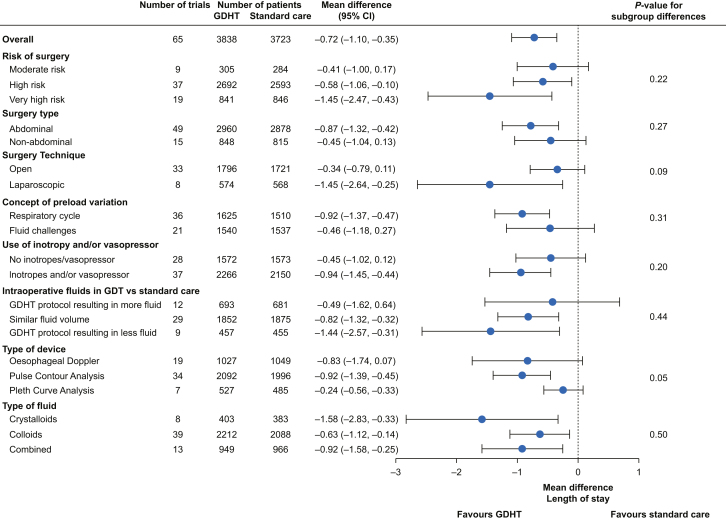
Subgroup results for hospital length of stay. Subgroup results from meta-analyses comparing GDHT with standard care for hospital length of stay. Estimates are mean differences with 95% confidence intervals. Number of trials included in the analyses and number of patients are reported for both GDHT and standard care groups in all subgroups. *P*-values for formal test of subgroup differences are presented in the rightmost column. Definitions of all subgroups are provided in Supplementary Content S2. Figures of individual forest plots are shown in eFigures 17–24. CI, confidence interval; GDHT, goal-directed haemodynamic therapy.

Subgroup analyses for mortality and hospital length of stay are summarised in Fig 2, Fig 3, respectively – a detailed description is provided in eTables 7 and 8, whereas forest plots for individual analyses are presented in eFigures 9–16 and 17–24. Results from meta-regressions for both outcomes are reported in eTable 9, whereas forest plots for individual analyses are given in eFigures 25–29 and 30–34 for mortality and hospital length of stay, respectively.

There was no clear difference in the effect of GDHT on mortality or hospital length of stay according to all subgroups (all *P*-values for subgroup differences >0.05). Estimates for very high-risk surgery, abdominal surgery, and GDHT targets based on preload variation by the respiratory cycle showed a larger effect of GDHT for both outcomes – however, all confidence intervals had considerable overlap with their comparators.

Meta-regression showed increased absolute reduction in hospital length of stay by GDHT with increasing length of stay in the control group (eFig. 34). There were no clear effect measure modifications in the remaining meta-regression analyses.

Funnel plots for mortality and hospital length of stay showed no clear signs of publication bias (eFigs 35 and 36).

### Postoperative complications

For postoperative complications, details on outcome definitions and data synthesis are given in Supplementary Content S2, section ‘Outcomes: Definitions, data synthesis, and sensitivity analyses’. A detailed summary of the results from the primary analyses and sensitivity analyses is provided in eTable 6, whereas individual forest plots are provided in eFigures 37–67.

Except for pulmonary oedema, which showed neutral results (eFig. 43), point estimates favoured GDHT for all postoperative complications. However, only pneumonia, acute respiratory distress syndrome, surgical site infection, anastomotic leakage, and delirium had estimates where the 95% CIs did not include 1 (Fig. 1). As compared with the primary analyses, there was no clear different effect of GDHT on any postoperative complication when grouped by abdominal surgery *vs* non-abdominal surgery (eFigs 37, 41, 45, 47, 49, 53, 55, 59, 62, and 65).

Sensitivity analyses on postoperative complications generally showed similar results with their primary analyses although point estimates varied, especially for outcomes with few included trials or patients (eFigs 38–40, 42, 44, 46, 48, 50–52, 54, 56–58, 60, 61, 63, 64, 66, and 67). Including all trials that reported pulmonary oedema gave a more precise and larger risk reductive effect of GDHT on the outcome but led to moderate inconsistency in the estimates (*I*^2^=47%) (eFig. 42). For delirium, a sensitivity analysis only including three trials with the EPCO 2015 definition^30^ showed a stronger effect favouring GDHT (eFig. 67).

### GRADE assessment

GRADE assessment is presented in Table 2. For all outcomes, the certainty in the evidence was downgraded owing to risk of bias. The primary outcomes mortality and hospital length of stay were classified as low level of certainty because of imprecision and inconsistent estimates, respectively. The level of certainty was moderate for pneumonia, surgical site infection, and anastomotic leakage, which all had consistent estimates favouring GDHT and relatively narrow CIs with large sample sizes. Certainties for other postoperative complications were either very low or low.

**Table 2:** GRADE (Grading of Recommendations, Assessment, Development, and Evaluations). ∗The majority of the trials were rated as having an intermediate risk of bias. ^†^The 95% confidence interval includes both potential benefit and no effect. ^‡^Substantial heterogeneity (*I*^2^=78%), but few trials showing harm. ^¶^Wide 95% confidence interval including both potential benefit and harm. ^§^Optimal information size not reached, see Supplementary Content S2. ^||^Confidence interval includes both benefit and harm. ^#^Moderate inconsistency (*I*^2^=41%). CI, confidence interval; OR, odds ratio; MD, mean difference.

Certainty assessment							Number of patients (events/total (%) or *n*)		Effect		Certainty
No. of trials	Study design	Risk of bias	Inconsistency	Indirectness	Imprecision	Other	Goal-directed therapy	Standard of care	Relative (95% CI)	Absolute (95% CI)	
Mortality											
39	RCTs	Serious∗	Not serious	Not serious	Serious^†^	None	124/2723 (4.6%)	134/2604 (5.1%)	OR 0.84 (0.64–1.09)	8 fewer per 1000 (from 18 fewer to 4 more)	⊕⊕◯ ◯ LOW
Hospital length of stay											
65	RCTs	Serious∗	Serious^‡^	Not serious	Not serious	None	3838	3723	–	MD 0.7 days fewer (1.1 fewer to 0.4 fewer)	⊕⊕◯ ◯ LOW
Pneumonia											
33	RCTs	Serious∗	Not serious	Not serious	Not serious	None	124/2122 (5.8%)	174/2103 (8.3%)	OR 0.69 (0.55–0.88)	24 fewer per 1000 (from 35 fewer to 9 fewer)	⊕⊕⊕◯ MODERATE
Pulmonary oedema											
2	RCTs	Serious∗	Not serious	Not serious	Very serious^¶^	None	2/430 (0.5%)	2/428 (0.5%)	OR 1.00 (0.14–7.08)	0 fewer per 1000 (from 4 fewer to 27 more)	⊕◯ ◯ ◯ VERY LOW
Pulmonary embolism											
9	RCTs	Serious∗	Not serious	Not serious	Very serious^¶^	None	13/834 (1.6%)	16/844 (1.9%)	OR 0.82 (0.39–1.71)	3 fewer per 1000 (from 11 fewer to 13 more)	⊕◯ ◯ ◯ VERY LOW
Acute respiratory distress syndrome											
4	RCTs	Serious∗	Not serious	Not serious	Serious^§^	None	5/625 (0.8%)	18/625 (2.9%)	OR 0.32 (0.14–0.74)	19 fewer per 1000 (from 25 fewer to 7 fewer)	⊕⊕◯ ◯ LOW
Myocardial infarction											
13	RCTs	Serious∗	Not serious	Not serious	Serious^||^	None	29/1405 (2.1%)	32/1413 (2.3%)	OR 0.91 (0.55–1.52)	2 fewer per 1000 (from 10 fewer to 11 more)	⊕⊕◯ ◯ LOW
Arrhythmia											
3	RCTs	Serious∗	Not serious	Not serious	Serious^||^	None	6/259 (2.3%)	13/261 (5.0%)	OR 0.46 (0.18–1.16)	26 fewer per 1000 (from 40 fewer to 8 more)	⊕⊕◯ ◯ LOW
Acute kidney injury											
18	RCTs	Serious∗	Serious^#^	Not serious	Serious^†^	None	147/1760 (8.4)	166/1663 (10.0)	OR 0.83 (0.65–1.06)	16 fewer per 1000 (from 33 fewer to 5 more)	⊕◯ ◯ ◯ VERY LOW
Surgical site infection											
39	RCTs	Serious∗	Not serious	Not serious	Not serious	None	183/2346 (7.8%)	294/2301 (12.8%)	OR 0.54 (0.45–0.66)	54 fewer per 1000 (from 66 fewer to 40 fewer)	⊕⊕⊕◯ MODERATE
Paralytic ileus											
15	RCTs	Serious∗	Not serious	Not serious	Serious^†^	None	108/1232 (8.8%)	127/1221 (10.4%)	OR 0.83 (0.63–1.10)	16 fewer per 1000 (from 36 fewer to 9 more)	⊕⊕◯ ◯ LOW
Anastomotic leakage											
21	RCTs	Serious∗	Not serious	Not serious	Not serious	None	55/1664 (3.3%)	81/1568 (5.2%)	OR 0.61 (0.43–0.87)	19 fewer per 1000 (from 29 fewer to 6 fewer)	⊕⊕⊕◯ MODERATE
Delirium											
16	RCTs	Serious∗	Not serious	Not serious	Serious^†^	None	74/1502 (4.9%)	98/1492 (6.6%)	OR 0.72 (0.52–0.99)	18 fewer per 1000 (from 30 fewer to 1 fewer)	⊕⊕◯ ◯ LOW

## Discussion

In this comprehensive systematic review, GDHT as compared with standard care, used during general anaesthesia for adult patients undergoing noncardiac surgery, resulted in reduced mortality and hospital length of stay – however, the estimates were imprecise and the overall certainty in the evidence was low. GDHT reduced the risk of pneumonia, surgical site infection, and anastomotic leakage (moderate certainty in evidence). Point estimates from meta-analyses also favoured GDHT for other postoperative complications but the certainty in the evidence was very low to low. Our findings support that GDHT may reduce postoperative complication rates, especially infections and anastomotic leakage, but whether it reduces mortality or shortens hospital length of stay remains uncertain and will require evidence from larger trials. Although meta-regression did not find any association between study size and effect size, it is of note that none of the trials including more than 200 patients had an overall mortality estimate favouring GDHT.

A potential mechanism of a beneficial effect of GDHT cannot be determined from the current review. We found that the average volume of fluid used in the GDHT and control groups in the included trials were relatively similar with 91% of the trials reporting a difference within 1000 ml. However, it is possible that the goals used in GDHT allow for a more individualised approach such that some patients benefit with fluid optimisation and increased cardiac output, whereas others avoid excessive fluid administration and therefore have a decreased risk of tissue oedema, which could potentially lead to a decreased risk of outcomes such as pneumonia, surgical site infection, and anastomotic leakage.

The included trials were heterogeneous. Although the authors of a previous review concluded that no meaningful meta-analysis could be conducted because of heterogeneity,^6^ we instead explored the potential importance of this heterogeneity through extensive subgroup analyses, sensitivity analyses, and meta-regression. Although there were certain population subgroups where GDHT appeared to be more beneficial (e.g. abdominal surgery, very high-risk surgery), these findings are very uncertain as formal statistical tests for subgroup differences were not statistically significant. We also explored whether heterogeneity in the GDHT protocol could influence the effect. There were some signals that GDHT protocols with targets based on preload variation by the respiratory cycle demonstrated better outcomes, but, again, this result should be interpreted carefully as the test for subgroup differences did not reach statistical significance. There were no clear indications that other elements of the GDHT protocol (e.g. use of vasoactive drugs, the amount/type of fluid) modified the effect. The results were generally consistent across multiple sensitivity analyses. Heterogeneity is unavoidable in any meta-analysis, but it is still reasonable to conduct meta-analyses as long as this heterogeneity does not influence the effect of the intervention (i.e. that there is no effect measure modification). With that said, the results of our meta-analyses should be interpreted carefully within this context. With the availability of additional larger trials in the future, it might be possible to further explore this heterogeneity and determine whether there are certain subgroups that benefit with greater certainty or whether certain elements of the GDHT protocol result in better outcomes.

Given the nature of GDHT, it is practically impossible to blind the clinical team performing the intervention. It was therefore not possible to determine whether the two treatment groups received comparable treatment outside the protocol. As such, all the included trials were rated as having an intermediate risk of bias owing to a lack of blinding of the clinical team. Future trials should focus on blinding personnel not directly involved in the intervention, including outcome assessors. Strict adherence to pre-defined outcome definitions would also lower the potential risk of bias and allow for more homogenous comparisons.

This systematic review identified 104 clinical trials assessing various aspects of GDHT, of which 76 specifically compared a GDHT protocol including fluid therapy to optimise stroke volume (or a related parameter) to standard of care. Despite this large number of trials, the 76 trials only included 9081 patients. For inclusion in meta-analyses, the number of patients ranged from 310 patients to 5406 patients depending on the outcome. This illustrates two points. First, the majority of the trials were small with only 10 trials including more than 200 patients and only one trial including more than 500 patients. Second, many trials did not report patient-centred outcomes such as mortality, hospital length of stay, and postoperative complications (eTable 4). No trial reported health-related quality of life, nor did we identify any study assessing cost-effectiveness of a GDHT protocol. Future trials should include multicentre collaborations to increase the sample size and focus on outcomes that are relevant for both clinicians and patients. Such trials are currently on the way (eTable 1).^112^, ^113^, ^114^

This review has several strengths. We conducted a comprehensive and updated search and adhered to standard methodology including risk of bias assessment and GRADE evaluation. We provide detailed information on the included trials and performed extensive subgroup and sensitivity analyses on a wide range of patient-centred outcomes to an extent not included in previous reviews.

The review also has several limitations. As noted, the included trials were generally small and heterogeneous with many trials reporting zero or few outcome events. This makes valid meta-analyses difficult.^33^^,^^34^ Also, continuous outcomes reported as a median with a measure of variance (e.g. quartiles) were transformed to a mean and a standard deviation, which could result in some mis-estimation. The relatively low number of included patients also limits our statistical power, especially for subgroup analyses and meta-regressions. Lastly, given the many small trials and sometimes poor reporting, it was difficult to identify all relevant trials as reflected in a relatively low agreement between reviewers. Although we also reviewed references of included trials, previous systematic reviews, and trial registration sites, it is possible that we might have missed some relevant trials.

In adult noncardiac surgery, GDHT during general anaesthesia might reduce mortality, hospital length of stay, and the risk of several postoperative complications. However, although a reduction in pneumonia, surgical site infection, and anastomotic leakage reached moderate certainty in the evidence, it was very low or low for most outcomes.

## Authors' contributions

Study conception and design: LWA, AG, MJH

Data acquisition: all authors.

Data analysis: MKJ, MFV, LWA

Data interpretation: all authors.

Drafting the manuscript: MKJ, MFV, LWA

Critical revision of the manuscript for important intellectual content: all authors.

All authors reviewed the results and approved the final version of the manuscript.

## Declaration of interest

None of the authors have any conflicts of interests.

## Protocol registration

Version 1 (June 11, 2020): https://doi.org/10.6084/m9.figshare.12464366.v1.

Version 2 (August 19, 2020): https://doi.org/10.6084/m9.figshare.12826595.v1.
