# Supplementary material for: Goal-directed haemodynamic therapy during general anaesthesia for noncardiac surgery: a systematic review and meta-analysis
Source: Br J Anaesth. 2021 Dec 13;128(3):416–33. doi: 10.1016/j.bja.2021.10.046 (PMC8900265; doi:10.1016/j.bja.2021.10.046)
Supplement: Multimedia component 1 [file mmc1.docx]

SUPPLEMENTARY CONTENT S2

Goal-Directed Haemodynamic Therapy During General Anaesthesia for Non-Cardiac Surgery: A Systematic Review and Meta-Analysis

# CONTENT

[CONTENT 2](#_Toc82011947)

[SUPPLEMENTAL METHODS 8](#_Toc82011948)

[Ongoing randomized clinical trials 8](#_Toc82011949)

[Risk of bias assessment 8](#_Toc82011950)

[Risk of bias arising from the randomization process 8](#_Toc82011951)

[Risk of bias due to deviations from the intended interventions 9](#_Toc82011952)

[Risk of bias due to missing outcome data 9](#_Toc82011953)

[Risk of bias in measurement of the outcome 9](#_Toc82011954)

[Risk of bias in selection of the reported result 9](#_Toc82011955)

[Overall risk of bias 10](#_Toc82011956)

[Pooling of trials based on heterogeneity 10](#_Toc82011957)

[Outcomes: Definitions, data synthesis, and sensitivity analyses 11](#_Toc82011958)

[Intraoperative fluid volume difference 11](#_Toc82011959)

[Mortality 11](#_Toc82011960)

[Hospital length of stay 12](#_Toc82011961)

[Pneumonia 12](#_Toc82011962)

[Pulmonary oedema 12](#_Toc82011963)

[Pulmonary embolism 13](#_Toc82011964)

[Acute respiratory distress syndrome 13](#_Toc82011965)

[Myocardial infarction 13](#_Toc82011966)

[Arrhythmia 14](#_Toc82011967)

[Acute kidney injury 14](#_Toc82011968)

[Surgical site infection 14](#_Toc82011969)

[Paralytic ileus 15](#_Toc82011970)

[Anastomotic leakage 15](#_Toc82011971)

[Delirium 15](#_Toc82011972)

[Considerations for outcomes that were data extracted but not analysed 16](#_Toc82011973)

[Outcome definition of acute lung injury (ALI) 16](#_Toc82011974)

[Outcome definition of combined pulmonary complications 16](#_Toc82011975)

[Outcome definition of combined cardiac complications 16](#_Toc82011976)

[Outcome definition of combined abdominal complications 16](#_Toc82011977)

[Subgroup definitions 16](#_Toc82011978)

[Risk of surgery 16](#_Toc82011979)

[Abdominal vs. non-abdominal 17](#_Toc82011980)

[Open surgery vs. laparoscopic surgery 17](#_Toc82011981)

[Concept of preload variation 17](#_Toc82011982)

[Use of inotropes and/or vasopressor 18](#_Toc82011983)

[Intraoperative fluid amount differences between GDHT and standard care 18](#_Toc82011984)

[Type of device 18](#_Toc82011985)

[Type of fluid 19](#_Toc82011986)

[GRADE 19](#_Toc82011987)

[Risk of bias 19](#_Toc82011988)

[Inconsistency 19](#_Toc82011989)

[Indirectness 19](#_Toc82011990)

[Imprecision 19](#_Toc82011991)

[Others 20](#_Toc82011992)

[eTABLES 21](#_Toc82011993)

[eTable 1: Registered randomized controlled trials comparing goal directed therapies International Clinical Trials Registry Platform and ClinicalTrials.gov 21](#_Toc82011994)

[eTable 2: Trials not included in meta-analyses with reason for non-inclusion and trial characteristics 30](#_Toc82011995)

[eTable 3: Characteristics of included trials 36](#_Toc82011996)

[eTable 4: Reported outcomes in trials 50](#_Toc82011997)

[eTable 5: Risk of bias for individual trials 56](#_Toc82011998)

[eTable 6. Primary analyses and sensitivity analyses 64](#_Toc82011999)

[eTable 7: Subgroup analyses for mortality 67](#_Toc82012000)

[eTable 8: Subgroup analyses for hospital length of stay 69](#_Toc82012001)

[eTable 9: Meta-regression analyses 71](#_Toc82012002)

[eFIGURES 72](#_Toc82012003)

[eFigure 1: Flow chart of study selection 72](#_Toc82012004)

[eFigure 2: Intraoperative fluid volume differences between GDHT and standard care 73](#_Toc82012005)

[Mortality, primary results 74](#_Toc82012006)

[eFigure 3: Mortality - Primary analysis 74](#_Toc82012007)

[eFigure 4: Mortality - Sensitivity analysis 1: Excluding trials with high risk of bias 75](#_Toc82012008)

[eFigure 5: Mortality - Sensitivity analysis 2: Excluding outlying outcome definition 76](#_Toc82012009)

[Hospital length of stay, primary results 77](#_Toc82012010)

[eFigure 6: Hospital length of stay - Primary analysis 77](#_Toc82012011)

[eFigure 7: Hospital length of stay - Sensitivity analysis 1: Excluding trials with high risk of bias 78](#_Toc82012012)

[eFigure 8: Hospital length of stay - Sensitivity analysis 2: Excluding trials with >20 days hospital length of stay in control group 79](#_Toc82012013)

[Subgroup analyses, meta regressions, and funnel plots for mortality and hospital length of stay 80](#_Toc82012014)

[eFigure 9: Mortality - Subgroup analysis 1: Risk of surgery 80](#_Toc82012015)

[eFigure 10: Mortality - Subgroup analysis 2: Abdominal surgery vs. non-abdominal surgery 81](#_Toc82012016)

[eFigure 11: Mortality - Subgroup analysis 3: Open surgery vs. laparoscopic surgery 82](#_Toc82012017)

[eFigure 12: Mortality - Subgroup analysis 4: GDHT-targets based on preload variation by the respiratory cycle vs. fluid challenges 83](#_Toc82012018)

[eFigure 13: Mortality - Subgroup analysis 5: GDHT-protocols without vs. with use of inotropes or vasopressors 84](#_Toc82012019)

[eFigure 14: Mortality - Subgroup analysis 6: GDHT-protocols resulting in less vs. similar vs. more intraoperative fluid amounts than their standard care comparator 85](#_Toc82012020)

[eFigure 15: Mortality -Subgroup analysis 7: Type of device 86](#_Toc82012021)

[eFigure 16: Mortality - Subgroup analysis 8: Type of fluid to reach target 87](#_Toc82012022)

[eFigure 17: Hospital length of stay - Subgroup analysis 1: Risk of surgery 88](#_Toc82012023)

[eFigure 18: Hospital length of stay - Subgroup analysis 2: Abdominal surgery vs. non-abdominal surgery 89](#_Toc82012024)

[eFigure 19: Hospital length of stay - Subgroup analysis 3: Open surgery vs. laparoscopic surgery 90](#_Toc82012025)

[eFigure 20: Hospital length of stay - Subgroup analysis 4: GDHT-targets based on preload variation by the respiratory cycle vs. fluid challenges 91](#_Toc82012026)

[eFigure 21: Hospital length of stay - Subgroup analysis 5: GDHT-protocols without vs. with use of inotropes or vasopressors 92](#_Toc82012027)

[eFigure 22: Hospital length of stay - Subgroup analysis 6: GDHT-protocols resulting in less vs. similar vs. more intraoperative fluid amounts than their standard care comparator 93](#_Toc82012028)

[eFigure 23: Hospital length of stay - Subgroup analysis 7: Type of device 94](#_Toc82012029)

[eFigure 24: Hospital length of stay - Subgroup analysis 8: Type of fluid to reach target 95](#_Toc82012030)

[eFigure 25: Mortality - Meta-regression 1: Median year of patient inclusion 96](#_Toc82012031)

[eFigure 26: Mortality - Meta-regression 2: Duration of surgery 97](#_Toc82012032)

[eFigure 27: Mortality - Meta-regression 3: Sample size in trials 98](#_Toc82012033)

[eFigure 28: Mortality - Meta-regression 4: Mortality in the control group 99](#_Toc82012034)

[eFigure 29: Mortality - Meta-regression 5: Hospital length of stay in the control group 100](#_Toc82012035)

[eFigure 30: Hospital length of stay - Meta-regression 1: Median year of patient inclusion 101](#_Toc82012036)

[eFigure 31: Hospital length of stay - Meta-regression 2: Duration of surgery 102](#_Toc82012037)

[eFigure 32: Hospital length of stay - Meta-regression 3: Sample size in trials 103](#_Toc82012038)

[eFigure 33: Hospital length of stay - Meta-regression 4: Mortality in the control group 104](#_Toc82012039)

[eFigure 34: Hospital length of stay - Meta-regression 5: Hospital length of stay in the control group 105](#_Toc82012040)

[eFigure 35: Mortality - Funnel plot 106](#_Toc82012041)

[eFigure 36: Hospital length of stay - Funnel plot 107](#_Toc82012042)

[Pneumonia 108](#_Toc82012043)

[eFigure 37: Pneumonia - Primary analysis with abdominal surgery vs. non-abdominal surgery 108](#_Toc82012044)

[eFigure 38: Pneumonia - Sensitivity analysis 1: Excluding trials with high risk of bias 109](#_Toc82012045)

[eFigure 39: Pneumonia - Sensitivity analysis 2: Excluding trials without an outcome definition 110](#_Toc82012046)

[eFigure 40: Pneumonia - Sensitivity analysis 3: Only including trials with established definitions 111](#_Toc82012047)

[Pulmonary oedema 112](#_Toc82012048)

[eFigure 41: Pulmonary oedema - Primary analysis 112](#_Toc82012049)

[eFigure 42: Pulmonary oedema - Sensitivity analysis 1: All trials reporting the outcome 112](#_Toc82012050)

[Pulmonary embolism 113](#_Toc82012051)

[eFigure 43: Pulmonary embolism - Primary analysis with abdominal surgery vs. non-abdominal surgery 113](#_Toc82012052)

[eFigure 44: Pulmonary embolism - Sensitivity analysis 1: Excluding trials without an outcome definition 114](#_Toc82012053)

[Acute respiratory distress syndrome 115](#_Toc82012054)

[eFigure 45: Acute respiratory distress syndrome - Primary analysis 115](#_Toc82012055)

[eFigure 46: Acute respiratory distress syndrome – Sensitivity analysis 1: All trials reporting the outcome 116](#_Toc82012056)

[Myocardial infarction 117](#_Toc82012057)

[eFigure 47: Myocardial infarction - Primary analysis with abdominal surgery vs. non-abdominal surgery 117](#_Toc82012058)

[eFigure 48: Myocardial infarction - Sensitivity analysis 1: Excluding trials without an outcome definition 118](#_Toc82012059)

[Arrhythmia 119](#_Toc82012060)

[eFigure 49: Arrhythmia - Primary analysis 119](#_Toc82012061)

[eFigure 50: Arrhythmia – Sensitivity analysis 1: Definition “requiring intervention” 120](#_Toc82012062)

[eFigure 51: Arrhythmia - Sensitivity analysis 2: Definition “requiring intervention” excluding trials with high risk of bias 121](#_Toc82012063)

[eFigure 52: Arrhythmia - Sensitivity analysis 3: All trials reporting the outcome 122](#_Toc82012064)

[Acute kidney injury 123](#_Toc82012065)

[eFigure 53: Acute kidney injury - Primary analysis with abdominal surgery vs. non-abdominal surgery 123](#_Toc82012066)

[eFigure 54: Acute kidney injury - Sensitivity analysis 1: All trials reporting the outcome 124](#_Toc82012067)

[Surgical site infection 125](#_Toc82012068)

[eFigure 55: Surgical site infection - Primary analysis with abdominal surgery vs. non-abdominal surgery 125](#_Toc82012069)

[eFigure 56: Surgical site infection - Sensitivity analysis 1: Excluding trials with high risk of bias 126](#_Toc82012070)

[eFigure 57: Surgical site infection - Sensitivity analysis 2: Excluding trials without an outcome definition 127](#_Toc82012071)

[eFigure 58: Surgical site infection - Sensitivity analysis 3: Only including trials with established definitions 128](#_Toc82012072)

[Paralytic ileus 129](#_Toc82012073)

[eFigure 59: Paralytic ileus - Primary analysis 129](#_Toc82012074)

[eFigure 60: Paralytic ileus - Sensitivity analysis 1: Excluding trials with high risk of bias 130](#_Toc82012075)

[eFigure 61: Paralytic Ileus - Sensitivity analysis 2: All trials reporting the outcome 131](#_Toc82012076)

[Anastomotic leakage 132](#_Toc82012077)

[eFigure 62: Anastomotic leakage - Primary analysis 132](#_Toc82012078)

[eFigure 63: Anastomotic leakage - Sensitivity analysis 1: Excluding trials with high risk of bias 133](#_Toc82012079)

[eFigure 64: Anastomotic leakage - Sensitivity analysis 2: Only including trials with a definition based on radiology 133](#_Toc82012080)

[Delirium 134](#_Toc82012081)

[eFigure 65: Delirium - Primary analysis with abdominal surgery vs. non-abdominal surgery 134](#_Toc82012082)

[eFigure 66: Delirium - Sensitivity analysis 1: Excluding trials with high risk of bias 135](#_Toc82012083)

[eFigure 67: Delirium - Sensitivity analysis 2: Only including trials with EPCO 2015-definition 136](#_Toc82012084)

[PRISMA-checklist 137](#_Toc82012085)

[REFERENCES 140](#_Toc82012086)

# SUPPLEMENTAL METHODS

## Ongoing randomized clinical trials

The International Clinical Trials Registry Platform (ICTRP) was searched on April 5, 2021, and on June 28, 2021. The searches yielded 4034 records of which 53 records were ongoing randomized controlled trials comparing goal directed therapies during general anaesthesia.

To optimize sensitivity, we performed an additional search for the term *general anesthesia* (filters: recruiting, not yet recruiting, enrolling by invitation, active but not recruiting, and interventional study type) on ClinicalTrials.Gov on April 5, 2021. Of 311 records, we identified 1 additional randomized controlled trial.

We did not consider records for which 1) the recruitment status was unknown, 2) the trial status was marked as unknown for more than 5 years, 3) the trial was marked as completed for more than 5 years, and/or 4) the last update was posted over 5 years ago.

Search at ICTRP on April 5, 2021:

*anesthesia AND hemodynamic OR anesthesia AND respiratory OR surgery AND hemodynamic OR surgery AND respiratory*

Search at ICTRP on June 28, 2021:

*((anesthesia OR anaesthesia OR surgery OR surgical) AND (arterial blood pressure OR heart rate OR cardiac output OR stroke volume OR pulse pressure OR goal directed therapy OR goal-directed therapy OR oxygen OR carbon dioxide OR tidal volume OR respiratory rate OR PEEP OR recruitment maneuver OR lung protective ventilation))*

## Risk of bias assessment

Risk of bias was assessed using version 2 of the Cochrane Risk-of-Bias tool for individually-randomized parallel-group trials.^1^ Results for risk of bias are presented in eTable 5. Some general considerations related to this specific review are provided below.

### Risk of bias arising from the randomization process

Three elements are considered within this domain: random allocation sequence, allocation concealment, and baseline imbalances. In general, if randomization was described and there was no indication of loss of allocation concealment, we categorized the trial as low risk of bias – this included trials with no clear description of allocation concealment as we then assumed it to be present. If the randomization process was not described at all, we generally considered trials to be at an intermediate risk of bias.

### Risk of bias due to deviations from the intended interventions

This domain focuses on whether participants received the intended intervention without other differences in care. As such, the domain primarily focuses on blinding of participants and the clinical team. By the nature of GDHT, it is impossible to blind the clinician who provides the intervention, and for much of the treatment data it is infeasible to decipher whether differences result from the intervention or non-protocol treatments. We therefore decided to judge all trials as at least intermediate risk of bias.

The difference between the effect of assignment to an intervention and the effect of adherence to an intervention was difficult to assess and often not described in the trials. Although we were technically interested in the effect of adherence to the intervention, the distinction between the two was therefore not considered further. Since appropriate per-protocol analyses were rarely performed, we – when possible – included results from (modified) intention-to-treat analyses.

### Risk of bias due to missing outcome data

For most trials, outcomes were only assessed in-hospital, and the amount of missing data was low. These trials were therefore classified as having a low risk of bias. In a few trials, loss to follow-up was more substantial. When this loss to follow-up was moderate and equal among groups, the risk of bias was assessed as intermediate; if the loss to follow-up was more substantial and different between groups, the risk of bias was classified as high.

### Risk of bias in measurement of the outcome

This domain includes multiple elements including whether the method of measuring the outcome was appropriate and similar between groups, whether outcome assessors were aware of the intervention, and whether this knowledge could have influenced the measurement of the outcome.

For most trials, the methods of measuring the outcomes were described very limited. Survival, and hospital length of stay were considered objective outcomes, why knowledge of the intervention is unlikely to influence measurement of the outcome, and risk of bias was therefore considered low. For postoperative complications, the risk of bias was classified as low if the outcomes assessors were blinded and intermediate if they were not. We considered it unlikely that this non-blinding would have a major influence on the measurement of the outcome.

### Risk of bias in selection of the reported result

If the trials reported results consistent with a prospectively registered protocol or trial registration, this domain was classified as low risk of bias. If there was no protocol or trial registration, the trial was classified as intermediate risk of bias. If the protocol or trial registration was made retrospectively (i.e. after including the last patient), the trial was classified as intermediate risk of bias. Outcome-relevant discrepancies between the manuscript and the protocol or trial registration were classified as intermediate or high based on whether or not it was selected from multiple outcome measurements or analyses of the data.

### Overall risk of bias

The overall risk of bias was generally based on the highest risk reported within an individual domain.

## Pooling of trials based on heterogeneity

We assessed all included trials for clinical and methodological heterogeneity. First, we looked at trial design, population, intervention, and comparator in order to compose a main pool of trials that was considered comparable on these parameters. These trials were then assessed for heterogeneity of outcome definitions (see section “Outcomes: Definitions, data synthesis, and sensitivity analyses”).

This review only included randomized controlled trials. This also comprised quasi-randomized trials (e.g., assignment based on day of the week) as well as cluster-randomized trials. Randomized crossover trials where the crossover occurred within individual patients were not included. Observational studies, case series, case reports, reviews, abstracts, editorials, comments, letters to the editor, or unpublished studies were not included. We did not assess further heterogeneity of study design.

This review included trials that investigated adult patients undergoing non-cardiac surgery in general anaesthesia with mechanical ventilation. We did not exclude trials based on heterogeneity of the population but did assess further complexity of this in three subgroup analyses (type and risk of surgery, see section “Subgroup definitions”) and three meta-regressions (duration of surgery and mortality as well as hospital length of stay in the control group).

As described in the main manuscript and eTable 2, we made several exclusions from the main pool of trials due to heterogeneity of the GDHT-protocols. Ultimately, all included GDHT-protocols included an intraoperative fluid intervention based on a GDHT-target that was either directly or indirectly related to stroke volume. Additionally, all cut-offs for included targets were comparable. We assessed further complexity of the intervention in five subgroups (concept of preload variation, use of inotropes and/or vasopressors, type of device as well as type and amount of fluid therapy).

Likewise, we made several exclusions based on the comparator (eTable 2). Ultimately, all comparators were considered standard care meaning either at the discretion of the clinical team or based on standard monitoring targets such as mean arterial pressure (MAP), heart rate (HR), central venous pressure (CVP), and/or urinary output. We did not assess further heterogeneity of the comparator.

## Outcomes: Definitions, data synthesis, and sensitivity analyses

When an outcome was reported with comparable definitions, the primary analysis consisted of data from all trials that reported the outcome – including trials that did not report a definition. When outcome definitions were heterogeneous, we only considered trials with an outcome definition; the primary analysis was then made with a pool of trials that had comparable outcome definitions. For this, we prioritized standardized definitions such as EPCO^2^, CDC^3^, POSSUM^4^ and NSQIP^5^ over the authors’ own definitions – secondarily, we prioritized statistical power, i.e., sample size.

Details on considerations for data abstraction and outcome definition heterogeneity for each outcome are presented below.

### Intraoperative fluid volume difference

Intraoperative fluid volumes were extracted from all trials as total intraoperative fluids, crystalloids, and colloids, separately. For trials only reporting medians with a measurement of variance, these were converted to means and standard deviations using the updated method by Shi et al.^6^ We defined intraoperative total fluid amount as the reported intraoperative sum of crystalloids and colloids; means were summed and a new standard deviation (SD) was calculated by the formula ${SD}_{total}=\sqrt{{SD}_{crystalloid}^{2}+{SD}_{colloid}^{2}}$. If either the intraoperative amount of crystalloids or colloids was not reported, then we used the authors’ definition of total; if this was also not reported, then the outcome was not reported for the trial. Any other time frame than intraoperative was excluded. Trials reporting fluid amounts in mL/kg or mL/kg/h were not included.

Results are reported in eFigure 2.

### Mortality

Mortality was defined as all-cause mortality. If the study reported multiple mortality time frames, the priority was: 30-day > in-hospital > any other time frame. When considering outcome definitions, we considered the time frames in-hospital-, 28-day-, and 30-day mortality comparable as representing short-term mortality; 37 of 39 reported time frames were one of these definitions, and the outcome definitions were hence homogeneous. Therefore, all trials reporting mortality – including those with no clear time frame – were included in meta-analyses, including two trials reporting intensive care unit (ICU)- and 180-day mortality, respectively. Sensitivity analyses were performed for the following: 1) excluding trials with high risk of bias and 2) excluding one trial^7^ reporting 180-day mortality and one trial reporting only ICU-mortality^8^.

Results of the primary analysis are given in eFigure 3, while results of sensitivity analyses are given in eFigure 4 and 5.

### Hospital length of stay

We accepted authors’ definitions of hospital length of stay requiring no detail explaining the time frame, therefore accepting that there may be minor differences in including the preoperative period or not as well as the noted day of discharge related to time of day when discharged. We included trials which only reported time until “fit for discharge” but excluded other time frames such as ICU length of stay. 65 (86%) of the included trials reported hospital length of stay of which 40 only reported medians with a measure of variance. Sensitivity analyses were performed for the following: 1) excluding trials with high risk of bias and 2) excluding trials with hospital length of stay >20 days in the control group.

Results of the primary analysis are given in eFigure 6, while results of sensitivity analyses are given in eFigure 7 and 8.

### Pneumonia

35 trials reported pneumonia as an outcome; 10 of these used guideline definitions of which six were the Center for Disease Control (CDC) 2008-criteria^3^ (including EPCO 2015^2^ and NSQIP 2014^5^ which use identical criteria). CDC 2008-criteria for pneumonia are 1) radiological findings and 2) clinical pulmonary symptoms along with 3) either fever, leukocytosis, or an altered mental state. Two trials used ATS 2005^9^-criteria which were similar to CDC 2008-criteria. Another nine trials used their own definitions, most of them incorporating radiological findings with clinical signs or leukocytosis. We deemed these definitions comparable to guideline definitions and therefore all 35 trials – including 16 without an outcome definition – were considered for meta-analyses. Two trials were post-hoc excluded from analyses due to no events in either group.

Based on the above considerations, three sensitivity analyses were defined: 1) excluding trials with high risk of bias, 2) excluding trials without an outcome definition (n=19 as all trials that reported an outcome had one or more events in either group), and 3) only including studies with CDC 2008-, EPCO 2015-, NSQIP 2014- or ATS 2005-criteria.

Results on the primary analysis are given in eFigure 37, while results on sensitivity analyses are given in eFigure 38-40.

### Pulmonary oedema

27 trials reported pulmonary oedema of which only one trial used a guideline definition. Eight trials used their own definition of pulmonary oedema, and these were considered too heterogeneous for meta-analysis. Of the nine trials using a definition, we found “radiology and clinical signs” the most comparable and with a relevant sample size; two trials used this definition, and these comprised the primary analysis.

None of the included trials had high risk of bias. We conducted one sensitivity analysis including all 27 articles that reported the outcome (of those, six trials were excluded due to no events in either group). Results on the primary analysis are given in eFigure 41, while results on the sensitivity analysis are given in eFigure 42.

### Pulmonary embolism

20 trials reported pulmonary embolism. Five trials used guidelines definitions, while another four trials used their own definition – common for all definitions was the need of radiological findings. Therefore, we deemed the definitions comparable, and all 20 trials were considered for meta-analysis including 11 trials with no outcome definition. A different 11 trials were excluded post-hoc because of no events in either group.

None of the included trials had high risk of bias. We conducted one sensitivity analyses: excluding trials without a specified outcome definition (n=3 since six trials that reported an outcome definition had no events in either group). Results of the primary analysis are given in eFigure 43, while results of the sensitivity analysis are given in eFigure 44.

### Acute respiratory distress syndrome

11 trials reported acute respiratory distress syndrome. Seven trials used their own definitions which were too heterogeneous to be considered for meta-analysis. However, a different four trials used either EPCO 2015-criteria^2^ or the “Berlin”-definition^10^ which were identical; these were included for meta-analysis.

None of the four trials had high risk of bias. We conducted one sensitivity analysis including all 11 articles that reported the outcome (of those, three trials were excluded due to no events in either group). Results on the primary analysis are given in eFigure 45, while results of the sensitivity analysis are given in eFigure 46.

### Myocardial infarction

25 trials reported myocardial infarction. Three trials used the EPCO 2015^2^ or the AHA 2012^11^ definitions which were identical: elevated cardiac biomarker with either 1) clinical symptoms, 2) electrocardiography changes, 3) radiologic evidence of myocardium loss, or 4) coronary thrombus as evident by angiographic imaging or autopsy. Another trial used the NSQIP^5^-guideline which was similar. Six trials used their own definitions which all included an elevated cardiac biomarker, and five of these also included electrocardiography changes. We therefore considered all definitions comparable, and all 25 trials – including 15 without an outcome definition – were considered for meta-analysis. 12 trials were post-hoc excluded due to no events in either group.

None of the included trials had high risk of bias. We conducted one sensitivity analysis excluding trials without an outcome definition (n=7 since three trials that reported an outcome definition had no events in either group). Results on the primary analysis are given in eFigure 47, while results on the sensitivity analysis are given in eFigure 48.

### Arrhythmia

37 trials reported arrhythmia in both groups. Three trials used the EPCO 2015^2^-criteria, and another 15 trials used their own definitions; generally, the definitions were very heterogeneous. We decided to define two distinct analyses using 1) EPCO 2015-criteria and 2) five trials defining arrhythmia solely by the need for medical or defibrillation intervention. For the latter definition, we conducted one sensitivity analysis excluding trials with risk of bias, while none of the trials using EPCO 2015-criteria had high risk of bias. Finally, a sensitivity analysis of all 37 trials reporting arrhythmia was conducted (of those, three trials were excluded due to no events in either group).

Results on the primary analysis are given in eFigure 49, results on the secondary analysis are given in eFigure 50, and results on the sensitivity analyses are given in eFigure 51 and 52.

### Acute kidney injury

44 trials reported acute kidney injury in both groups. 15 trials used guideline definitions of which 13 adhered to either RIFLE^12^, KDIGO 2012^13^, AKIN, or EPCO2015^2^ which were comparable using a 150% increase in plasma creatinine as one of their criteria. All of these definitions include multiple categories, but most trials reported the outcome as binary without defining the cut-off – a complexity that therefore could not be assessed any further. Another 16 trials used their own definitions with varying cut-offs; of these, six trials were comparable to the guidelines’ criteria. Therefore, a total of 19 trials were considered for meta-analysis. One trial was excluded post-hoc due to no events in either group.

None of the included trials had high risk of bias. A sensitivity analysis was conducted including all trials reporting the outcome. Results on the primary analysis are given in eFigure 53, while results on the sensitivity analysis are given in eFigure 54.

### Surgical site infection

42 trials reported surgical site infection in both groups. Eight trials used guideline definitions, and another 11 trials used their own definitions; all of these definitions were comparable reflecting that surgical site infection is a clinical diagnosis with clear symptoms and objective signs. Therefore, all 42 trials – including 23 trials without an outcome definition – were considered for meta-analysis. Three trials were post-hoc excluded due to no events in either group.

Three sensitivity analyses were conducted: 1) excluding trials with high risk of bias, 2) excluding trials without an outcome definition (n=18 since one trial that reported an outcome definition had no events in either group), and 3) only including five studies using the identical EPCO 2015^2^ / NSQIP 2014^5^ / CDC 2008-guideline^3^ definitions. Results of the primary analysis are given in eFigure 55, while results of sensitivity analyses are given in eFigure 56-57.

### Paralytic ileus

23 trials reported ileus in both groups. One trial reported mechanical ileus exclusively, while 16 trials reported paralytic ileus; six trials did not state the type of ileus. We considered paralytic ileus the most clinically relevant postoperative ileus type, why we chose to consider the 16 trials reporting this as our primary analysis. One trial used a guideline definition, while another seven trials used their own definitions. All definitions were comparable incorporating missing oral intake, defecation, or flatus for ≥3 or ≥5 days. Therefore, all 16 trials – including eight trials without an outcome definition – were considered for meta-analysis. One trial was post-hoc excluded due to no events in either group.

We performed two sensitivity analyses: 1) excluding trials with high risk of bias and 2) including all trials reporting the outcome. Results on the primary analysis are given in eFigure 59, while results on the sensitivity analyses are given in eFigure 60 and 61.

### Anastomotic leakage

21 trials in abdominal surgery reported anastomotic leakage in both groups. Two trials used the EPCO 2015-criteria^2^, while another seven trials used their own definition. Definitions either required radiological evidence or reoperation which we considered comparable as most postoperative anastomotic leakages require surgical intervention. Therefore, all 21 trials – including 12 trials without an outcome definition – were considered for meta-analysis.

We performed two sensitivity analyses: 1) excluding trials with high risk of bias and 2) only including trials with a definition based on radiology. Results on the primary analysis are given in eFigure 62, while results on the sensitivity analyses are given in eFigure 63 and 64.

### Delirium

18 trials reported delirium in both groups. Three trials used the EPCO 2015^2^-criteria, while another five trials used their own definitions. We considered definitions comparable reflecting that delirium is a clinical diagnosis; however, one trial that reported psychiatric complications as a combined outcome was excluded. The remaining 17 trials – including nine trials without an outcome definition – were considered for meta-analysis. One trial was post-hoc excluded due to no events in either group.

We performed two sensitivity analyses: 1) excluding trials with a high risk of bias and 2) only including trials with the EPCO 2015-criteria^2^. Results on the primary analysis are given in eFigure 65, while results on sensitivity analyses are given in eFigure 66 and 67.

## Considerations for outcomes that were data extracted but not analysed

### Outcome definition of acute lung injury (ALI)

A few trials reported acute lung injury, while some did report “respiratory failure” or “respiratory insufficiency”. The definitions were regarded too heterogeneous for comparison, and the outcome was not included in meta-analyses.

### Outcome definition of combined pulmonary complications

Several trials reported a combined pulmonary outcome for example including pneumonia, atelectasis, pleural effusion, pneumothorax, bronchospasm, aspiration, respiratory failure, hypoxia, requirement of invasive/non-invasive ventilation etc. The definitions, if reported, were extracted and compared. The definitions were regarded too heterogeneous for comparison, and the outcome was not included in meta-analyses.

### Outcome definition of combined cardiac complications

16 trials reported a “combined cardiac complication”-outcome including for example angina, hypotension, death, MI, arrhythmia etc., while several did not report a definition. There were no comparable definitions, and the outcome was not included in meta-analyses.

### Outcome definition of combined abdominal complications

Seven trials reported a “combined abdominal” outcome. There were no comparable definitions, and the outcome was not included in meta-analyses.

## Subgroup definitions

We defined subgroups as listed below:

### Risk of surgery

Moderate risk of surgery:

- American Society of Anesthesiologists (ASA)-class I >50% AND mortality* ≤2%
- Duration of surgery* ≤120 min AND mortality* ≤2%
- Hospital length of stay* ≤5 days AND mortality* ≤2%

*Mean combined between the GDHT- and standard care group

High risk of surgery:

- Fulfilling none of the criteria for moderate risk of surgery AND none the criteria for very high risk of surgery

Very high risk of surgery:

- ASA-class III + IV >50%
- Expected post-operative ICU-admission (inclusion criteria)
- Hospital length of stay* >20 days
- Mortality* >10%

*Mean combined between the GDHT- and standard care group

### Abdominal vs. non-abdominal

We defined trials as abdominal if ≥50% of included patients underwent abdominal surgery, meaning within the abdominal cavity; this included surgeries that were retroperitoneal (e.g. kidney transplant) and there were no limits on surgical specialty. Trials were classified as non-abdominal when <50% of included patients underwent abdominal surgery. One trial not reporting surgery type was not included.

### Open surgery vs. laparoscopic surgery

This subgroup only considered trials with ≥50% abdominal surgery as defined above. We classified trials as open when <50% of the abdominal surgeries were performed by laparoscopy. Trials were classified as laparoscopic if ≥50% of the abdominal surgeries were performed by laparoscopy. Trials that did not report the percentage of laparoscopy were classified as “Not reported” and excluded from these subgroup analyses.

### Concept of preload variation

This subgroup concerns the GDHT-protocols that use targets based on differences in a haemodynamic variable at increasing stages of preload. In short, if increasing preload leads to an increase in the observed haemodynamic variable, then the patient is considered to be in need of increased intravascular volume (“fluid responsive”); if the increase in preload does not increase the observed haemodynamic variable, then other interventions than fluid therapy may be relevant. This is based on Starling’s curve stating that increasing preload leads to increased stroke volume until a certain plateau.

There are two concepts of preload variation that dominate the GDHT-literature. Other concepts exist but are rarely used for GDHT during surgical general anaesthesia. The first concept employs that mechanical ventilation increases intrathoracic pressure during inspiration which then decreases again during expiration – this creates a cyclic preload variation parallel to the respiratory cycle. A haemodynamic variable’s variation is assessed in relation to the different stages of the respiratory cycle, most often through automated software. Classic GDHT-examples of these variables include stroke volume variation (SVV), pulse pressure variation (PPV), and pleth variability index (PVI).

The second concept uses fluid challenges of typically 250-500 mL to increase preload, and a haemodynamic variable is measured before and after to yield a difference. Classic GDHT-examples of these variables include stroke volume difference (ΔSV) and cardiac index difference (∆CI).

Other concepts include passive leg raising or Trendelenburg-position which both increase preload by shifting blood from the lower extremity venous system towards the heart.

Trials assessing targets through preload variation of the respiratory cycle were classified as “respiratory cycle”. Trials assessing targets through fluid challenges were classified as “fluid challenges”. Trials using targets not based on preload variation (e.g., cardiac output/index (CO/CI), pulmonary artery occlusion pressure (PAOP)) or using mixed concepts of preload variation were excluded from these subgroup analyses. One trial used Trendelenburg-position and was also not included.

### Use of inotropes and/or vasopressor

This subgroup concerns GDHT-protocols that used either vasopressors or inotropes to reach one or more of the protocol’s targets. Only interventions different from the control were noted. Depending on this, trials were classified as “no inotrope or vasopressor” and “inotrope and/or vasopressor”.

### Intraoperative fluid amount differences between GDHT and standard care

Trials were classified as “similar fluid volumes” if the mean intraoperative fluid volume in the GDHT-arm was within ±500 ml of the mean intraoperative fluid volume in the standard care arm. In trials with “GDHT-protocol resulting in less intraoperative fluids”, patients in the GDHT-arm received >500 mL *less* fluid than patients in the standard care arm. In trials with “GDHT-protocol resulting in more intraoperative fluids”, patients in the GDHT-arm received >500 mL *more* fluid than patients in the standard care arm.

### Type of device

This subgroup concerns the technical equipment used to measure the haemodynamic target. Devices are often classified either as non-invasive (i.e., finger-cuffs, finger-clamps, and bioreactance), minimally-invasive (i.e., through a radial artery catheter or oesophageal Doppler), and invasive (i.e., pulmonary artery catheter).

Trials were grouped into three: 1) Non-invasive techniques (pleth variability index, finger pulse contour analysis, and bioreactance), 2) pulse contour analysis (through a radial artery catheter), and 3) oesophageal Doppler monitoring. Only the two oldest included trials (published in 1988 and 1997) used a pulmonary artery catheter reflecting the recent decades’ practice where it is rarely used for perioperative monitoring outside cardiac surgery. We therefore chose not to include these in the subgroup analyses.

### Type of fluid

This subgroup relates to the type of fluid used to reach the stroke volume-related target in the GDHT-protocol. Trials were classified as using either crystalloids (normal saline, Ringers’ lactate, Ringers’ acetate, Hartmann's solution etc.) or colloids (hydroxyethyl starch (HES), gelatin, or albumin). Trials using both crystalloids and colloids to reach the target, “fluids at the discretion of the treating anaesthesiologist”, or trials not reporting fluid types at all were excluded from these subgroup analyses.

## GRADE

The GRADE methodology was used to evaluate the overall certainty in the evidence for a given intervention and outcome. The following domains were considered.

### Risk of bias

Risk of bias was rated as “serious” for all interventions and outcomes since almost all of the individual trials were assessed as having an intermediate risk of bias.

### Inconsistency

Inconsistency was evaluated with the I^2^ statistic as well as visual inspection of the forest plot. As a guide, inconsistency was considered to the “serious” if the I^2^ statistic was > 40% but this also dependent on the distribution of the individual trials.

### Indirectness

Given that all the trials met our predefined criteria for inclusion and therefore represent the patient population of interest, indirectness was considered “not serious”. However, if the interest is in specific patient populations defined by patient characteristics or surgery type, it could be considered to assess indirectness as “serious”.

### Imprecision

In determining whether a given comparison and outcome should be downgraded for imprecision, we considered both the width of the confidence interval and the sample size (in that order). For binary outcomes, we mainly considered the effect on the relative scale (i.e., odds ratio) but also evaluated the effect on the absolute scale (i.e., risk difference). If the confidence interval was very wide (i.e., < 0.50 and > 2.00 for the odds ratio) and included both potential benefit and harm, we considered imprecision to be “very serious”. If the confidence was less wide and included potential benefit and no clear effect or harm, imprecision was considered “serious”. If the confidence interval only included clear benefit or harm or was narrow around no effect (i.e., within 0.80 to 1.20 for the odds ratio), we considered the sample size in relation to the optimal information size.^14^ For length of stay, a narrow confidence interval was considered within -0.25 to 0.25 days.

The optimal information size is equivalent to the required sample size of a single, adequately powered trial. It is challenging to determine a general optimal information size. However, considering an absolute risk reduction of 1%, the optimal information size would be 6206 for a control group outcome proportion of 2% and 39,494 for a control group outcome proportion of 11%. If a 5% absolute risk reduction were considered, the optimal information size would be 1164 for a control group outcome proportion of 10% and 2424 for 20%. These calculations are based on a chi-squared test, an alpha of 5%, and 90% power. Based on these considerations and previous suggestions^14^, we considered imprecision to be “serious” if the sample size was less than 2000 patients. If the sample size was less than 100 patients, we considered imprecision to be “very serious”. For consistency, we used the same sample sizes for length of stay.

### Others

In this domain, multiple aspects are considered including publication bias, the size of the effect, the direction of potential bias, and dose response gradients. The last two were not of relevance in the current review. Publication bias was assessed via funnel plots when feasible. We considered a large effect to be < 0.50 or > 2.00 on the odds ratio scale with reasonable narrow confidence intervals. For length of stay, we considered a large effect to be > 2 days.

# eTABLES

## eTable 1: Registered randomized controlled trials comparing goal directed therapies International Clinical Trials Registry Platform and ClinicalTrials.gov

| **Title** | **ID** | **Country** | **Year regi-stered** | **Estimated Completion** | **Intervention** | **Comparator(s)** | **Pa-tients** | **Status** |
| --- | --- | --- | --- | --- | --- | --- | --- | --- |
| Fluid therapy in thyroidectomy surgery | IRCT201410285140N14 | Iran | 2017 | 07/2017 | Fluid therapy based on pleth variability index | Conventional fluid therapy | 50 | Completed |
| A clinical trial of blood flow optimization for patients who have emergency bowel surgery | ISRCTN14729158 | United Kingdom | 2017 | 01/2022 | Treatment algorithm guided by cardiac output monitoring to determine dose and timing of intravenous fluid | Standard of care | 7646 | Recruiting |
| Optimization of perioperative cardiovascular management to improve surgical outcome ii | ISRCTN39653756 | Australia, Canada, Germany, Spain, Sweden, United Kingdom, United States of America | 2016 | 12/2022 | Cardiac output-guided fluid therapy with low dose inotrope infusion | Standard of care | 2502 | Recruiting |
| Effect of goal directed fluid therapy combined with norepinephrine infusion on postoperative outcome in elderly patients | ChiCTR-INR-17012594 | China | 2017 | 12/2018 | Goal directed fluid therapy combined with norepinephrine administration | Balanced solution infused at a constant rate of 5-7 mL/kg/h | 156 | Recruiting |
| The effect of intraoperative goal-directed fluid therapy on postoperative rehabilitation of elderly patients undergoing tumor resection surgery | ChiCTR-IOR-17011582 | China | 2017 | 07/2018 | Goal-directed fluid therapy | Standard of care | 180 | Not recruiting |
| The application of goal-directed fluid therapy in the fast-track anesthesia for gastrectomy | ChiCTR-INR-17010636 | China | 2017 | 03/2018 | Goal-directed fluid infusion | Standard of care | 100 | Recruiting |
| The study of goal-directed fluid therapy during traumatic brain injury | ChiCTR-INR-17012910 | China | 2017 | 12/2018 | Goal-directed fluid therapy | Standard of care | 180 | Recruiting |
| Effects of goal-directed fluid therapy based on pleth variability index on clinical outcomes of elderly patients undergoing gastrointestinal surgery: a prospective randomized and controlled trial. | ChiCTR-INR-17012220 | China | 2017 | 07/2021 | Goal-directed fluid therapy based on pleth variability index | Conventional fluid therapy | 220 | Recruiting |
| Effect of goal-directed fluid therapy on the rehabilitation of aged patients with laparoscopic colorectal surgery | ChiCTR-IOR-17013759 | China | 2017 | 11/2019 | Goal directed therapy with cardiac index ≥ 2.5 L/min/m2 | #1: Goal directed therapy with cardiac index ≥ 2.0 L/min/m2  #2: Standard of care | 90 | Recruiting |
| Comparison of three different liquid therapy in colorectal surgery | ChiCTR1800014777 | China | 2018 | 12/2018 | Goal-directed fluid therapy | #1: Fluid therapy and furosemide  #2: Restricted fluid therapy | 60 | Recruiting |
| Intraoperative use of non-invasive cardiac output monitoring versus standard fluid management in free perforator flap surgery: a randomized pilot trial | ChiCTR1900021018 | China | 2018 | 01/2019 | Floatrac system to guide intraoperative fluid management | Standard of care | 40 | Not recruiting |
| Continuous cardiac output and variation rate per wave monitoring were applied in the study of patients undergoing craniotomy with cardiovascular diseases | ChiCTR1900024660 | China | 2019 | 07/2020 | Adjustments to maintain cardiac output above 4 L/min | Conventional management | 200 | Not recruiting |
| Application of staged goal-directed fluid therapy in anesthesia for hepatectomy | ChiCTR2000029722 | China | 2020 | 07/2022 | Goal-directed fluid therapy based on stroke volume variation | Liquid management according to central venous pressure | 200 | Recruiting |
| Effects of intraoperative goal-directed fluid therapy on postoperative outcomes in elderly patients undergoing head and neck reconstructive surgery: a multicenter, randomized controlled trial | ChiCTR2000034084 | China | 2020 | 08/2020 | Goal-directed fluid therapy | Standard of care | 80 | Recruiting |
| Effects of goal-directed fluid therapy guided by SVV on intestinal barrier function in patients undergoing colon cancer surgery | ChiCTR2000039979 | China | 2020 | 08/2022 | Goal-directed fluid therapy | Standard of care | 60 | Recruiting |
| Effects of fluid optimization before induction of general anesthesia and intraoperative goal-directed therapy on the outcomes of elderly patients | ChiCTR2000040269 | China | 2020 | 12/2022 | Liquid optimization combined with goal-directed therapy | Routine anaesthesia management | 320 | Not recruiting |
| Effects of giving fluids as per goal on postoperative morbidity in patients undergoing open gastroenterology surgeries | CTRI/2016/12/007522 | India | 2016 | 12/2016 | Fluid therapy guided by stroke volume | Fluid therapy guided by central venous pressure | 100 | Completed |
| Comparison of two different methods of fluid management on function of kidney in patients undergoing surgery for kidney stones | CTRI/2018/12/016693 | India | 2018 | 04/2020 | Fluid therapy guided by stroke volume variation | Conventional fluid therapy | 40 | Not recruiting |
| A clinical study to see the effects of two different methods of fluid administration (fluid volume estimated by anesthetist compared with volume estimated by a machine) during cytoreductive surgery with hyperthermic intraperitoneal chemotherapy | CTRI/2020/08/027296 | India | 2020 | 02/2021 | Fluid therapy guided by stroke volume variation | Fluid therapy guided mean arterial pressure, central venous pressure, and urine output | 72 | Not recruiting |
| Comparing the amount of intravenous fluid required during surgery by using a variable derived from arterial blood pressure and conventional method by calculation of fluid and blood loss during surgery in brain surgeries done in supine position | CTRI/2019/04/018746 | India | 2019 | 11/2020 | Plasmalyte and normal saline provided alternatively to maintain normal pulse pressure variance or central venous pressure | Pulse pressure variance guided fluid management and fluid management based on conventional method using calculated losses along with central venous pressure | 80 | Not recruiting |
| Comparison of two parameters, one derived from an intra-arterial line and another derived from pulse oximeter to guide fluid therapy in patients undergoing elective surgery for brain tumors | CTRI/2019/08/020639 | India | 2019 | 02/2021 | Goal directed fluid therapy guided by stroke volume variation | Goal directed fluid therapy guided by plethysmography variability index | 98 | Not recruiting |
| A clinical trial to compare two methods of giving intravenous fluid therapy during the operation, conventional and goal directed, in major surgeries being carried out under general anesthesia. | CTRI/2020/02/023133 | India | 2020 | 02/2021 | Goal directed fluid therapy | Conventional fluid therapy | 60 | Not recruiting |
| The study to show factors affecting early recovery after surgery including fluid management | CTRI/2020/03/024318 | India | 2020 | 01/2021 | Goal directed fluid therapy based on stroke volume variation | Fluid therapy based on central venous pressure | 60 | Not recruiting |
| A scientific study to compare two dynamic parameters for fluid therapy in neurosurgery cases | CTRI/2020/12/029790 | India | 2020 | 12/2021 | Fluid therapy based on pulse pressure variation and pleth variability index | Fluid therapy based on pleth variability index | 80 | Not recruiting |
| Comparison of different types of fluid therapy in plastic reconstruction surgery | CTRI/2020/11/028876 | India | 2020 | 05/2021 | Goal directed fluid therapy | Standard of care | 100 | Not recruiting |
| A comparison of total fluid given by goal directed fluid therapy and conventional therapy in head injury patients undergoing surgery – a randomized controlled trial | CTRI/2021/02/031242 | India | 2021 | 02/2022 | Goal directed fluid therapy based on stroke volume variation, cardiac index, and mean arterial pressure | Fluid therapy at 2 mL/kg and boluses at the discretion of anaesthetist | 64 | Not Recruiting |
| Volume therapy controlled by pulse pressure variation in major abdominal surgery | DRKS00015710 | Germany | 2019 | NR | Crystalloid fluid at 4 mL/kg/h and a bolus of 500 mL as soon as the half-hourly measured pulse pressure variation is >10% | Crystalloid fluid at 4 mL/kg/h and a bolus of 500 mL as soon as the half-hourly measured pulse pressure variation is >30% | 720 | Recruiting |
| The effect of goal-directed therapy guided by stroke volume variation and cardiac index in non-severe surgical patients | NCT02841046 | China | 2016 | 06/2019 | Goal-directed fluid therapy based on cardiac index | Goal-directed fluid therapy based stroke volume variation | 50 | Completed |
| The effect of intraoperative goal directed restricted fluid therapy on extravascular lung water | NCT02845310 | Egypt | 2016 | 06/2017 | Restricted fluid management guided by stroke volume variation | Standard of care | 56 | Completed |
| Fluid oriented therapy for young ASA 1 patients | NCT03090399 | Italy | 2017 | 01/2017 | Fluids administered according to FloTrac parameters | Standard of care | 60 | Completed |
| Goal directed fluid management, PVI | NCT03956901 | Turkey | 2019 | 06/2020 | Fluid therapy based on pleth variation index | Liberal fluid therapy | 112 | Completed |
| Neurological effects of goal-directed fluid therapy in beach chair position shoulder surgery | NCT03963063 | Taiwan | 2019 | 09/2021 | Goal-directed fluid therapy | Standard of care | 100 | Recruiting |
| Restrictive vs goal directed fluid therapy during hepatobiliary surgery | NCT04092608 | Belgium | 2019 | 07/2020 | Goal-directed fluid therapy | Restrictive fluid therapy to maintain central venous pressure <7 mmHg | 40 | Completed |
| Brain-targeted goal-directed therapy in high-risk patients undergoing major elective surgery: the brain-promise study | NCT04266574 | Italy | 2020 | 12/2023 | Haemodynamic optimization based on near infrared spectroscopy | Standard of care | 200 | Recruiting |
| Recommendations of enhanced recovery interventions for patient's clinical team and collection of associated data | NCT04606264 | United States | 2020 | 04/2025 | Combinations of perioperative treatments | NA | 35485 | Not recruiting |
| Cardiac output optimization on postoperative complications in major hepatic surgery | NCT04655885 | France | 2020 | 07/2023 | Optimization of cardiac flow by base water-electrolyte supply of 1 mL/kg/h by Ringer Lactate and correction of VES <10% by administration of 250 mL Ringer Lactate | Increase basic hydro-electrolyte supply of 6 mL/kg/h by Ringer Lactate and 1: 1 blood loss compensation by crystalloids of the same nature | 186 | Not recruiting |
| Goal-directed therapy in neurosurgery. | NCT04754295 | Czech Republic | 2021 | 09/2021 | Administration of fluids and vasoactive drugs guided by non-invasive haemodynamic monitoring | Administration of fluids and vasoactive drugs guided by standard vital signs monitoring | 34 | Recruiting |
| Goal directed fluid therapy in major hepatic resection surgeries, a prospective randomized study | PACTR202012816670352 | Egypt | 2020 | 04/2021 | Haemodynamic and fluid management guided by systolic pressure variation | Fluid and haemodynamic management guided by central venous pressure | 90 | Recruiting |
| Goal directed fluid therapy during pancreaticoduodenectomy operation | PACTR201801002862138 | Egypt | 2017 | 12/2017 | Fluid therapy guided by stroke volume variation | Fluid therapy guided by central venous pressure | 40 | Recruiting |
| Monitoring of stroke volume variation and fluid responsiveness in laparotomy and pneumoperitoneum surgery | JPRN-UMIN000036240 | Japan | 2019 | 06/2022 | Fluid therapy when FloTrac stoke volume variation >10% | Fluid therapy when FloTrac stoke volume variation >15% | 120 | Recruiting |
| A comparison of return of gastrointestinal function between perioperative goal-directed therapy and traditional fluid therapy in major abdominal surgery patients: a prospective randomized controlled study | TCTR20170206008 | Thailand | 2017 | 08/2016 | Fluid and inotropic support based on arterial pulse contour analysis | Traditional fluid therapy | NR | Enrolling by invitation |
| A comparison of return of gastrointestinal function between oxygen content guided goal-directed therapy and standard fluid therapy in major abdominal surgery patients: a prospective randomized controlled study | TCTR20210204006 | Thailand | 2021 | 12/2020 | Oxygen content guided goal directed therapy | Standard fluid therapy | 70 | Completed |
| Clinical study for the effect of individualized hemodynamic management under multi-mode circulatory monitoring with optimal cardiac output on postoperative rehabilitation of elderly patients undergoing laparoscopic surgery | ChiCTR1900024010 | China | 2019 | NR | Haemodynamic management by using FloTrac/Vigileo alone | Multi-mode monitoring by using TTE, TEE, and FloTrac/Vigileo in haemodynamic management | 200 | Recruiting |
| Comparison of the effects of arterial blood pressure and cardiac output based hemodynamic management on cognitive function in elderly patients undergoing spinal surgery | ACTRN12619000471189 | Turkey | 2019 | NR | Haemodynamic management based on cardiac index | Haemodynamic management based on mean arterial pressure and systolic pressure | 60 | Recruiting |
| Clinical study for individualized hemodynamic monitoring in perioperative non-cardiac surgery of critical elderly patients | ChiCTR1900026143 | China | 2019 | NR | Haemodynamic management by using FloTrac/Vigileo alone | Multi-mode monitoring by using TTE, TEE, and FloTrac/Vigileo in haemodynamic management | 80 | Recruiting |
| Goal directed fluid therapy in spine surgery | IRCT20190719044274N1 | Iran | 2019 | NR | Fluid therapy based on haemodynamic monitoring | Standard of care | 40 | Completed |
| Management of intraoperative fluids in ambulatory surgery | NCT03193320 | Venezuela | 2017 | 12/2023 | Liberal fluid therapy | #1: Restrictive fluid therapy  #2: PVI guided fluid therapy | 243 | Not recruiting |
| Influence of a multi-parametric optimization strategy for general anesthesia on postoperative morbidity and mortality | NCT02668250 | France | 2016 | 02/2021 | Multi-parametric optimization strategy of anaesthesia including goal directed haemodynamic therapy and lung-protective ventilation | Standard of care | 2500 | Not recruiting |
| Individualized perioperative hemodynamic goal-directed therapy in major abdominal surgery (IPEGASUS-trial) | NCT03021525 |  | 2020 | 12/2022 | Goal directed haemodynamic therapy | Standard of care | 380 | Recruiting |
| Individualized hemodynamic optimization by indirect measurement of the respiratory quotient in major surgery (OPHIQUE) | NCT03852147 | France | 2019 | 01/2023 | Dynamic management based on respiratory quotient | Standard of care | 350 | Recruiting |
| Estimated oxygen extraction versus dynamic parameters for perioperative hemodynamic optimization | NCT04053595 | Italy | 2019 | 12/2021 | Haemodynamic optimization based on estimated oxygen extraction | Haemodynamic optimization based on dynamic parameters | 200 | Not recruiting |
| Blood gas assisted management of patients in major operations | CTRI/2018/07/014987 | India | 2018 | NR | Goal directed haemodynamic management | Standard of care | 80 | Closed for recruitment |
| A comparative study of blood flow stability in the body through intraoperative fluid administration by conventional versus a novel method in spine surgery | CTRI/2020/06/025723 | India | 2020 | NR | Fluid administration guided by stroke volume variation | Fluid administration guided by conventional parameters | 70 | Not recruiting |
| Patient management during major abdominal surgery (HEART-CORE) | NCT03113435 | Italy | 2017 | 12/2022 | Haemodynamic optimization based on oxygen consumption | #1: Haemodynamic optimization based the stroke volume  #2: Standard of care | 76 | Recruiting |

## eTable 2: Trials not included in meta-analyses with reason for non-inclusion and trial characteristics

| **First author, year of public-cation** | **Years of patient inclusion** | **Country** | **Type of surgery** | **Main inclusion criteria** | **Type of device to measure target** | **Device brand** | **GDHT targets** | | **Fluid therapy to reach target (bolus amount, type)** | **Vasoactive drugs to reach target** | **Comparator** |
| --- | --- | --- | --- | --- | --- | --- | --- | --- | --- | --- | --- |
| **REASON FOR EXCLUSION: Trials with supranormal or very restrictive targets** | | | | | | | | | | | |
| Shoe-maker, 1988^15^ | NR | United States | NR | High risk, general surgery or one of: massive haemorrhage, shock, sepsis, respiratory failure, acute abdominal catastrophe, acute renal failure, vascular disease, age > 70 | PAC | NR | CI > 4.5  DO_2_ > 600  VO_2_ > 170 | | NR | Noradrenaline, dopamine, dobutamine | Standard care, see included trials |
| Sand-ham, 2003^16^ | 1990 - 1999 | Canada | Multiple | Major abdominal, thoracic, vascular, or hip-fracture surgery, planned post-operative ICU-stay, age ≥ 60, ASA III-IV, | PAC | NR | DO_2_ 550-600  CI 3.5-4.5  MAP > 70  PCWP > 18 | | NR | NR | Standard care |
| Corbella, 2018^17^ | 2013-2015 | Canada | Urology | Deceased-donor kidney transplantation | ODM | “Cardio-Q-ODM”-monitor | ΔSV < 25 from “fluid optimized SV” at initiation | | 500 mL, crystalloid | None | Standard care |
| Nethan, 2018 ^18^ | NR | India | Abdo-minal | Open major abdominal surgery, estimated blood loss > 500 mL, duration of surgery expected > 1 hour | Pulse contour analysis and pleth curve analysis | BSM-9101 and Masimo pleth | PVI < 20  CI > 2.5 | | 200 mL, NR | Vasopressor (NR type), dopamine | Standard care |
| Wu, 2021^19^ | 2016-2017 | China | Urology | Laparoscopic or open partial nephrectomy, ASA I-III | Pulse contour analysis with lithium calibration | LiDCO rapid | SVV < 6  CI 3-4  MAP > 95  HR> 120% of baseline or > 100 | | 250 ml, crystalloid | Noradrenaline, dobutamine | Standard care |
| **REASON FOR EXCLUSION: Trials comparing two GDHT-protocol groups** | | | | | | | | | | | |
| Lobo, 2000^20^ | 1994-1996 | Brazil | Abdo-minal | ≥ 2 of the following: Age > 60, carcinoma-removal with expected surgery time ≥ 2h or AAA-repair, heart failure or former MI, ST-depression during stress test, or Q-waves on ECG or antiarrhythmic drug or COPD or liver cirrhosis | PAC | NR | DO_2_ > 600  PAOP > 16  Haematocrit < 32  MAP >70 (or > 80 if PAOP > 16) | 1000 ml, NaCl or Ringer or 500 Gelatine | | Dobutamine | GDHT:  Identical to intervention arm, but DO_2_ target > 520 |
| Abdul-lah, 2011^21^ | 2010-2011 | Egypt | Abdo-minal | Cirrhosis, open major abdominal surgery | Pleth contour analysis | Masimo set version | PVI < 13 | 250 ml, Ringers then HES | | None | GDHT:  Use of EDM:  CFT<350  ΔSV < 10 |
| Wang, 2012^22^ | NR | China | Gastro-intestinal | Elective gastrointestinal surgery, ASA I-II | Pulse contour analysis no calibration | Flotrac Vigileo, Edwards Lifesciences | SVV 11-13 | NR | | None | GDHT:  SVV maintained 5-7 |
| Choi, 2015^23^ | 2013-2015 | Korea | Hepatic | Elective living-donor liver lobectomy | Pulse contour analysis no calibration | Flotrac Vigileo, Edwards Lifesciences | SVV < 10 | Infusion rates changed, PlasmaLyte, or 20% albumin | | None | GDHT:  SVV > 10 infusion rate increased 7–10 mL/kg/h to maintain SVV < 10 |
| Feld-heiser, 2015^24^ | 2009-2010 | Germany | Hepatic | Elective liver resection, ASA I-III | Pulse contour analysis with lithium calibration | LiDCO  rapid | SVI < 10  SBP > 100  MAP > 65  SVI > 25% from baseline OR SVI drops < 40  CVP > 5 | 200 ml, colloid | | Noradrenaline | GDHT: Use of EDM  Protocol as intervention arm with same targets |
| Pavlovic, 2015^25^ | 2010-2013 | Switzer-land | Multiple | Severe sepsis or hypovolemia, emergency surgery, expected duration > 2 hours | Pulse contour analysis with thermodilution calibration | PiCCO-plus monitor | SVV < 13  ΔSV < 10  GEDVI 600-800  CI 2.5-3  ELWI < 10 | 250 ml, crystalloid, HES | | Phenylephrine, norepinephrine, epinephrine, dobutamine | GDHT:  PPV < 12 |
| Hassan, 2016^26^ | 2013-2015 | Egypt | Abdo-minal | Major abdominal surgery, expected duration > 120 minutes and blood loss > 1000 ml (e.g. radical cystectomy, radical prostatectomy, gastrectomy, pancreatectomy, spleenectomy), ASA I-II | Pulse contour analysis with thermodilution calibration | PICCO | SVV < 10  ΔSV < 10 | 200 ml, HES | | None | GDHT:  Using EDM:  FTC < 0.35  SV < 10 |
| Kong, 2016^27^ | 2015 | South Korea | Urology | Elective radical cystectomy, age 20 - 80 | Pulse contour analysis no calibration | Flotrac Vigileo, Edwards Lifesciences | SVV 10-20 | Infusion rate increased to 10 ml/kg  + bolus of 200 ml HES or 400 ml Hartmann’s solution | | None | GDHT:  SVV < 10. When SVV > 10% fluid rate was increased to 10 mL/kg/h + a bolus of either 200 mL crystalloid or 400 ml HES |
| Seo, 2016^28^ | 2014 | Korea | Hepatic | Donors scheduled for right hepatectomy for living donor liver transplantation | Pulse contour analysis, no calibration | EV1000, Edwards Lifesciences | SVV < 10 | Infusion rates changed, Mannitol | | None | GDHT:  SVV 10-20  Fluid: Mannitol |
| Warnaku-lasuriya, 2016^29^ | 2012-2013 | United Kingdom | Gastro-intestinal | Major colorectal surgery, low risk patients | ODM | NR | ΔSV < 10 | 200 ml, HES or Gelatine | | None | GDHT:  PVI < 14 |
| Wu, 2017^30^ | 2014-2017 | Taiwan | Neuro-surgery | Elective craniotomy for supratentorial brain tumour resection, age < 70 | Pulse contour analysis, no calibration | Flotrac Vigileo, Edwards Lifesciences | SVV < 18  Additional fluid boluses allowed if ΔCI decrease > 10% and SVV decrease > 5% | 250 ml, HES | | None | GDHT:  SVV < 10 |
| Bahl-mann, 2018^31^ | 2011-2014 | Sweden | Gastro-intestinal | Open abdominal surgery with expected duration ≥ 2 h, ASA I-III | Pulse contour analysis, no calibration | Masimo Radical 7 monitor | PVI < 10 | 3 ml/kg, Venofundin or Vololyte | | None | GDHT:  Using OD:  ΔSV < 10 |
| Coeck-elen-bergh, 2019^32^ | 2011-2013 | Belgium | Gastro-intestinal | Elective low-to-moderate risk abdominal surgery lasting > 1h, ASA I-III | Pleth contour analysis | Philips IntelliVue MP5-monitor | Two intervention groups:  PPV < 13 | 250 ml, Gelatine | | None | GDHT:  PVI > 15 |
| Lee, 2020^33^ | 2012-2015 | South Korea | Gastro-intestinal | Elective, open gastric or colorectal surgery, ASA I-II, weight 40-100 kg | Pulse contour analysis, no calibration | Flotrac Vigileo, Edwards Lifesciences | SVV < 13  CVP < 15 | 50 ml, HES | |  | GDHT:  SVV-target according to a 5 min baseline measurement in early surgery ("individual SVV"). Patient excluded if baseline-SVV > 13.  CVP < 15 |
| Wang, 2020^34^ | 2017-2018 | China | Neuro-surgery | Severe traumatic brain injury combined with ARDS, craniotomy and hematoma evacuation | Multiple tech. (State in "Notes") | See notes | SVV < 13 (or 15)  ΔCI > 15% | NR | | None | GDHT:  SVV < 13 |
| **REASON FOR EXCLUSION: Trials with targets correlating poorly with stroke volume** | | | | | | | | | | | |
| Venn, 2002^35^ | NR | UK | Ortho-paedic | Hip fracture surgery, age ≥ 65 | CVC | NR | ΔCVP < 3 | | 100 or 200 ml dependent on initial CVP, Gelatine | None | Standard care, see included trials |
| Donati, 2007^36^ | NR | Italy | Gastro-intestinal+vascular | Elective abdominal extensive surgery or abdominal aortic surgery | CVC | NR | O_2_ER < 27% | | 250-1000 ml, Colloids and red blood cells | Dobutamine | Standard care |
| Szak-many, 2005^37^ | 2002-2003 | Hungary | Gastro-intestinal+hepato-biliary  +pan-creatic | Elective oesophagectomy, total gastrectomy, pancreatectomy (Whipple’s), or liver resection, planned postoperative ICU | Pulse contour analysis with thermodilution calibration | PICCO | ITBVI 850-950 | | NR, HES or Gelatine | None | Standard care |
| Cohn, 2010^38^ | NR | United States | Gastro-intestinal | Elective open colorectal surgery, ASA I-III | Non-invasive oxygenation | InSpectra StO_2_ Tissue Oxygen Monitor | S_t_O2 > 75 or < 20% below baseline and either 1) SBP < 90 or > 20% below baseline, 2) HR > 100 or HR 20% above baseline, or 3) UO < 0.5 mL/kg/h for ≥ 2 hours and clinically judgment of hypovolemia | | 250 mL, Lactated Ringer’s | NR | Standard care |
| Jammer, 2010^39^ | 2007-2009 | Norway | Gastro-intestinal | Elective open colorectal or lower intestinal surgery | CVC | NR | S_cv_O2 >75 | | 3 mL/kg, HES | None | Standard care |
| Mikor, 2015^40^ | 2011-2013 | Hungary | Gastro-intestinal+vascular | Major abdominal/vascular surgery: oesophagectomy, gastrectomy, radical cystectomy, aorto-bifemoral bypass, abdominal aortic aneurysm repair | CVC | S_cv_O2-sensor (CeVOX-probe) | S_cv_O2 > 75 or sudden decrease < 3 | | 250 mL, HES | None | Standard care |
| Li, 2017^41^ | 2012-2014 | China | NR | Elderly patients undergoing general anaesthesia | ODM | FloTrac Vigileo, Edwards Lifesciences + Philips MP40-monitor | Inferior vena cava collapse index < 40% | | NR | None | Standard care |
| **REASON FOR EXCLUSION: Trials with no fluid intervention** | | | | | | | | | | | |
| Feng, 2010^42^ | 2006-2008 | China | Hepatic | Primary liver transplant without portal thrombosis | CVC | NR | CVP < 5 or > 40% lower than baseline  MAP > 60 | | No fluid therapy interventions | Norepinephrine, epinephrine | Standard care |
| ^a^In trials comparing two GDHT groups, we used authors’ definitions to define intervention and control.  Abbreviations and units in parenthesis (in alphabetical order): **CFT**: Corrected flow time (seconds), **CI:** Cardiac index (L/min/m^2^), **CVP:** Central venous pressure (mmHg), **DO_2_:** Oxygen delivery (mL/min/m^2^), **ELWI:** Extravascular lung water index (mL/kg), **GDHT:** Goal-directed therapy, **GEDWI:** Global end-diastolic volume index (ml/m^2^), **HES:** Hydroxyethyl starch, **HR:** Heart rate (min^-1^), **ITBVI:** Intra-thoracic blood volume index, **LiDCO:** Lithium Dilution Cardiac Output, **MAP:** Mean arterial pressure (mmHg), **NR:** Not reported, **O_2_ER:** Oxygen extraction ratio (VO_2_ / DO_2_),  **ODM:** Oesophageal Doppler monitoring, **PAC:** Pulmonary artery catheter, **PAOP:** Pulmonary artery occlusion pressure (mmHg), **PICCO:** Pulse Contour Cardiac Output, **PPV:** Pulse pressure variation (%), **PVI:** Pleth Variability Index (0-100), **SV:** Stroke volume (ml/s), **∆SV:** Delta stroke volume (%), **SVI:** Stroke volume index, **SVR:** Systemic vascular resistance (dyne-second/cm5), **SVV:** Stroke volume variation (%), **UO:** Urinary output, **VO_2_:** Maximal oxygen consumption (ml/min*m^2^) | | | | | | | | | | | |

## eTable 3: Characteristics of included trials

| **First author, country, year of publication (Years of patient inclusion)** | **Main inclusion criteria**  **(Type of surgery)** | **Main exclusion criteria** | **Age^a^ (years)** | **Sex (% male)** | **ASA (% for classes I-IV)** | **Patients random-ized^b^**  **(*n*)** | **Dura-tion of sur-gery (min)** |
| --- | --- | --- | --- | --- | --- | --- | --- |
| Shoemaker, USA, 1988  (NR on years of patient inclusion)^15^ | General surgery with ≥ 1 of the following high risk criteria: 1) Severe cardiorespiratory illness, 2) extensive surgery for carcinoma, 3) massive haemorrhage, 4) age >70, 5) shock, 6) sepsis w. haemodynamic instability, 7) respiratory failure, 8) acute abdominal catastrophe, 9) acute renal failure, 10) late-stage vascular disease  (NR on type of surgery) | No indication for invasive monitoring | 55 | 59 | NR | 91 | NR |
| Bender, USA, 1997  (1992-1995)^43^ | Elective infra-renal aortic reconstruction or lower limb re-vascularization  (Vascular) | Anticipated need for supraceliac or suprarenal cross clamp, MI within 3 months or unstable angina, congestive heart failure (i.e. such as peripheral oedema), CABG within 6 weeks, symptomatic aortic or mitral valve disease | 65 | 66 | NR | 104 | 321 |
| Sinclair, UK, 1997  (NR on years of patient inclusion)^44^ | Acute fractured neck of femur, age > 55  (Orthopaedic) | Fracture secondary to neoplasm, fractures occurring in-hospital, fracture through the site of a previous surgical correction or associated with instability of a previous prosthesis, planned regional anaesthesia, refusal of/impossible to get consent | 75 | NR | NR | 40 | 72 |
| Conway, UK, 2002  (NR on years of patient inclusion)^45^ | Major colorectal bowel resection  (Gastrointestinal) | Emergency, intrathoracic or oesophageal surgery, oesophageal disease | 67 | NR | NR | 57 | 137 |
| Gan, USA, 2002  (NR on years of patient inclusion)^46^ | Major general, urologic, or gynaecologic surgery, anticipated blood loss > 500 mL, ASA I-III  (Abdominal^c^) | Preoperative bowel obstruction, coagulopathy, renal and hepatic dysfunction, congestive heart failure, oesophageal pathology, gastric or oesophageal surgery, antiemetic medication within 3 days of surgery. | 56 | 57 | I: 11  II: 68  III: 21 IV: 0 | 100 | NR |
| Venn, UK, 2002  (NR on years of patient inclusion)^35^ | Acute hip fracture surgery, age ≥ 65  (Orthopaedic) | Oesophageal pathology, central venous cannula already in situ, pathological fracture of femur | 84 | 17 | I: 0  II: 0  III: NR IV: NR | 90 | NR |
| Wakeling, UK, 2005  (2001-2003)^47^ | Elective large bowel surgery  (Gastrointestinal) | Hepatic pathology, perforated viscus, oesophageal pathology, coagulopathy | 69 | 56 | I: 0  II: 100 III: 0  IV: 0 | 128 | NR |
| Noblett, UK, 2006  (NR on years of patient inclusion)^48^ | Elective colorectal surgery  (Gastrointestinal) | Severe oesophageal disease, recent oesophageal or upper airway surgery, use of systemic steroids, moderate to severe aortic valve disease, bleeding | 65 | NR | NR | 108 | 158 |
| Lopes, Brazil, 2007  (2005-2006)^49^ | High-risk surgery requiring postoperative ICU-care  (Abdominal^c^) | Arrhythmia, BMI > 40, thoracotomy, neurosurgery, acute surgery | 63 | 70 | I: 0  II: 18  III: 52 IV: 30 | 33 | 228 |
| Buettner, Germany, 2008 (2002-2003)^8^ | Major elective abdominal or gynaecological surgery with bowel resection and anticipated blood loss > 500 mL  (Abdominal^c^) | Age < 18, emergency surgery, coagulopathy, renal and hepatic dysfunction, hyperthyroidism, cardiac rhythm other than sinus | 64 | 45 | NR | 80 | 300 |
| Harten, UK, 2008  (2003-2005)^50^ | Emergency abdominal surgery, age > 50, only inclusion 8 a.m. - 8 p.m.  (Gastrointestinal) | Trauma, vascular surgery, duration of surgery expected < 90 min, lithium drug therapy | 65 | 79 | NR | 30 | 110 |
| Senagore, USA, 2009  (2008-2009)^51^ | Laparoscopic hemicolectomy, ASA I-III  (Gastrointestinal) | Stoma formation or closure, total colectomy or procto-colectomy, major surgery within the past month, pregnancy, psychiatric patients, prisoners, bowel obstruction, coagulopathy, renal or hepatic dysfunction, congestive heart failure, unstable angina, NYHA ≥ 2, oesophageal pathology, antiemetic medication | NR | NR | NR | 64 | NR |
| Benes, Czech Republic, 2010  (2007-2009)^52^ | Major open abdominal surgery with duration > 120 min OR presumed blood loss > 1,000 mL AND at least one of: 1) ischemic heart disease, 2) severe heart dysfunction 3) COPD, 4) age > 70, 5) ≥ASA III  (Abdominal^c^) | Irregular heart rhythm, weight < 55 or > 140 | 67 | 81 | I: 0  II: 21  III: 64 IV: 15 | 120 | 170 |
| Forget, Belgium, 2010 (2008)^53^ | Oesophagectomy, gastric resection/suture, hepatectomy, pancreatectomy, or intestinal colorectal surgeries  (Abdominal^c^) | Arrhythmia, cardiac ejection fraction 30%, lung pathology prohibiting mechanical ventilation with tidal volumes larger than 6 mL/kg, and kidney dialysis. | 60 | 61 | I: 0  II: 54  III: 46 IV: 0 | 86 | 298 |
| Mayer, Germany, 2010  (2008-2009)^54^ | Open, elective, major abdominal surgery, ASA ≥ 3, ≥ 2 Lee Cardiac Risk Index risk factors  (Abdominal^c^) | Severe aortic regurgitation, permanent arrhythmia | 73 | 70 | I: 0  II: 0  III: 100 IV: 0 | 60 | 289 |
| Van der Linden, Belgium, 2010  (2006-2008)^55^ | Peripheral arterial bypass grafting, ASA II-III  (Vascular) | Atrial fibrillation, aortic and mitral valve disease, treatment with oral anti-diabetic drugs | 68 | 70 | NR | 60 | 210 |
| Pillai, UK, 2011  (2006-2009)^56^ | Radical cystectomy, ASA I-III  (Abdominal^c^, urology) | Oesophageal disease/stricture, recent oesophageal or laryngeal surgery, moderate to severe aortic valve disease, patient choice | 67 | 30 | NR | 66 | 325 |
| Brandstrup, Denmark, 2012  (2008-2009)^57^ | Colorectal resection,  ASA I-III  (Gastrointestinal) | Disseminated cancer, alcoholic drinks >5 per day, pregnant, contraindications for hydroxylethyl starch | 68 | 57 | I: 31  II: 53  III: 16 IV: 0 | 151 | NR |
| Challand, UK, 2012  (2009-2010)^58^ | Major colorectal surgery, aerobic fit to complete cardiopulmonary exercise testing  (Gastrointestinal) | Anaerobic threshold < 8 ml O2/kg/min or not detectable | 66 | 57 | I: 12  II: 58  III: NR IV: NR | 179 | NR |
| Zhang, China, 2012  (NR on years of patient inclusion)^59^ | Gastric or colon cancer-resection, anticipated blood loss < 500 mL, age 18-64  (Gastrointestinal) | BMI > 30, significant arrhythmia, cardiopulmonary dysfunction, peripheral arterial occlusive disease, renal or liver disease, pregnancy, coagulopathy | 54 | 70 | I: 62  II: 38  III: 0  IV: 0 | 60 | 185 |
| Bisgaard, Denmark, 2013  (2008-2010)^60^ | Open, elective abdominal aortic surgery  (Abdominal^c^, vascular) | End-stage renal failure, lithium therapy, weight < 40 | 68 | 70 | NR | 70 | 217 |
| Bundgaard-Nielsen, Denmark, 2013  (2008-2009)^61^ | Open radical prostatectomy, ASA I-III  (Abdominal^c^, vascular) | Sedative premedication, psychiatric disease, alcohol abuse, kidney disease, coagulation impairment, opioid consumption, orthostatic hypotension, use of β-blockers, need for intraoperative infusion of vasopressor or inotropic agents other than ephedrine, contraindication to oesophageal Doppler use | 64 | 100 | I: 62  II: 38  III: 0  IV: 0 | 44 | 157 |
| El Sharkawy, Egypt, 2013  (2010-2011)^62^ | Major liver resection (≥3 segments) with no Pringle manoeuver, hepatic cirrhosis (CHILD ≥A), age 18-60, ASA I-II  (Abdominal^c^, hepatic) | Contraindication for oesophageal Doppler insertion, arrhythmia (frequent ectopic beats), history of bleeding tendency | 49 | 51 | I: 33  II: 67 III: 0  IV: 0 | 59 | 294 |
| McKenny, Ireland, 2013  (NR on years of patient inclusion)^63^ | Major, open gynecology surgery (malignancy of uterus and/or adnexae, with or without omentectomy, lymph node dissection) or bowel resection, ASAI-III  (Abdominal^c^) | EF < 30%, oesophageal pathology or recent upper gastrointestinal surgery, known hypersensitivity to hydroxyethyl starch, renal disease, hepatic disease | 58 | 0 | NR | 102 | 150 |
| Ramsingh, USA, 2013  (NR on years of patient inclusion)^64^ | Major open abdominal non-vascular surgery  (Abdominal^c^) | Coagulopathy, cerebrovascular disease, renal or hepatic dysfunction, congestive heart failure, ischemic heart disease, arrhythmia, lung disease | 59 | 29 | NR | 46 | 282 |
| Salzwedel, multiple countries, 2013  (2011-2012)^65^ | Elective open abdominal surgery, expected duration > 2 hours or estimated blood loss > 20%, ASA II-IV, indication for arterial line and central venous catheter  (Abdominal^c^) | Planned ICU stay, pregnant or lactating, arrhythmia | 64 | 60 | I: 0  II: 59  III: 41 IV: 0 | 180 | 229 |
| Scheeren, multiple countries, 2013  (2008-2009)^66^ | Surgery requiring postoperative ICU-care, ASA III-IV, indication for arterial line and central venous catheter, thoracic epidural anaesthesia  (Abdominal^c^) | Arrhythmia, BMI > 40, surgery with an open thorax, neurosurgery, hepatic surgery, emergency surgery | 70 | 67 | I: 0  II: 0  III: 96 IV: 4 | 64 | 277 |
| Srinivasa, New Zealand, 2013  (2009-2011)^67^ | Elective colectomy, ASA I-III  (Gastrointestinal) | Severe oesophageal disease, recent oesophageal or upper airway surgery, moderate or severe aortic valve disease, bleeding diathesis, cortico- or mineralocorticoids, cognitive impairment, rectal tumour, stoma formation | 71 | 55 | I: 14  II: 47  III: 39 IV: 0 | 85 | NR |
| Zakhaleva, USA, 2013  (2010-2011)^68^ | Open or laparoscopic elective bowel resection with primary anastomosis  (Gastrointestinal) | Recent oesophageal or upper airway surgery, moderate or severe aortic valve disease, congestive heart failure | 57 | 57 | I: 0  II: 19  III: 81 IV: 0 | 91 | 220 |
| Zheng, China, 2013  (1999-2011)^69^ | Moderate to high-risk surgery, prior coronary angiography, coronary heart disease, ASA I-II, NYHA II-III, BMI 18-24, anticipated blood loss < 600 mL, age 60-80, ASA I-III  (Gastrointestinal) | Fluid therapy within 48 hours, congenital heart disease, cardiomyopathy, rheumatic heart disease, pulmonary heart disease, use of vasoactive drugs within 3 months, administration of diuretics, acid-base or electrolyte imbalance, difficulty placing central line, blood-borne infectious disease, undergone surgery twice after admission, dehydration | 68 | 50 | I: 0  II: 40  III: 60 IV: 0 | 65 | 158 |
| Pearse, UK, 2014  (NR on years of patient inclusion)^70^ | Major surgery of the gastrointestinal tract, expected duration > 90 min, age ≥ 50, at least one of: 1) Age ≥ 65, 2) ischemic heart disease, 3) EF < 30%, 4) moderate or severe valve disease, 5) COPD, 6) poor lung function, 7) lung disease, 8) low anaerobic threshold, 9) heavy smoker, 10) renal impairment, 11) diabetes, 12) emergency surgery  (Gastrointestinal) | Pregnancy, acute pulmonary oedema, MI, palliative surgery | 72 | 64 | I: 6  II: 51  III: 41 IV: 2 | 734 | 265 |
| Peng, China, 2014  (NR on years of patient inclusion)^71^ | Major elective orthopaedic surgery, anticipated blood loss > 800 ml  (Orthopaedic) | BMI > 40 or < 15, coagulopathy, arrhythmia or cardiopulmonary dysfunction, renal or liver disease | 54 | 43 | I: 42  II: 54  III: 4  IV: 0 | 80 | 169 |
| Pestaña, Spain and Israel, 2014  (2011-2012)^72^ | Open elective colorectal surgery, gastrectomy or small bowel resection, requiring ICU-admission  (Gastrointestinal) | Intraabdominal infection, life expectancy < 60 days, disseminated malignancy | 74 | 56 | I: 3  II: 46  III: 50 IV: 1 | 170 | 182 |
| Phan, Australia, 2014  (2012-2013)^73^ | Major elective colorectal surgery, ASA I-III  (Gastrointestinal) | Pregnancy, renal dysfunction, hepatic dysfunction, NYHA III-IV, oesophageal pathology | 64 | 61 | NR | 100 | 233 |
| Shillcutt, USA, 2014  (2010-2011)^74^ | Major elective non-cardiac surgeries, Age > 65 or risk factors for left ventricular dysfunction, echocardiographic evidence of left-ventricular dysfunction, ASA I-IV  (Mixed surgical specialties) | Expected hospital stay < 24 hours, inability to undergo trans-oesophageal echocardiography, ASA V, intracranial pressure, shock, sepsis | 70 | 36 | NR | 28 | 66 |
| Benes, Czech Republic, 2015  (2012-2013)^75^ | Total hip or knee replacement, age > 18  (Orthopaedic) | Finger oedema, arrhythmia, need for direct and continuous blood pressure monitoring or advanced haemodynamic monitoring | 67 | 39 | I: 16  II: 64  III: 20 IV: 0 | 80 | 103 |
| Colantonio, Italy, 2015  (2010-2012)^76^ | Surgery for peritoneal carcinomatosis with peritonectomy and  hyperthermic intraperitoneal chemotherapy (HIPEC), ASA II–III  (Gastrointestinal) | Haemodynamically significant aortic regurgitation and heart rhythm disorders | 56 | 66 | I: 0  II: 92  III: 8  IV: 0 | 80 | 570 |
| Correa-Gallego, USA, 2015  (2012-2014)^77^ | Elective open liver resection  (Abdominal^c^, hepatic) | Active coronary/cerebrovascular/ congestive heart disease, atrial fibrillation or flutter, oxygen saturation <90%, renal dysfunction, severe hepatic dysfunction or portal hypertension, pregnancy,  BMI > 45 or < 17 | 57 | 37 | NR | 135 | 258 |
| Funk, Canada, 2015  (NR on years of patient inclusion)^78^ | Elective open abdominal aortic aneurism (AAA)-repair  (Abdominal^c^, vascular) | Age > 80, weight > 120 kg, aortic insufficiency, renal dysfunction, congestive heart failure, atrial fibrillation | 68 | 68 | NR | 40 | 219 |
| Jammer, Norway and Finland, 2015  (2012-2013)^79^ | Open major abdominal surgery, ASA III-IV  (Abdominal^c^) | Liver or oesophageal surgery, atrial fibrillation, severe aortic or mitral stenosis | NR | NR | NR | 30 | NR |
| Kumar, India, 2015  (NR on years of patient inclusion)^80^ | High-risk gastrointestinal or vascular surgery, ASA II-III  (Abdominal^c^) | Renal dysfunction, liver dysfunction, EF ≤ 35%, hepatic resection, condition that contraindicated fluid resuscitation | NR | NR | NR | 40 | NR |
| Lai, UK, 2015  (2011-2013)^81^ | Elective rectal resection or cystectomy with ileac conduit  (Abdominal^c^) | Recent MI, unstable angina, uncontrolled arrhythmia, syncope, active endocarditis, acute myocarditis or pericarditis, symptomatic severe aortic stenosis, uncontrolled heart failure, acute pulmonary embolus or pulmonary infarction, DVT, suspected dissecting aneurysm, uncontrolled asthma, pulmonary oedema, oxygen saturation <85% at rest, respiratory failure, or acute non-cardiopulmonary disorder that might affect exercise performance or be aggravated by exercise | 63 | 61 | I: 13  II: 70  III: NR IV: NR | 221 | 198 |
| Broch, Germany, 2016  (2014-2015)^82^ | Major open abdominal surgery, estimated duration ≥120 min, anticipated blood loss ≥1000 mL, ASA II-III  (Abdominal^c^) | Arrhythmia, advanced peripheral artery occlusive disease, arteriovenous shunts concerning upper extremities | 66 | 73 | I: 0  II: 71 III: 29 IV: 0 | 86 | 228 |
| Hand, USA, 2016  (2012-2014)^83^ | Primary free tissue transfer reconstruction  (Plastic) | Cognitive limitation, NYHA > III or EF < 30%, severe pulmonary disease, weight < 55 or > 160, arrhythmia | 58 | 75 | I: 0  II: 18  III: 80  IV: 2 | 94 | 657 |
| Kumar, India, 2016  (NR on years of patient inclusion)^84^ | Major gastrointestinal surgery, ASA I-II  (Gastrointestinal) | Arrhythmia, BMI > 40, combined abdominal and open thoracic surgery | 56 | 62 | I: 53  II: 47  III: 0  IV: 0 | 60 | 468 |
| Schmid, Germany, 2016  (2010-2012)^85^ | Major non-cardiac surgery, expected duration > 3 hours, planned ICU stay > 3 days, ASA I-III  (Gastrointestinal) | Dialysis | 66 | 77 | NR | 193 | NR |
| Elgendy, Egypt, 2017  (2014-2016)^86^ | Major abdominal surgery, duration > 120 min or blood loss > 20%, ASA II-III, ≥ 1 Lee Cardiac Risk Index risk factor  (Abdominal^c^) | NR | 58 | 59 | I: 0  II: 43  III: 57 IV: 0 | 86 | 230 |
| Gómez-Izquierdo, Canada, 2017  (2013-2015)^87^ | Elective laparoscopic colorectal resection  (Gastrointestinal) | Previous oesophageal or gastric surgery, oesophageal varices or cancer, aortic coarctation, chronic atrial fibrillation, severe aortic stenosis, preoperative bowel obstruction, coagulopathies, contraindications to epidural analgesia, chronic opioid use | 62 | 55 | I: 11  II: 63  III: 25  IV: 1 | 135 | 186 |
| Liang, China, 2017  (2014-2015)^88^ | Transurethral resection of prostate due to benign prostatic hyperplasia, ASA II-III, age 60-80, hypertension, | Abnormal liver function, abnormal kidney function, BMI ≥30, perioperative haematocrit ≤ 0.30, secondary hypertension, prostate cancer, severe cardiopulmonary disease, arrhythmia | 72 | 100 | I: 0  II: 72 III: 28 IV: 0 | 60 | 120 |
| Luo, China, 2017  (NR on years of patient inclusion)^89^ | Elective craniotomy (tumour resection, abscess, or aneurysm) with expected duration > 2 hours, ASA III-IV  (Neurosurgery, intracranial) | Weight < 40 or > 100, arrhythmia | 62 | 43 | I: 0  II: 0  III: 97 IV: 3 | 150 | 275 |
| Reisinger, Netherlands, 2017  (2010-2013)^90^ | Elective colorectal cancer surgery with primary anastomosis  (Gastrointestinal) | Use of steroids, oesophageal varices or other oesophageal disease, aortic valve disease | 68 | 71 | I: 21  II: 66  III: 14 IV: 0 | 58 | 231 |
| Stens, Netherlands, 2017  (2012-2013)^91^ | Elective moderate-risk abdominal surgery | Cardiac arrhythmia, planned ICU-admission, BMI < 20 or > 40, cardiac decompensation, aortic valve disease, EF < 30%, aortic valve stenosis, pulmonary arterial pressure > 30 mmHg, tricuspid annular plane systolic excursion < 18 mm | 63 | 55 | NR | 244 | 142 |
| Weinberg, Australia, 2017  (2013-2015)^92^ | Elective pancreatico-duodenectomy, ASA I-IV  (Gastrointestinal) | Pregnancy, coagulopathy, renal impairment, chronic liver disease, distal or central or total pancreatectomy or pancreatic enucleation | 65 | 56 | NR | 52 | 492 |
| Wu, China, 2017  (NR on years of patient inclusion)^93^ | Supratentorial neoplasms (meningioma, glioma or metastatic tumour), ASA I-II  (Neurosurgery) | Severe cardiovascular or respiratory disease, severe aortic stenosis or regurgitation, permanent arrhythmia, severe peripheral vascular disease, or obesity (body mass index ≥35), reoperation in 24 h | 50 | 46 | I: 35  II: 65 III: 0  IV: 0 | 66 | 193 |
| Calvo-Vecino, Spain, 2018  (2011-2014)^7^ | Elective major abdominal, urological, gynecological or orthopedic surgery, expected duration > 2 hours, ASA I-III  (Abdominal^c^ + Orthopedic) | Contraindications for oesophageal Doppler, aortic pathology | 65 | 62 | I: 14  II: 60  III: 26 IV: 0 | 428 | 191 |
| Kaufmann, Germany, 2018  (2015-2016)^94^ | Cemented hip arthroplasty  (Orthopaedic) | BMI > 50, oesophageal- or gastric pathologies, pregnancy | 79 | 39 | I: 0  II: 30  III: 57 IV: 14 | 90 | 122 |
| Kim, South Korea, 2018  (2014-2016)^95^ | Elective free flap reconstruction for head and neck cancer  (Plastic) | Valvular heart disease, congestive heart failure, cardiac arrhythmia, renal failure, liver cirrhosis, liver failure, coagulation abnormalities, allergic reaction to hydroxyethyl starch solution | 56 | 78 | I: 21  II: 50  III: 29 IV: 0 | 62 | 647 |
| Yin, China, 2018  (2014-2015)^96^ | Elective colorectal surgery, age 65-90, BMI 18 – 28, ASA II-III  (Gastrointestinal) | Liver dysfunction, renal dysfunction | 69 | 47 | I: 0  II: 46  III: 54 IV: 0 | 50 | 201 |
| Zhang, China, 2018  (NR on years of patient inclusion)^97^ | Surgery for spinal stenosis, age > 60, ASA I-II, BMI < 30, haematocrit > 0.35,  (Neurosurgery, spinal) | Chronic disease, history of mental illness, neurological disease, receiving drugs that may affect cognitive function, arrhythmia, liver or kidney dysfunction, severe cardiopulmonary disease, low Montreal Cognitive Assessment Score | 65 | 67 | I: 53  II: 47  III: 0  IV: 0 | 83 | 121 |
| Zhao, China, 2018  (2016)^98^ | Surgery for gastrointestinal cancer, age > 60, ASA II-III, living above 2000 m altitude  (Gastrointestinal) | Severe aortic regurgitation, rapid arrhythmia, requiring intraaortic counter-pulsation | 69 | 50 | I: 0  II: 68  III: 32 IV: 0 | 88 | 221 |
| Cesur, Turkey, 2019  (NR on years of patient inclusion)^99^ | Elective open colorectal tumour surgery, ASA I-II  (Gastrointestinal) | Arrhythmia, peripheral artery disease, EF < 30%, pulmonary pathology, liver or renal dysfunction | 61 | 41 | I: 59  II: 41  III: 0  IV: 0 | 70 | 145 |
| Davies, UK, 2019  (2015-2017)^100^ | Urgent or acute repair of a proximal femoral fracture, high-risk patients, age > 50, ASA I-IV  (Orthopaedic) | Multiple injuries requiring operative management | 87 | 42 | I: 1  II: 14  III: 66 IV: 19 | 241 | 67 |
| Godai, Japan, 2019  (2017-2018)^101^ | Elective open gynaecological surgery requiring arterial line, ASA I-III  (Abdominal^c^, gynaecology) | Uncompensated heart failure, stroke, arrhythmia, severe liver or renal dysfunction | 46 | 0 | I: 30  II: 71  III: 0  IV: 0 | 40 | 239 |
| Hasanin, Egypt, 2019  (2017-2018)^102^ | Supratentorial mass excision  (Neurosurgery, intracranial) | BMI > 40, arrhythmia, pulmonary hypertension, impaired cardiac contractility, liver or kidney dysfunction | 40 | 46 | I: 64  II: 36  III: 0  IV: 0 | 61 | 324 |
| Liu, China, 2019  (2017-2018)^103^ | Laparoscopic resection of colorectal cancer, age 65-80, ASA I-II  (Gastrointestinal) | Uncompensated cardiac or respiratory disease, neurological disease, peripheral vascular disease, coagulopathy, anaemia, renal impairment, previous major operation | 70 | 62 | I: 15  II: 85  III: 0  IV: 0 | 74 | 181 |
| Sujatha, India, 2019  (2014-2018)^104^ | Elective open major bowel surgery requiring invasive arterial pressure monitoring, age 20-70, ASA I-II  (Gastrointestinal) | Heart failure or renal failure, simple colostomy or jejunostomy | 53 | 65 | I: 51  II: 49 III: 0  IV: 0 | 306 | 262 |
| Szturz, Czech Republic, 2019  (2014-2016)^105^ | Elective open gastrointestinal surgery, expected duration > 2 hours or expected blood loss > 15%, ASA II-III  (Gastrointestinal) | Pregnancy, sepsis, oesophageal pathology or prior oesophageal surgery | 65 | 60 | I: 0  II: 50  III: 45 IV: 5 | 150 | 190 |
| Weinberg, Australia, 2019  (2013-2016)^106^ | Elective major liver resection, ASA I-III  (Abdominal^c^, hepatic) | Coagulopathy, thrombocytopenia, renal impairment, hepatic insufficiency, EF < 40%, atrial fibrillation, moderate or severe tricuspid regurgitation, impairment of right ventricular function. | 63 | 64 | I: NR  II: NR III: 78 IV: 0 | 50 | NR |
| Arslan-Carlon, USA, 2020  (2014-2018)^107^ | Open radical cystectomy  (Abdominal^c^, urology) | Atrial fibrillation, BMI > 45 | 69 | 78 | NR | 320 | 314 |
| De Cassai, Italy, 2020  (2018-2019)^108^ | Deceased-donor kidney transplantation  (Abdominal^c^, urology) | Double-kidney transplantation, multi-organ transplantation, heart disease, arrhythmia | 53 | 75 | I: 0  II: 0  III: 100 IV: 0 | 40 | 223 |
| Fischer, France, 2020  (2015-2017)^109^ | Elective hip or knee arthroplasty, sinus rhythm  (Orthopaedic) | Pregnancy, arrhythmia, sepsis, chronic kidney disease with dialysis, dark-coloured skin | 66 | 37 | I: 16  II: 69  III: 16 IV: 0 | 447 | NR |
| Iwasaki, Japan, 2020  (2015-2016)^110^ | Elective major hepatobiliary-pancreatic surgery with planned postoperative ICU-admission  (Abdominal^c^) | Only cholecystectomy, atrial fibrillation, anaemia or hypoalbuminemia, dialysis, massive bleeding or anaphylaxis or cardiac arrest anticipated | 69 | 23 | NR | 60 | 312 |
| Nicklas, Germany, 2020  (2016-2017)^111^ | Elective major abdominal surgery (general, urological, gynaecological, or vascular) with duration > 90 min or expected blood loss >1000 ml and one high risk criteria (see trial)  (Abdominal^c^) | Pregnancy, palliative surgery, participating in other interventional trials | 63 | 61 | I: 0  II: 13  III: 73 IV: 13 | 188 | 246 |
| Schneck, Germany, 2020  (2017-2018)^112^ | Total hip arthroplasty, ASA I-III  (Orthopaedic) | Pregnancy or nursing, participation in another interventional study, contraindications for invasive blood pressure monitoring, need for dialysis, coagulopathy | 63 | 51 | I: 4  II: 78  III: 18 IV: 0 | 50 | 81 |
| Diaper, Switzerland, 2021  (2010-2018)^113^ | Elective open major abdominal, urological, or vascular surgery, duration > 2 hours  (Abdominal^c^) | End-stage organ failure (with Child-Pugh class ≥C or model for end-stage liver disease score >22, on haemodialysis or hemofiltration, with forced expiratory lung volume in 1 second <30% of predicted values), psychiatric disorders | 64 | 64 | NR | 401 | 306 |
| Trials listed according to publication year.  ^a^Age: Mean or median, as reported by the trial.  ^b^Patients randomized includes those who were excluded from analyses due to protocol violations, drop-out, loss-to-follow up etc.  ^c^Abdominal as defined in the subgroup abdominal vs. non-abdominal, meaning that it was within the abdominal cavity (including the retroperitoneal space) but could be multiple surgical specialties  Abbreviations in alphabetical order: **ASA:** American Society of Anesthesiologists classification, **BMI:** Body mass index, **COPD:** Chronic obstructive pulmonary disease, **ICU:** Intensive care unit, **NR:** Not reported, **NYHA:** New York Heart Association Classification, **UK:** United Kingdom, **USA:** United States of America | | | | | | | |

## eTable 4: Reported outcomes in trials

| **Study** | **Fluid volume difference** | **Mortality^a^** | **Hospital length of stay** | **Combined Pulmonary^b^** | **Pneumonia** | **Pulmonary Oedema** | **Acute Lung Injury** | **ARDS^c^** | **Pulmonary embolism** | **Combined cardiovascular^d^** | **Myocardial infarction** | **Arrhythmia** | **Acute Kidney Injury** | **Surgical Site infection** | **Combined Abdominal^e^** | **Paralytic ileus** | **Anastomotic leakage** | **Delirium** |
| --- | --- | --- | --- | --- | --- | --- | --- | --- | --- | --- | --- | --- | --- | --- | --- | --- | --- | --- |
| Shoe-maker, 1988^15^ |  | X | X | (X) |  | (X) |  |  | (X) |  | (X) |  | (X) | X |  |  |  |  |
| Bender, 1997^43^ | (only cryst-alloid reported) | X | X |  |  | (X) |  |  |  |  | X | (X) | (X) | (X) |  |  |  |  |
| Sinclair, 1997^44^ | X | X | X |  |  |  |  |  |  |  |  |  |  |  |  |  |  |  |
| Conway, 2002^45^ |  | X | X |  |  |  |  |  |  |  |  | (X) |  |  |  |  |  |  |
| Gan, 2002^46^ | X |  | X |  | X |  |  |  |  | (X) |  |  | (X) | X |  |  |  |  |
| Venn, 2002^35^ | X | X | (X) |  | X |  |  |  | X |  | (X) | (X) | (X) | X |  |  |  |  |
| Wakeling, 2005^47^ | (X) | (X) | X | (X) |  |  |  |  |  | (X) |  |  |  |  | (X) |  | X |  |
| Noblett, 2006^48^ | X | X | X |  | X |  |  |  |  |  |  |  |  |  |  | (X) | X |  |
| Lopes, 2007^49^ | (X) | X | X |  | X | (X) | (X) |  | X |  |  | (X) | (X) |  |  | X | X |  |
| Buettner, 2008^8^ | X | X | X |  |  |  |  |  |  |  |  | (X) |  |  |  |  |  |  |
| Harten, 2008^50^ | X | X |  | (X) |  |  |  |  |  | (X) |  |  | (X) | X | (X) |  |  |  |
| Senagore, 2009^51^ |  | X | (X) | (X) |  |  |  |  |  |  |  | (X) |  | X | (X) |  |  | X |
| Benes, 2010^52^ | X | X | X |  | X |  | (X) | (X) | (X) |  | X | (X) | X | X |  |  |  |  |
| Forget, 2010^53^ | X | X | X |  |  |  |  |  |  | (X) |  |  |  | X |  |  | X |  |
| Mayer, 2010^54^ | X | X | X |  | X | (X) |  |  | (X) |  | X | (X) | (X) | X |  | X | X |  |
| Van der Linden, 2010^55^ | (only colloid reported) | X | X |  | X |  |  |  |  |  |  |  |  |  |  |  |  |  |
| Pillai, 2011^56^ |  |  | X |  |  |  |  |  |  |  |  |  |  | X |  | X |  |  |
| Brand-strup, 2012^57^ | X | X | X |  | X | (X) |  | (X) | X |  | X | (X) |  | X |  | X | X |  |
| Challand, 2012^58^ | X | X | X |  |  |  |  |  |  |  |  |  | X |  |  |  |  |  |
| Zhang, 2012^59^ | X | (X) | X |  | X |  |  |  |  |  |  | (X) |  | X |  | X |  |  |
| Bisgaard, 2013^60^ | X | X | X |  | X | (X) |  | (X) | (X) |  | X | (X) | (X) | X |  | X |  |  |
| Bundgaard-Nielsen, 2013^61^ | X |  | X |  |  |  |  |  |  |  |  |  |  |  |  |  |  |  |
| El Sharkawy, 2013^62^ | (X) | (X) | (X) |  | X |  |  |  |  |  |  |  |  |  |  |  |  |  |
| McKenny, 2013^63^ | X | (X) | X |  | X | (X) |  |  | X |  | X | (X) |  | X |  |  |  |  |
| Ramsingh, 2013^64^ | (only colloid reported) |  | X |  |  |  |  |  |  |  |  |  |  |  |  |  |  |  |
| Salzwedel, 2013^65^ | X |  | X | (X) |  |  |  |  |  | (X) |  |  | (X) |  | (X) |  |  |  |
| Scheeren, 2013^66^ | X | X |  |  |  |  |  |  |  |  |  |  |  | X |  |  |  |  |
| Srinivasa, 2013^67^ | X |  | X |  |  |  |  |  |  |  |  |  |  |  |  |  |  |  |
| Zakhaleva, 2013^68^ | X | (X) | X |  |  |  |  |  | X |  |  |  | (X) | X |  | (X) | X |  |
| Zheng, 2013^69^ | X |  | X |  |  |  |  |  |  | (X) | X | (X) |  |  |  |  |  |  |
| Pearse, 2014^70^ | X | X | X |  | X | X |  | X | X |  | X | (X) | X | X |  | X | X | X |
| Peng, 2014^71^ | X | X | X |  | X |  |  | (X) |  |  |  | (X) | (X) | X |  |  |  | X |
| Pestaña, 2014^72^ | X | X | X |  | X |  |  |  |  |  | (X) | (X) | X | X |  | X | X |  |
| Phan, 2014^73^ | X | X | X |  | X | (X) |  |  |  |  |  | (X) | (X) | X |  | (X) | X |  |
| Shillcutt, 2014^74^ |  |  | X |  |  | (X) |  |  |  |  | (X) | (X) | (X) |  |  |  |  |  |
| Benes, 2015^75^ | x | X | X | (X) |  |  |  |  |  | (X) |  |  | X |  |  |  |  |  |
| Col-antonio, 2015^76^ | X | X | (X) | (X) |  |  |  |  |  | (X) |  |  |  |  | (X) |  |  |  |
| Correa-Gallego, 2015^77^ | X | X | X |  |  | (X) |  |  |  |  |  |  | (X) | X |  |  |  |  |
| Funk, 2015^78^ | X | X | X | (X) | X |  |  |  |  |  | X | (X) | X |  |  |  |  | X |
| Jammer, 2015^79^ |  |  |  |  | X | (X) |  |  | (X) |  | (X) | (X) | (X) | (X) |  | X |  | X |
| Kumar, 2015^80^ | X | (X) | X |  | (X) | (X) | (X) |  | (X) |  | (X) | (X) | (X) | X |  | X | X |  |
| Lai, 2015^81^ | X | X | X |  |  |  |  |  |  |  |  |  | X |  |  |  | X |  |
| Broch, 2016^114^ | X |  | X |  |  |  |  |  |  |  |  |  |  |  |  |  |  |  |
| Hand, 2016^83^ | X |  | X |  |  |  |  |  |  |  |  |  |  |  |  |  |  |  |
| Kumar, 2016^84^ |  | (X) | X |  |  |  |  |  | (X) |  | (X) | (X) | (X) | X |  | X | X |  |
| Schmid, 2016^85^ | X | X |  | (X) |  |  |  |  |  | (X) | X |  | (X) |  |  |  |  | X |
| Elgendy, 2017^86^ | X | X | X |  |  |  |  |  |  |  |  |  |  |  |  |  |  |  |
| Gómez-Izquierdo, 2017^87^ | X | (X) | X | (X) | X | X |  |  | (X) | (X) | (X) | (X) | X | X | (X) | X | X | (X) |
| Liang, 2017^88^ | X | (X) | X |  | X | (X) |  |  |  |  |  | (X) | (X) |  |  |  |  | X |
| Luo, 2017^89^ | (only colloid reported) | X | X |  | X | (X) |  |  | (X) |  | (X) | (X) | (X) | (X) |  |  |  |  |
| Reisinger, 2017^90^ | X | X | X |  | X | (X) |  |  |  |  |  |  |  | X |  | (X) | X |  |
| Stens, 2017^91^ | X | X | X |  | X |  |  |  |  |  |  |  |  | X |  | (X) | X | X |
| Weinberg, 2017^92^ | X |  | X |  | X | (X) |  | X |  | (X) |  | X | X | X |  |  |  | X |
| Wu, 2017^93^ | X |  | X |  | X |  |  |  |  |  |  | (X) |  | X | (X) |  |  |  |
| Calvo-Vecino, 2018^7^ | X | X (180days) | X |  | X | (X) |  | X | (X) |  | X | X | X | X |  | X | X | X |
| Kaufmann, 2018^94^ |  |  | X | (X) |  |  |  |  |  | (X) |  |  | X | X |  |  |  |  |
| Kim, 2018^95^ | X |  | X |  | X | (X) |  |  | X |  |  | (X) |  | X |  |  |  |  |
| Yin, 2018^96^ | X |  | X | (X) |  |  |  |  |  | (X) |  |  |  |  |  |  |  |  |
| Zhang, 2018^97^ | X |  |  |  | X | (X) |  |  |  |  |  |  | (X) | X |  |  |  | X |
| Zhao, 2018^98^ | X |  | X |  | X | (X) |  |  |  |  |  | (X) |  | X |  |  |  |  |
| Cesur, 2019^99^ |  |  | X |  |  |  |  |  |  |  |  |  |  |  |  |  |  |  |
| Davies, 2019^100^ | X | X | X | (X) |  |  |  |  |  | (X) |  |  | (X) | X |  |  |  | X |
| Godai, 2019^101^ |  | (X) | X |  |  |  |  |  |  |  |  |  | (X) |  |  |  |  |  |
| Hasanin, 2019^102^ | X |  | X |  |  |  |  |  |  |  |  |  |  |  |  |  |  |  |
| Liu, 2019^103^ | X |  | X |  |  |  |  |  |  |  |  | (X) | X |  |  |  | X |  |
| Sujatha, 2019^104^ |  | X | X |  |  |  |  |  |  |  |  |  | X |  |  |  | X |  |
| Szturz, 2019^105^ | X | X | X |  | X | (X) |  |  | (X) |  | (X) | (X) | X | X |  | (X) |  | X |
| Weinberg, 2019^106^ | X |  | X |  | X | (X) |  | X |  | (X) |  | X | X | X |  |  |  | X |
| Arslan-Carlon, 2020^107^ | X |  | X | (X) |  |  |  |  | X |  |  | (X) | (X) | X |  | X |  |  |
| De Cassai, 2020^108^ | X |  | X |  |  | (X) |  |  |  |  | (X) |  |  |  |  |  |  |  |
| Fischer, 2020^109^ |  | (X) | X |  |  |  |  | (X) |  |  | X | (X) | (X) | X |  | (X) |  |  |
| Iwasaki, 2020^110^ | X | (X) |  |  |  |  |  |  |  |  |  |  |  |  |  |  |  |  |
| Nicklas, 2020^111^ | X | X |  |  | X | (X) |  | (X) | X |  | (X) | (X) | X | X |  | x | X | X |
| Schneck, 2020^112^ | X |  | X |  |  |  |  |  |  |  |  |  |  |  |  |  |  |  |
| Diaper, 2021^113^ | X | X | X | (X) | X |  |  | (X) |  | (X) | X | (X) | X | X |  |  |  | X |
| (X): Outcome is reported but not included in the primary meta-analyses either due to 1) incomparable outcome definition, 2) no events in either group, or 3) continuous outcome that could not be transformed to a mean and standard deviation  ^a-e^ Further detail is provided in the section “Outcomes: Definitions, data synthesis, and sensitivity analyses”  Abbreviations in alphabetical order: **ARDS:** Acute respiratory distress syndrome | | | | | | | | | | | | | | | | | | |

## eTable 5: Risk of bias for individual trials

| **Study, Year of publication** | **Randomization** | **Adherence to intervention** | **Missing outcome data** | **Measurement of the outcome** | **Selective reporting** | **Overall** |
| --- | --- | --- | --- | --- | --- | --- |
| Shoemaker, 1988^15^ | Intermediate^a^ | Intermediate | Low | Intermediate | Intermediate | Intermediate |
| Bender, 1997^43^ | Intermediate^b^ | Intermediate^c^ | Low | Intermediate | Low | Intermediate |
| Sinclair, 1997^44^ | Low | Intermediate | Low | Low | Intermediate | Intermediate |
| Conway, 2002^45^ | Low | Intermediate | Low | Low | Intermediate | Intermediate |
| Gan, 2002^46^ | Low | Intermediate | Low | Low | Intermediate | Intermediate |
| Venn, 2002^35^ | Low | Intermediate | Low | Low | Intermediate | Intermediate |
| Wakeling, 2005^47^ | Low | Intermediate | Low | Low | Intermediate | Intermediate |
| Noblett, 2006^48^ | Low | Intermediate | Low | Low | Intermediate | Intermediate |
| Lopes, 2007^49^ | Low | Intermediate | Low | Low | Intermediate | Intermediate |
| Buettner, 2008^8^ | Low | Intermediate | Low | Low | Intermediate | Intermediate |
| Harten, 2008^50^ | Low | Intermediate | Low | Low | Low | Intermediate |
| Senagore, 2009^51^ | Low | Intermediate | Low | Low | Intermediate | Intermediate |
| Benes, 2010^52^ | Low | Intermediate | Low | Low | Low | Intermediate |
| Forget, 2010^53^ | Intermediate^b^ | Intermediate | Low | Low | High^d^ | High |
| Mayer, 2010^54^ | Low | Intermediate | Low | Low | Intermediate^e^ | Intermediate |
| Van der Linden, 2010^55^ | Low | Intermediate | Low | Low | Intermediate | Intermediate |
| Pillai, 2011^56^ | Intermediate^b^ | Intermediate | Low | Intermediate | Low | Intermediate |
| Brandstrup, 2012^57^ | Low | Intermediate | Low | Low | Intermediate | Intermediate |
| Challand, 2012^58^ | Low | Intermediate | Low | Low | Low | Intermediate |
| Zhang, 2012^59^ | Low | Intermediate | Low | Low | Intermediate | Intermediate |
| Bisgaard, 2013^60^ | Low | Intermediate | Low | Low | Low | Intermediate |
| Bundgaard-Nielsen, 2013^61^ | Low | Intermediate | Low | Low | Low | Intermediate |
| El Sharkawy, 2013^62^ | Low | Intermediate | High^f^ | Intermediate^g^ | Intermediate | High |
| McKenny, 2013^63^ | Low | Intermediate | Low | Low | Intermediate | Intermediate |
| Ramsingh, 2013^64^ | Low | Intermediate | Intermediate^h^ | Low | Intermediate^e^ | Intermediate |
| Salzwedel, 2013^65^ | Low | Intermediate | Low | Low | Low | Intermediate |
| Scheeren, 2013^66^ | Low | Intermediate | Low | Low | Low | Intermediate |
| Srinivasa, 2013^67^ | Low | Intermediate | Low | Low | Low | Intermediate |
| Zakhaleva, 2013^68^ | Low | Intermediate | Intermediate^h^ | Low | Low | Intermediate |
| Zheng, 2013^69^ | Low | Intermediate | Low | Low | Intermediate^e^ | Intermediate |
| Pearse, 2014^70^ | Low | Intermediate | Low | Low | Low | Intermediate |
| Peng, 2014^71^ | Low | Intermediate | Low | Low | Intermediate | Intermediate |
| Pestaña, 2014^72^ | Low | Intermediate | Low | Low | Low | Intermediate |
| Phan, 2014^73^ | Low | Intermediate | Low | Low | Low | Intermediate |
| Shillcutt, 2014^74^ | Low | Intermediate | Low | Low | Intermediate | Intermediate |
| Benes, 2015^75^ | Low | Intermediate | Low | Low | Low | Intermediate |
| Colantonio, 2015^76^ | Low | Intermediate | Low | Low | Low | Intermediate |
| Correa-Gallego, 2015^77^ | Low | Intermediate | Low | Low | Intermediate | Intermediate |
| Funk, 2015^78^ | Low | Intermediate | Low | Low | Low | Intermediate |
| Jammer, 2015^79^ | Low | High^i^ | Low | Low | Intermediate^j^ | High |
| Kumar, 2015^80^ | Intermediate^b^ | Intermediate | Low | Low | Intermediate | Intermediate |
| Lai, 2015^81^ | Low | Intermediate | Low | Low | Low | Intermediate |
| Broch, 2016^114^ | Intermediate^b^ | Intermediate | Low | Low | Intermediate | Intermediate |
| Hand, 2016^83^ | Low | Intermediate | Low | Low | Low | Intermediate |
| Kumar, 2016^84^ | Low | Intermediate | Low | Low | Intermediate | Intermediate |
| Schmid, 2016^85^ | Low | Intermediate | Low | Low | Low | Intermediate |
| Elgendy, 2017^86^ | Low | Intermediate | Low | Low | Intermediate | Intermediate |
| Gómez-Izquierdo, 2017^87^ | Low | Intermediate | Low | Low | Low | Intermediate |
| Liang, 2017^88^ | Low | Intermediate | Low | Low | Intermediate^e^ | Intermediate |
| Luo, 2017^89^ | Low | Intermediate | Low | Low | Intermediate | Intermediate |
| Reisinger, 2017^90^ | Low | Intermediate | Low | Low | Low | Intermediate |
| Stens, 2017^91^ | Low | Intermediate | Intermediate^h^ | Low | Low | Intermediate |
| Weinberg, 2017^92^ | Low | Intermediate | Low | Low | Low | Intermediate |
| Wu, 2017^93^ | Low | Intermediate | Low | Low | Intermediate | Intermediate |
| Calvo-Vecino, 2018^7^ | Low | Intermediate | Low | Low | Low | Intermediate |
| Kaufmann, 2018^94^ | Low | Intermediate | Low | Low | Low | Intermediate |
| Kim, 2018^95^ | Low | Intermediate | Low | Low | Low | Intermediate |
| Yin, 2018^96^ | Low | Intermediate | Low | Low | Low | Intermediate |
| Zhang, 2018^97^ | Intermediate^k^ | Intermediate | Low | Low | Low | Intermediate |
| Zhao, 2018^98^ | Low | Intermediate | Low | Low | Intermediate | Intermediate |
| Cesur, 2019^99^ | Low | Intermediate | Low | Low | Low | Intermediate |
| Davies, 2019^100^ | Low | Intermediate | Low | Low | Low | Intermediate |
| Godai, 2019^101^ | Low | Intermediate | Low | Low | Low | Intermediate |
| Hasanin, 2019^102^ | Low | Intermediate | Low | Low | Low | Intermediate |
| Liu, 2019^103^ | Low | Intermediate | Low | Low | Low | Intermediate |
| Sujatha, 2019^104^ | Low | Intermediate | Low | Low | Intermediate | Intermediate |
| Szturz, 2019^105^ | Low | Intermediate | Low | Low | Low | Intermediate |
| Weinberg, 2019^106^ | Low | Intermediate | Low | Low | Low | Intermediate |
| Arslan-Carlon, 2020^107^ | Low | Intermediate | Low | Low | Low | Intermediate |
| De Cassai, 2020^108^ | Low | Intermediate | Low | Low | Intermediate | Intermediate |
| Fischer, 2020^109^ | Low | Intermediate | Low | Low | Low | Intermediate |
| Iwasaki, 2020^110^ | Low | Intermediate | Low | Low | Low | Intermediate |
| Nicklas, 2020^111^ | Intermediate^k^ | Intermediate | Low | Low | Low | Intermediate |
| Schneck, 2020^112^ | Low | Intermediate | Low | Low | Low | Intermediate |
| Diaper, 2021^113^ | Low | Intermediate | Low | Low | Low | Intermediate |
| Risk of bias for all included trials, assessed using ”Revised Cochrane risk-of-bias tool for randomized trials (RoB 2)”^1^  See segment “Risk of bias assessment” for general considerations on each item. If no footnote is provided, then the assessment is based on the general considerations.  ^a^ Limited baseline data with some imbalances  ^b^ Randomization process not fully described  ^c^ Outcome assessors not blinded  ^d^ Reported a significant primary outcome that was different from the one described in the trial registration  ^e^ Some discrepancies between trial registration and manuscript  ^f^ Does not provide sample size for most outcomes. Large gap between sample size calculation and the sample size stated for one outcome.  ^g^ Primary outcome not defined. Not clear who assessed the outcome.  ^h^ Missing outcome data or loss to follow-up in a large proportion of patients  ^i^ Very limited information and data provided  ^j^ Stopped early with change in analysis plan  ^k^ Most likely no allocation concealment | | | | | | |

## eTable 6. Primary analyses and sensitivity analyses

| **Definition of analysis** | **Number of trials^a^** | **Number of patients** | | **Hetero-geneity (I^2^ (%))** | **Effect estimate**  **(95% CI)** | **Forest plot figure** |
| --- | --- | --- | --- | --- | --- | --- |
|  |  | **Intervention**  **(Events/Total (%))** | **Standard Care**  **(Events/Total (%))** |  |  |  |
| **Mortality** | | | | | | |
| Primary analysis | 39 | 124/2723 (4.5) | 134/2604 (5.1) | 0 | OR: 0.84 (0.64, 1.09) | eFigure 3 |
| Excluding trials with high risk of bias | 38 | 122/2682 (4.6) | 134/2563 (5.2) | 0 | OR: 0.82 (0.63, 1.07) | eFigure 4 |
| Excluding outlying outcome definition | 37 | 114/2474 (4.6) | 124/2353 (5.3) | 0 | OR: 0.84 (0.64, 1.09) | eFigure 5 |
| **Hospital length of stay (days)** | | | | | | |
| Primary analysis | 65 | n=3838 | n=3723 | 75 | Mean difference:  -0.72 (-1.10, -0.35) | eFigure 6 |
| Excluding trials with high risk of bias | 64 | n=3797 | n=3682 | 75 | Mean difference:  -0.72 (-1.10, -0.35) | eFigure 7 |
| Excluding trials with  > 20 days hospital length of stay in control group | 59 | n=3534 | n=3417 | 73 | Mean difference:  -0.67 (-1.03, -0.30) | eFigure 8 |
| **Pneumonia** | | | | | | |
| Primary analysis | 33 | 124/2122 (5.8) | 174/2103 (8.3) | 24 | OR: 0.69 (0.55, 0.88) | eFigure 35 |
| Excluding trials with high risk of bias | 31 | 117/2103 (5.6) | 173/2077 (8.3) | 0 | OR: 0.65 (0.51, 0.82) | eFigure 36 |
| Excluding trials without an outcome definition | 19 | 79/1272 (6.2) | 108/1294 (8.3) | 35 | OR: 0.73 (0.54, 0.98) | eFigure 37 |
| Only including trials with CDC 2008, EPCO 2015, NSQIP 2014, or ATS 2005-definitions | 8 | 50/812 (6.2) | 69/810 (8.5) | 9 | OR: 0.70 (0.48, 1.02) | eFigure 38 |
| **Pulmonary oedema** | | | | | | |
| Primary analysis (radiology AND clinical signs) | 2 | 2/430 (0.5) | 2/428 (0.5) | 25 | OR: 1.00 (0.14, 7.08) | eFigure 39 |
| Including all trials that reported the outcome | 21 | 23/1335 (1.7) | 50/1338 (3.7) | 47 | OR: 0.46 (0.29, 0.74) | eFigure 40 |
| **Pulmonary embolism** | | | | | | |
| Primary analysis | 9 | 13/834 (1.6) | 16/844 (1.9) | 32 | OR: 0.82 (0.39, 1.71) | eFigure 41 |
| Excluding trials without an outcome definition | 3 | 6/467 (1.3) | 1/472 (0.2) | 0 | OR: 4.23 (0.96, 18.66) | eFigure 42 |
| **Acute respiratory distress syndrome** | | | | | | |
| Primary analysis | 4 | 5/625 (0.8) | 18/625 (2.9) | 5 | OR: 0.32 (0.14, 0.74) | eFigure 43 |
| Including all trials that reported the outcome | 11 | 9/1177 (0.8) | 25/1173 (2.1) | 12 | OR: 0.39 (0.20, 0.76) | eFigure 44 |
| **Myocardial infarction** | | | | | | |
| Primary analysis | 13 | 29/1405 (2.1) | 32/1413 (2.3) | 9 | OR: 0.91 (0.55, 1.52) | eFigure 45 |
| Excluding trials without an outcome definition | 7 | 15/787 (1.9) | 21/789 (2.7) | 12 | OR: 0.71 (0.37, 1.39) | eFigure 46 |
| **Arrhythmia** | | | | | | |
| Primary analysis (EPCO 2015-criteria) | 3 | 6/259 (2.3) | 13/261 (5.0) | 0 | OR: 0.46 (0.18, 1.16) | eFigure 47 |
| Secondary analysis  (trials with definition “requiring intervention”) | 5 | 11/194 (5.7) | 11/195 (5.6) | 0 | OR: 1.02 (0.41, 2.51) | eFigure 48 |
| Secondary analysis:  Excluding trials with high risk of bias | 4 | 9/180 (5.0) | 10/179 (5.6) | 5 | OR: 0.88 (0.33, 2.33) | eFigure 49 |
| Including all trials that reported the outcome | 34 | 106/1698 (6.2) | 125/1684 (7.4) | 0 | OR: 0.85 (0.66, 1.11) | eFigure 50 |
| **Acute kidney injury** | | | | | | |
| Primary analysis  (pre- to postoperative P-creatinine increase >150%) | 18 | 136/1722 (7.9) | 154/1622 (9.5) | 44 | OR: 0.82 (0.64, 1.05) | eFigure 51 |
| Including all trials that reported the outcome | 38 | 305/2545 (12.0) | 311/2444 (12.7) | 48 | OR: 0.94 (0.79, 1.13) | eFigure 52 |
| **Surgical site infection** | | | | | | |
| Primary analysis | 39 | 183/2346 (7.8) | 294/2301 (12.8) | 7 | OR: 0.54 (0.45, 0.66) | eFigure 53 |
| Excluding trials with high risk of bias | 38 | 175/2305 (7.6) | 286/2260 (12.7) | 7 | OR: 0.53 (0.44, 0.65) | eFigure 54 |
| Excluding trials without an outcome definition | 18 | 88/1295 (6.8) | 170/1312 (13.0)) | 0 | OR: 0.50 (0.38, 0.64) | eFigure 55 |
| Only including trials with EPCO 2015, CDC 2008, NSQIP 2014, or ATS 2005-definitions | 5 | 44/657 (6.7) | 86/657 (13.1) | 0 | OR: 0.49 (0.34, 0.70) | eFigure 56 |
| **Paralytic ileus** | | | | | | |
| Primary analysis | 15 | 108/1232 (8.8) | 127/1221 (10.4) | 24 | OR: 0.83 (0.63, 1.10) | eFigure 57 |
| Excluding trials with high risk of bias | 14 | 107/1218 (8.8) | 125/1205 (10.4) | 29 | OR: 0.84 (0.63, 1.11) | eFigure 58 |
| Including all trials that reported the outcome | 22 | 157/1588 (9.9) | 187/1601 (11.7) | 40 | OR: 0.83 (0.66, 1.05) | eFigure 59 |
| **Anastomotic leakage** | | | | | | |
| Primary analysis | 21 | 55/1664 (3.3) | 81/1568 (5.2) | 8 | OR: 0.61 (0.43, 0.87) | eFigure 60 |
| Excluding trials with high risk of bias | 20 | 50/1624 (3.1) | 76/1528 (5.0) | 11 | OR 0.59 (0.41, 0.85) | eFigure 61 |
| Only including trials with a definition based on radiology | 6 | 29/815 (3.6) | 37/817 (4.5) | 22 | OR: 0.78 (0.47, 1.28) | eFigure 62 |
| **Delirium** | | | | | | |
| Primary analysis | 16 | 74/1502 (4.9) | 98/1492 (6.6) | 22 | OR: 0.72 (0.52, 0.99) | eFigure 63 |
| Excluding trials with high risk of bias | 15 | 73/1488 (5.6) | 96/1476 (7.2) | 21 | OR: 0.73 (0.53, 1.0) | eFigure 64 |
| Only including trials with EPCO 2015-definition | 3 | 3/259 (1.2) | 14/261 (5.4) | 0 | OR: 0.24 (0.09, 0.65) | eFigure 65 |
| Primary analysis and sensitivity analyses for all outcomes  *Only provided if there were trials in both abdominal surgery and non-abdominal surgery included in the analyses. The subgroups were not analyzed for the primary outcomes’ sensitivity analyses.  Abbreviations in alphabetical order: **CI**: Confidence interval, **OR:** Odds ratio | | | | | | |

## eTable 7: Subgroup analyses for mortality

| **Headline** | **Subgroup** | **Number of trials^a^** | **Number of patients** | | **Hetero-geneity (I^2^ (%))** | **Effect estimate:**  **Odds ratio (95% CI)^b^** | **Test for subgroup differences (P-value)** | **Forest plot figure** |
| --- | --- | --- | --- | --- | --- | --- | --- | --- |
|  |  |  | **Intervention**  **(Events/Total (%))** | **Standard Care**  **(Events/Total (%))** |  |  |  |  |
| **Mortality** | | | | | | | | |
| Risk of surgery | Moderate risk of surgery | 2 | 1/82 (1.2) | 1/62 (1.6) | 32 | 0.72 (0.04, 12.4) | 0.55 | eFigure 9 |
|  | High risk of surgery | 21 | 53/1782 (3.0) | 45/1688 (2.7) | 0 | 1.00 (0.66, 1.50) |  |  |
|  | Very high risk of surgery | 16 | 70/859 (8.2) | 88/854 (10.3) | 0 | 0.74 (0.53, 1.05) |  |  |
| Surgery type^c^ | Abdominal | 30 | 91/2279 (4.0) | 103/2183 (4.7) | 0 | 0.82 (0.62, 1.10) | 0.80 | eFigure 10 |
|  | Non-abdominal | 8 | 23/414 (5.6) | 24/391 (6.1) | 0 | 0.90 (0.49, 1.63) |  |  |
| Surgery technique^d^ | Open | 20 | 58/1202 (4.8) | 64/1120 (5.7) | 0 | 0.75 (0.51, 1.12) | 0.54 | eFigure 11 |
|  | Laparoscopic | 5 | 13/453 (2.9) | 13/446 (2.9) | 0 | 0.99 (0.45, 2.17) |  |  |
| Concept of preload variation in GDHT-protocol | Respiratory cycle | 17 | 55/844 (6.2) | 69/797 (8.7) | 0 | 0.64 (0.43, 0.95) | 0.15 | eFigure 12 |
|  | Fluid challenges | 15 | 47/1230 (3.8) | 47/1198 (3.9) | 0 | 0.97 (0.64, 1.47) |  |  |
| Use of inotropes/vasopressors in GDHT-protocol | No inotropes/  vasopressor | 19 | 52/1182 (4.4) | 60/1171 (5.1) | 0 | 0.82 (0.54  , 1.23) | 0.86 | eFigure 13 |
|  | Inotropes and/or vasopressor | 20 | 72/1541 (4.7) | 74/1433 (5.2) | 0 | 0.85 (0.61, 1.20) |  |  |
| Intra-operative fluid amount difference between GDHT and standard care | GDHT protocol resulting in more fluid | 8 | 12/528 (2.3) | 15/535 (2.8) | 0 | 0.78 (0.36, 1.70) | 0.98 | eFigure 14 |
|  | Similar fluid volumes | 20 | 53/1582 (3.3) | 65/1596 (4.1) | 0 | 0.74 (0.51, 1.05) |  |  |
|  | GDHT protocol resulting in less fluid | 3 | 26/148 (17.6) | 28/149  (18.8) | 64 | 0.86 (0.45, 1.63) |  |  |
| Type of device used in GDHT-protocol | Non-invasive | 5 | 21/413 (5.1) | 20/374 (5.3) | 0 | 0.88 (0.47, 1.68) | 0.92 | eFigure 15 |
|  | Pulse Contour Analysis | 21 | 74/1540 (4.8) | 85/1466 (5.8) | 0 | 0.77 (0.55, 1.08) |  |  |
|  | Oesophageal Doppler monitoring | 11 | 18/689 (2.6) | 21/681 (3.1) | 2.5 | 0.85 (0.45, 1.60) |  |  |
| Type of fluid used in GDHT-protocol | Crystalloids | 3 | 13/242 (5.4) | 12/242 (5.0) | 0 | 1.09 (0.48, 2.46) | 0.67 | eFigure 16 |
|  | Colloids | 27 | 84/1719 (4.9) | 95/1603 (5.9) | 0 | 0.76 (0.55, 1.05) |  |  |
| ^a^ Number of trials used in analysis (number of trials with reported outcome including trials without events)  ^b^ Peto’s method for odds ratio used for all binary outcomes with 95% confidence interval  ^c^ Abdominal surgery includes all types of surgery in the abdomen including gastrointestinal, gynaecology, urology, hepatobiliary, pancreatic etc.  ^d^ Surgery type: Laparoscopic if > 50% of patients operated with laparoscopic technique, only trials classified as “abdominal” included  ^e^ Random effects (DerSimonian and Laird) used for continuous outcomes of mean differences with 95% confidence interval  Abbreviations in alphabetical order: **CI:** Confidence interval, **GDHT:** Goal-directed haemodynamic therapy | | | | | | | | |

## eTable 8: Subgroup analyses for hospital length of stay

| **Headline** | **Subgroup** | **Number of trials^a^** | **Number of patients in GDHT-arm (*n*)** | **Number of patients in standard care-arm (*n*)** | **Hetero-geneity (I^2^ (%))** | **Effect estimate**  **Mean difference (95% CI)^e^** | **Test for subgroup differences (P-value)** | **Forest plot figure** |
| --- | --- | --- | --- | --- | --- | --- | --- | --- |
| **Hospital length of stay** | | | | | | | | |
| Risk of surgery | Moderate risk of surgery | 9 | 305 | 284 | 76 | -0.41 (-1.0, 0.17) | 0.22 | eFigure 17 |
|  | High risk of surgery | 37 | 2692 | 2593 | 63 | -0.58 (-1.06, -0.10) |  |  |
|  | Very high risk of surgery | 19 | 841 | 846 | 85 | -1.45 (-2.47, -0.43) |  |  |
| Surgery type^c^ | Abdominal | 49 | 2960 | 2878 | 75 | -0.78 (-1.25, -0.32) | 0.39 | eFigure 18 |
|  | Non-abdominal | 15 | 848 | 815 | 60 | -0.45 (-1.04, 0.13) |  |  |
| Surgery technique^d^ | Open | 33 | 1796 | 1721 | 60 | -0.34 (-0.79, 0.11) | 0.09 | eFigure 19 |
|  | Laparoscopic | 8 | 574 | 568 | 89 | -1.45 (-2.64, -0.25) |  |  |
| Concept of preload variation in GDHT-protocol | Respiratory cycle | 35 | 1625 | 1510 | 67 | -0.92 (-1.37, -0.47) | 0.29 | eFigure 20 |
|  | Fluid challenges | 22 | 1540 | 1537 | 74 | -0.46 (-1.18, 0.27) |  |  |
| Use of inotropes/vasopressors in GDHT-protocol | No inotropes/  vasopressor | 28 | 1572 | 1573 | 74 | -0.45 (-1.02, 0.12) | 0.20 | eFigure 21 |
|  | Inotropes and/or vasopressor | 37 | 2266 | 2150 | 76 | -0.94 (-1.45, -0.44) |  |  |
| Intra-operative fluid amount difference between GDHT and standard care | GDHT protocol resulting in more fluid | 12 | 693 | 681 | 78 | -0.42 (-1.53, 0.69) | 0.44 | eFigure 22 |
|  | Similar fluid volumes | 29 | 1852 | 1875 | 63 | -0.82 (-1.32, -0.32) |  |  |
|  | GDHT protocol resulting in less fluid | 9 | 457 | 455 | 85 | -1.44 (-2.57, -0.31) |  |  |
| Type of device | Non-invasive | 12 | 798 | 755 | 82 | -0.63 (-1.35, 0.09) | 0.79 | eFigure 23 |
|  | Pulse Contour Analysis | 32 | 1932 | 1836 | 95 | -0.93 (-1.41, -0.45) |  |  |
|  | Oesophageal Doppler | 19 | 1027 | 1049 | 88 | -0.83 (-1.74, 0.07) |  |  |
| Type of fluid | Crystalloids | 8 | 403 | 383 | 84 | -1.58 (-2.83, -0.33) | 0.50 | eFigure 24 |
|  | Colloids | 39 | 2212 | 2088 | 75 | -0.63 (-1.12, -0.14) |  |  |
| ^a^ Number of trials used in analysis (number of trials with reported outcome including trials without events)  ^b^ Peto’s method for odds ratio used for all binary outcomes with 95% confidence interval  ^c^ Abdominal surgery includes all types of surgery in the abdomen including gastrointestinal, gynaecology, urology, hepatobiliary, pancreatic etc.  ^d^ Surgery type: Laparoscopic if > 50% of patients operated with laparoscopic technique, only trials classified as “abdominal” included  ^e^ Random effects (DerSimonian and Laird) used for continuous outcomes of mean differences with 95% confidence interval  Abbreviations in alphabetical order: **CI:** Confidence interval, **GDHT:** Goal-directed haemodynamic therapy | | | | | | | | |

## eTable 9: Meta-regression analyses

| **Outcomes and moderators** | **Number of trials in analysis** | **Intercept** | **ß-coefficients** | **95 % CI** | **P-value** | **Bubble plot figure** |
| --- | --- | --- | --- | --- | --- | --- |
| **Mortality** | | | | | | |
| Year of patient inclusion | 39 | 0.22 | -0.02 | -0.06, 0.02 | 0.38 | eFigure 25 |
| Duration of surgery | 33 | 0.21 | -0.002 | -0.005, 0.001 | 0.25 | eFigure 26 |
| Mortality in control group | 39 | -0.14 | -0.003 | -0.026, 0.019 | 0.77 | eFigure 27 |
| Sample size in trials | 39 | -0.39 | 0.0009 | -0.0005, 0.002 | 0.20 | eFigure 28 |
| Hospital length of stay in control group | 32 | -0.21 | 0.008 | -0.043, 0.058 | 0.76 | eFigure 29 |
| **Hospital length of stay** | | | | | | |
| Year of patient inclusion | 65 | 0.61 | -0.055 | -0.115, 0.006 | 0.076 | eFigure 30 |
| Duration of surgery | 56 | -0.47 | -0.002 | -0.006, 0.003 | 0.47 | eFigure 31 |
| Sample size in trials | 65 | -0.84 | 0.0007 | -0.002, 0.003 | 0.59 | eFigure 32 |
| Mortality in control group | 42 | -0.48 | 0.01 | -0.047, 0.067 | 0.73 | eFigure 33 |
| Hospital length of stay in control group | 65 | 1.02 | -0.16 | -0.24, -0.09 | 0.000 | eFigure 34 |
| Summary of meta regression analyses investigating continuous variables as effects modifiers for the primary outcomes.  Abbreviations: **CI:** Confidence interval | | | | | | |

# eFIGURES

## eFigure 1: Flow chart of study selection


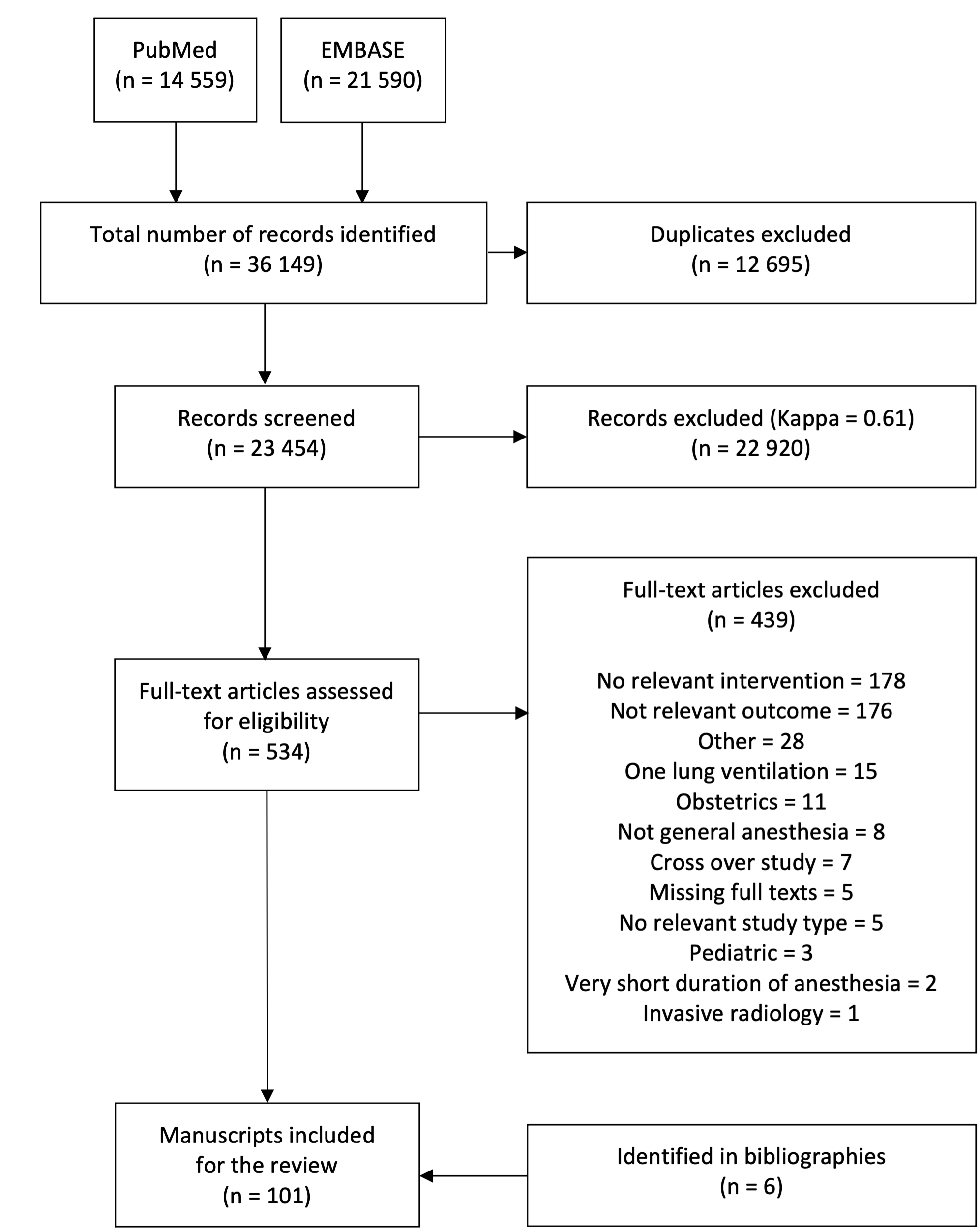


## eFigure 2: Intraoperative fluid volume differences between GDHT and standard care


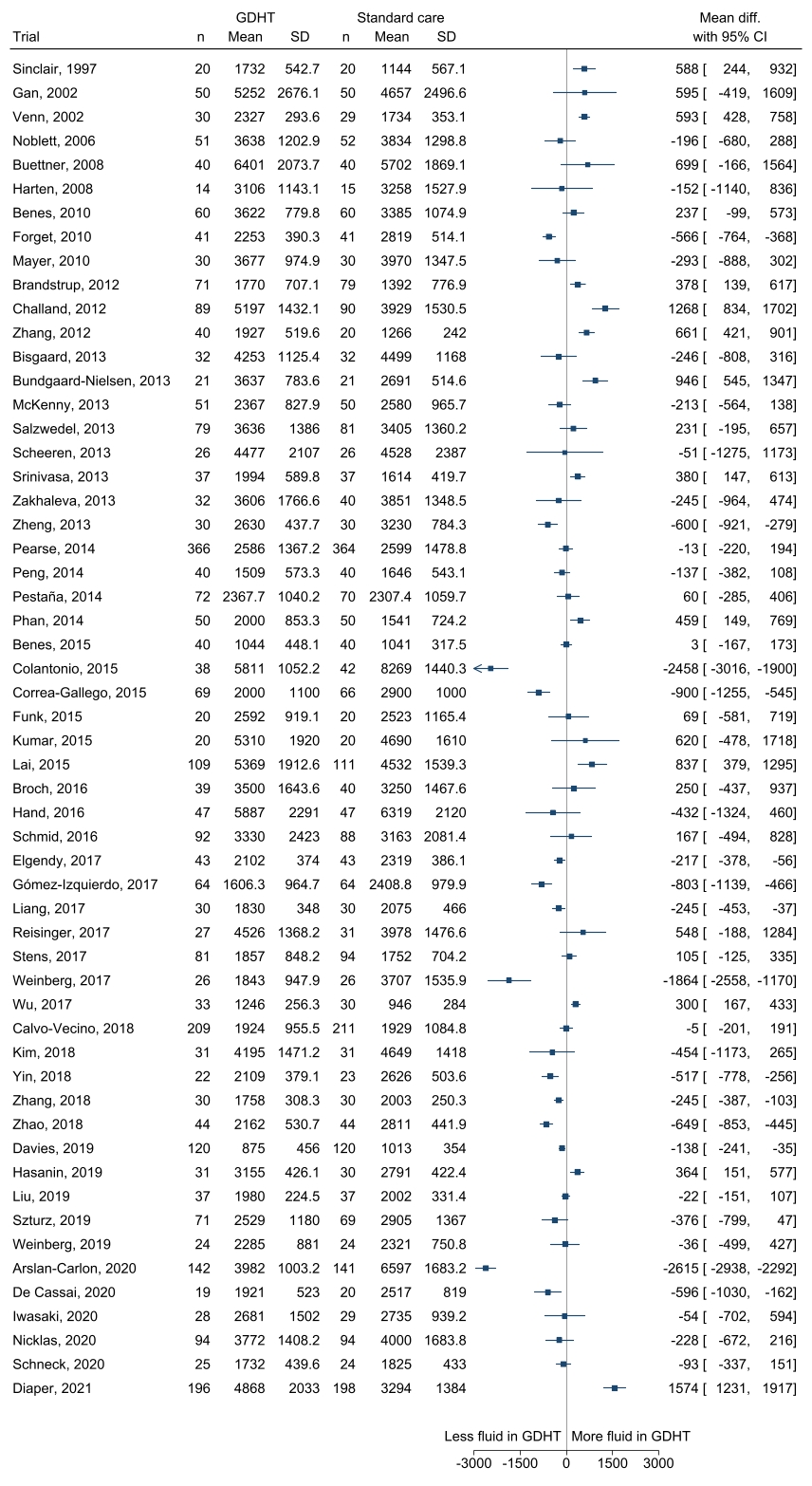


Mean fluid volume and standard deviations in ml. for GDHT and standard care arms are shown in numbers, and mean differences between the two groups are shown in the forest plot and as estimates with 95% confidence interval.

## Mortality, primary results

### eFigure 3: Mortality - Primary analysis


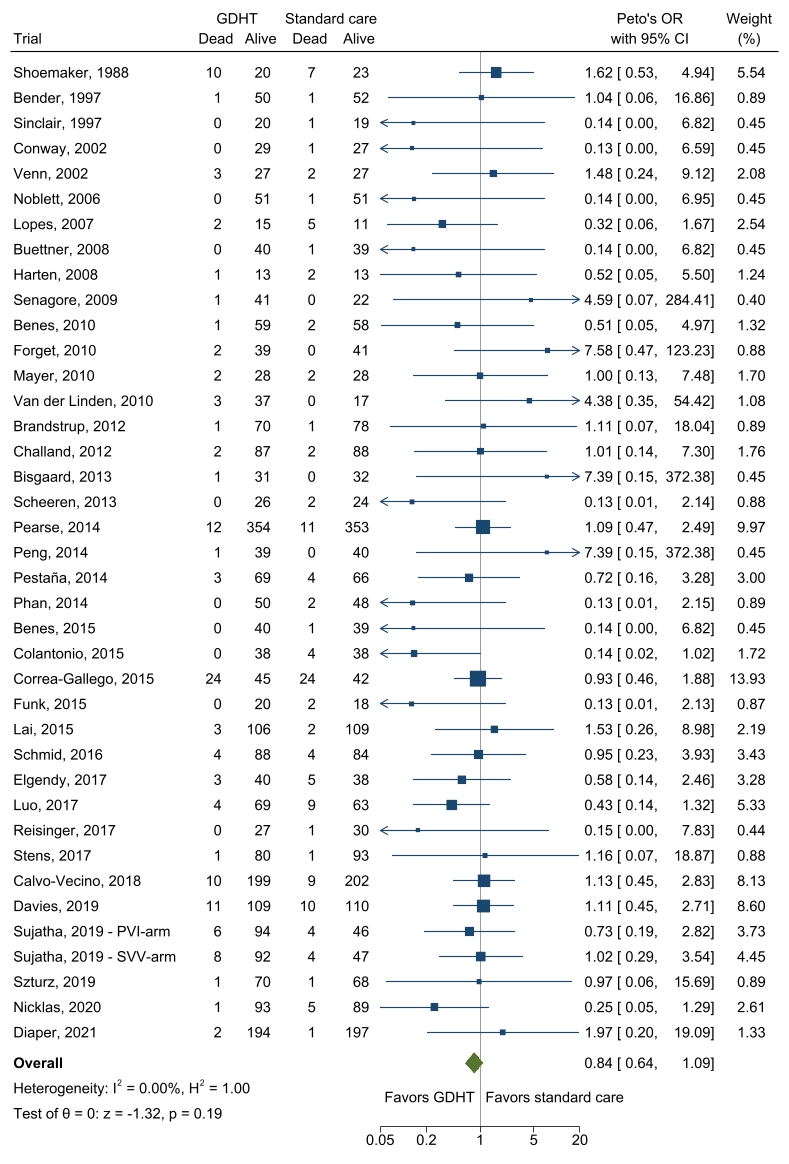


Fixed inverse-variance (Peto’s method for odds ratio) meta-analysis of GDHT compared to standard care for overall mortality.

### eFigure 4: Mortality - Sensitivity analysis 1: Excluding trials with high risk of bias


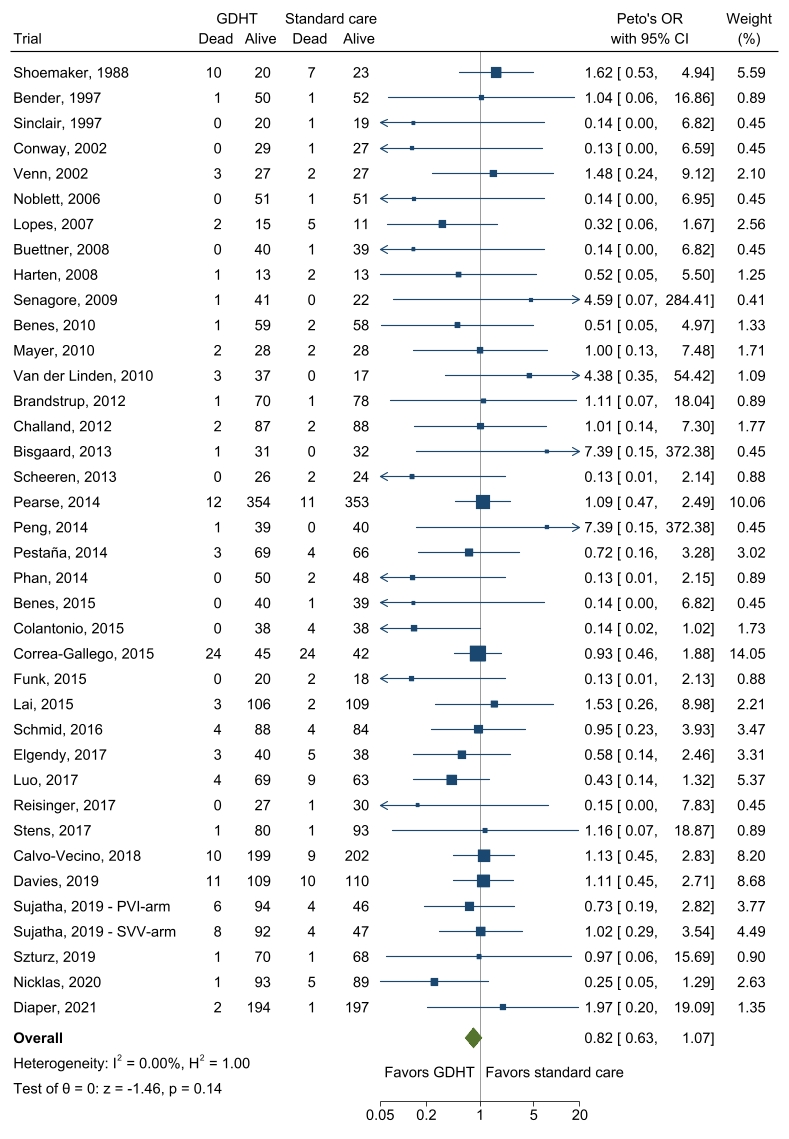


Fixed inverse-variance (Peto’s method for odds ratio) meta-analysis of GDHT compared to standard care for overall mortality. Trials with a high risk of bias were excluded.

### eFigure 5: Mortality - Sensitivity analysis 2: Excluding outlying outcome definition

**
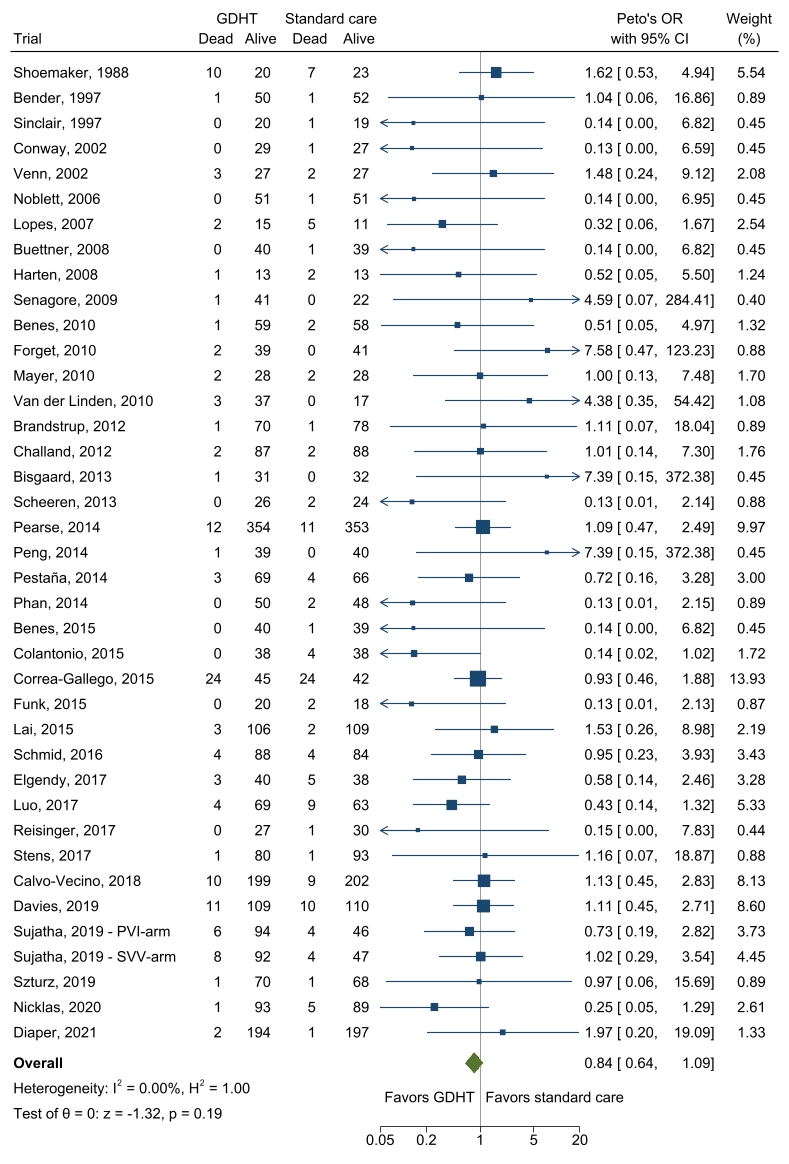
**

Fixed inverse-variance (Peto’s method for odds ratio) meta-analysis of GDHT compared to standard care for overall mortality. One trial reporting 180-day mortality was excluded.

## Hospital length of stay, primary results

### eFigure 6: Hospital length of stay - Primary analysis


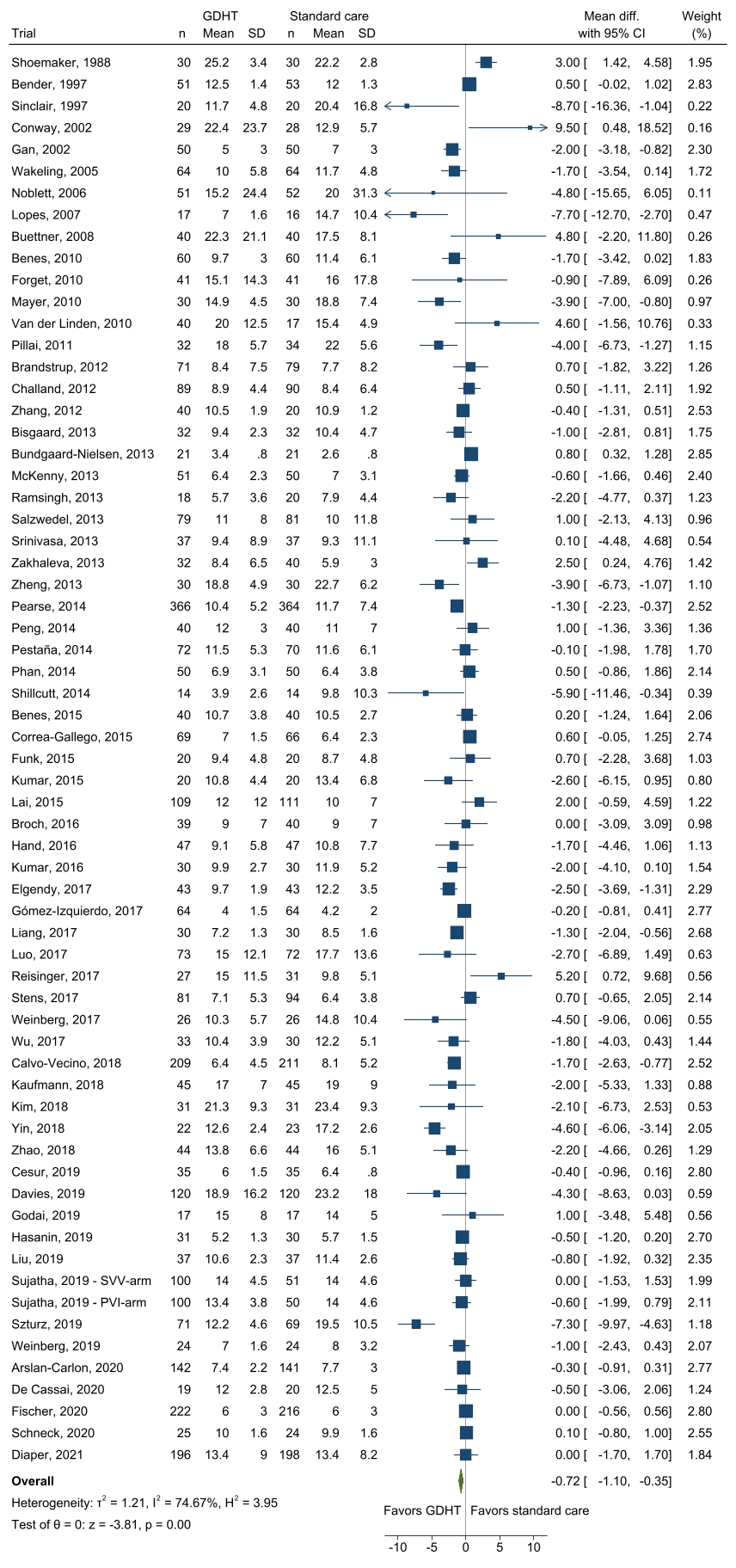


Random-effects meta-analysis of GDHT compared to standard care for hospital length of stay in days.

### eFigure 7: Hospital length of stay - Sensitivity analysis 1: Excluding trials with high risk of bias

**
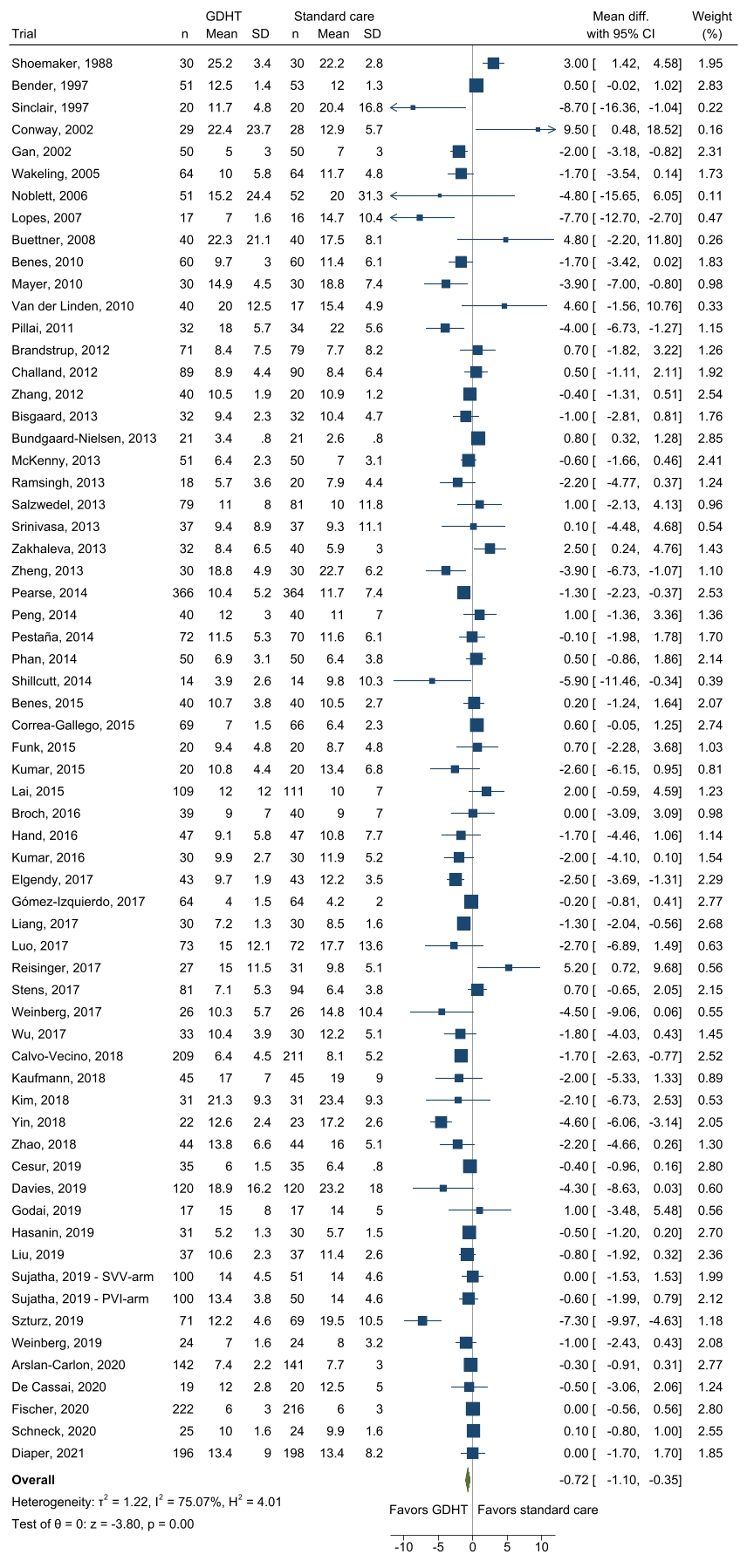
**

Random-effects meta-analysis of GDHT compared to standard care for hospital length of stay in days. Trials with a high risk of bias were excluded.

### eFigure 8: Hospital length of stay - Sensitivity analysis 2: Excluding trials with >20 days hospital length of stay in control group

**
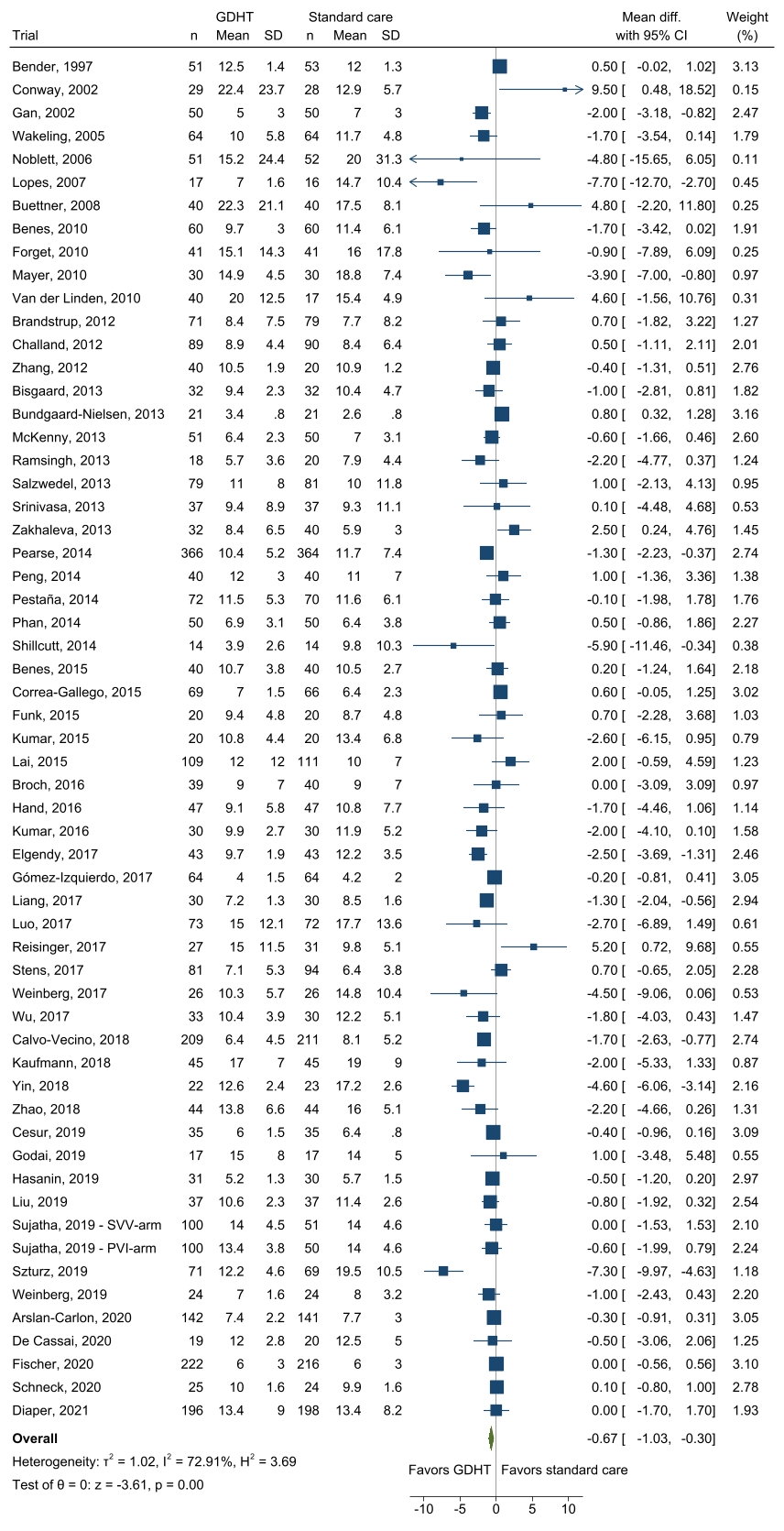
**

Random-effects meta-analysis of GDHT compared to standard care for hospital length of stay in days. Trials with > 20 days hospital length of stay in the control are excluded.

## Subgroup analyses, meta regressions, and funnel plots for mortality and hospital length of stay

### eFigure 9: Mortality - Subgroup analysis 1: Risk of surgery

**
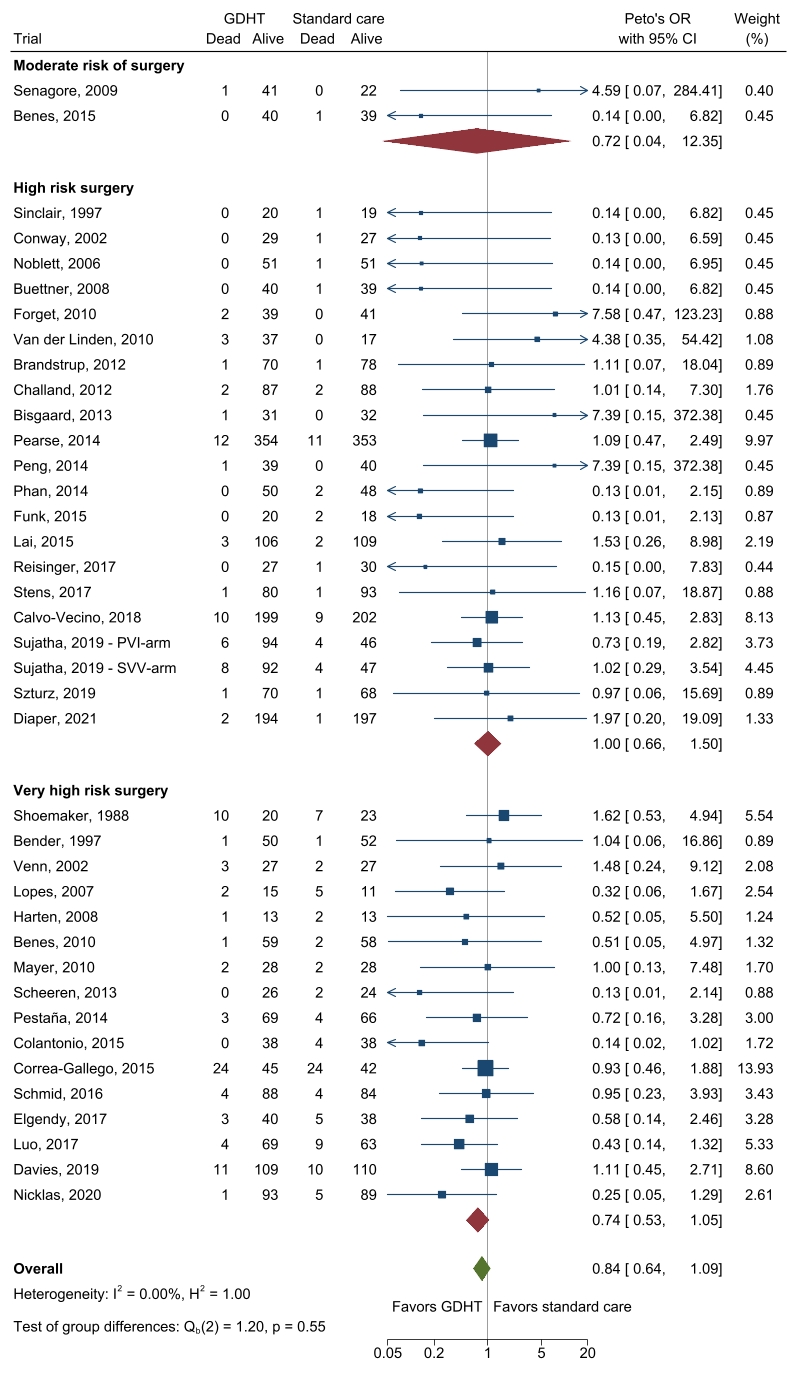
**

Fixed inverse-variance (Peto’s method for odds ratio) meta-analysis of GDHT compared to standard care for overall mortality. Trials are subgrouped according to risk of surgery. For definitions see section “Subgroup definitions”.

### eFigure 10: Mortality - Subgroup analysis 2: Abdominal surgery vs. non-abdominal surgery

**
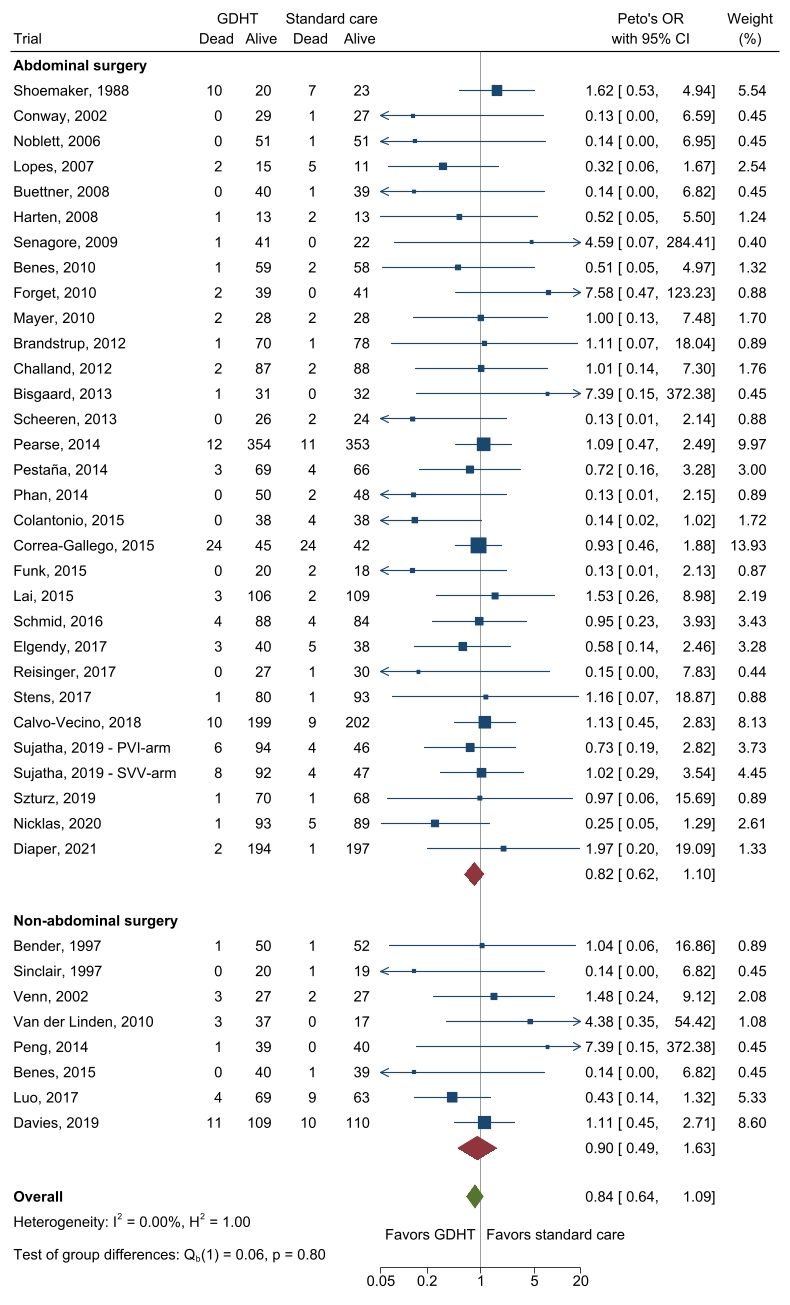
**

Fixed inverse-variance (Peto’s method for odds ratio) meta-analysis of GDHT compared to standard care for overall mortality. Trials are subgrouped as abdominal vs. non-abdominal. For definitions see section “Subgroup definitions”.

### eFigure 11: Mortality - Subgroup analysis 3: Open surgery vs. laparoscopic surgery

**
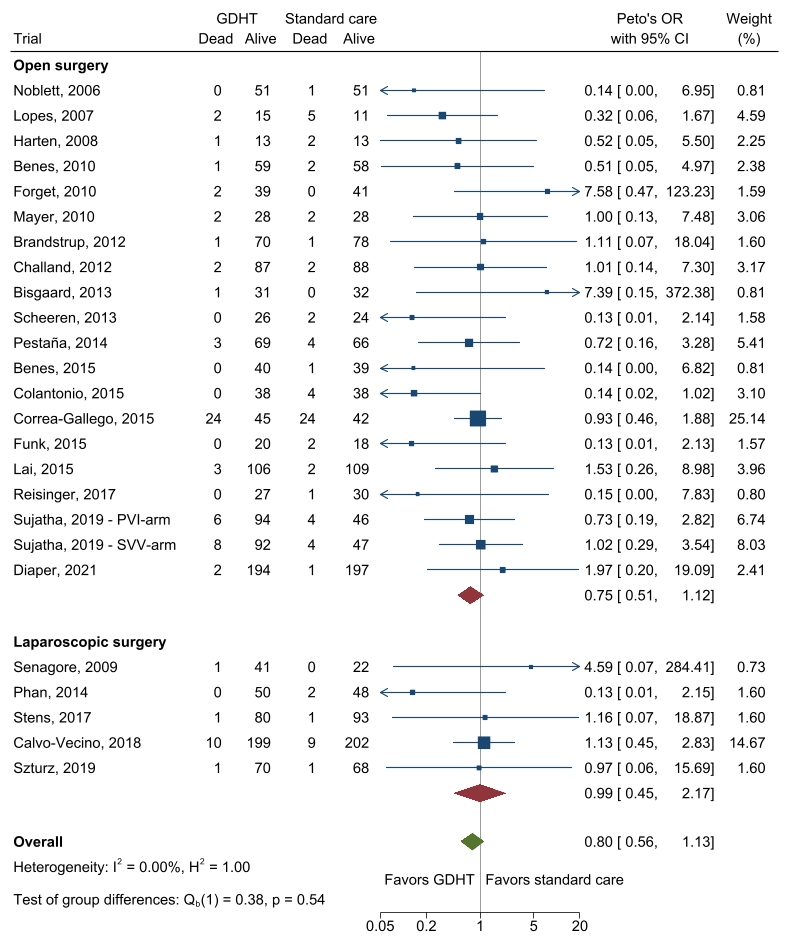
** Fixed inverse-variance (Peto’s method for odds ratio) meta-analysis of GDHT compared to standard care for overall mortality. Trials are subgrouped according to the surgical technique as open vs. laparoscopic. For definitions see section “Subgroup definitions”.

### eFigure 12: Mortality - Subgroup analysis 4: GDHT-targets based on preload variation by the respiratory cycle vs. fluid challenges


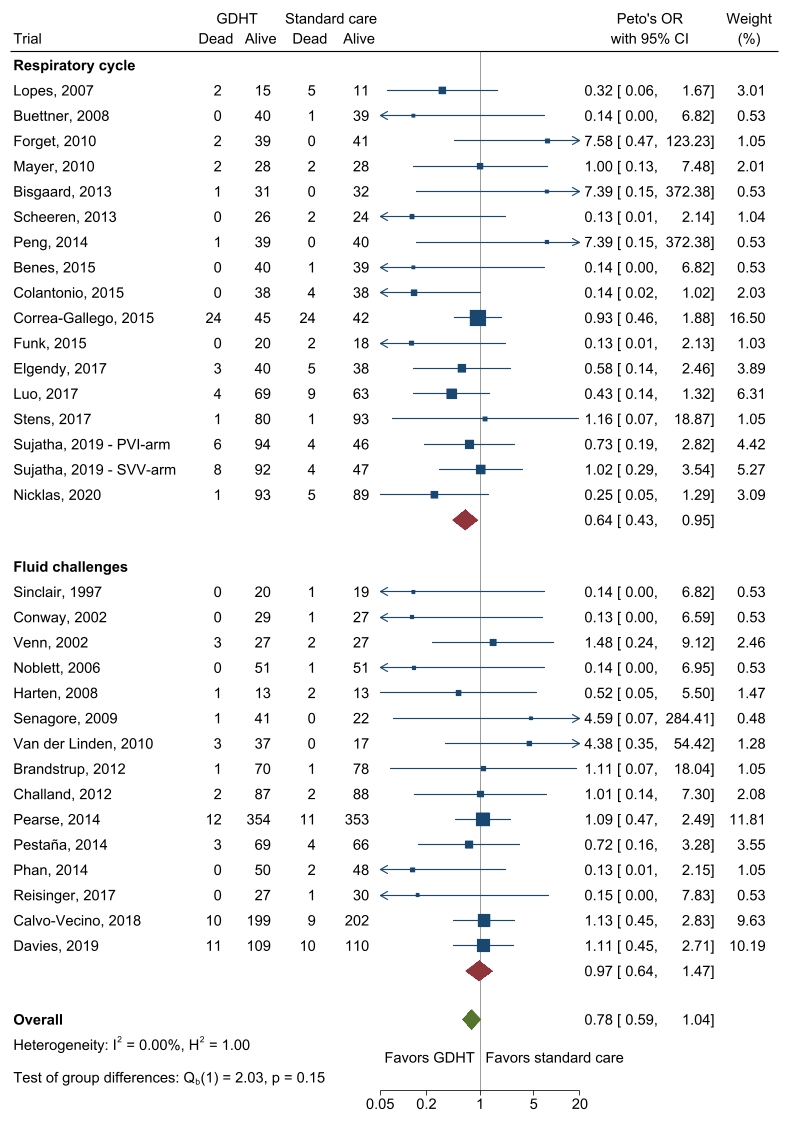


Fixed inverse-variance (Peto’s method for odds ratio) meta-analysis of GDHT compared to standard care for overall mortality. Trials are subgrouped according to concept of preload variation. For definitions see section “Subgroup definitions”.

### eFigure 13: Mortality - Subgroup analysis 5: GDHT-protocols without vs. with use of inotropes or vasopressors

**
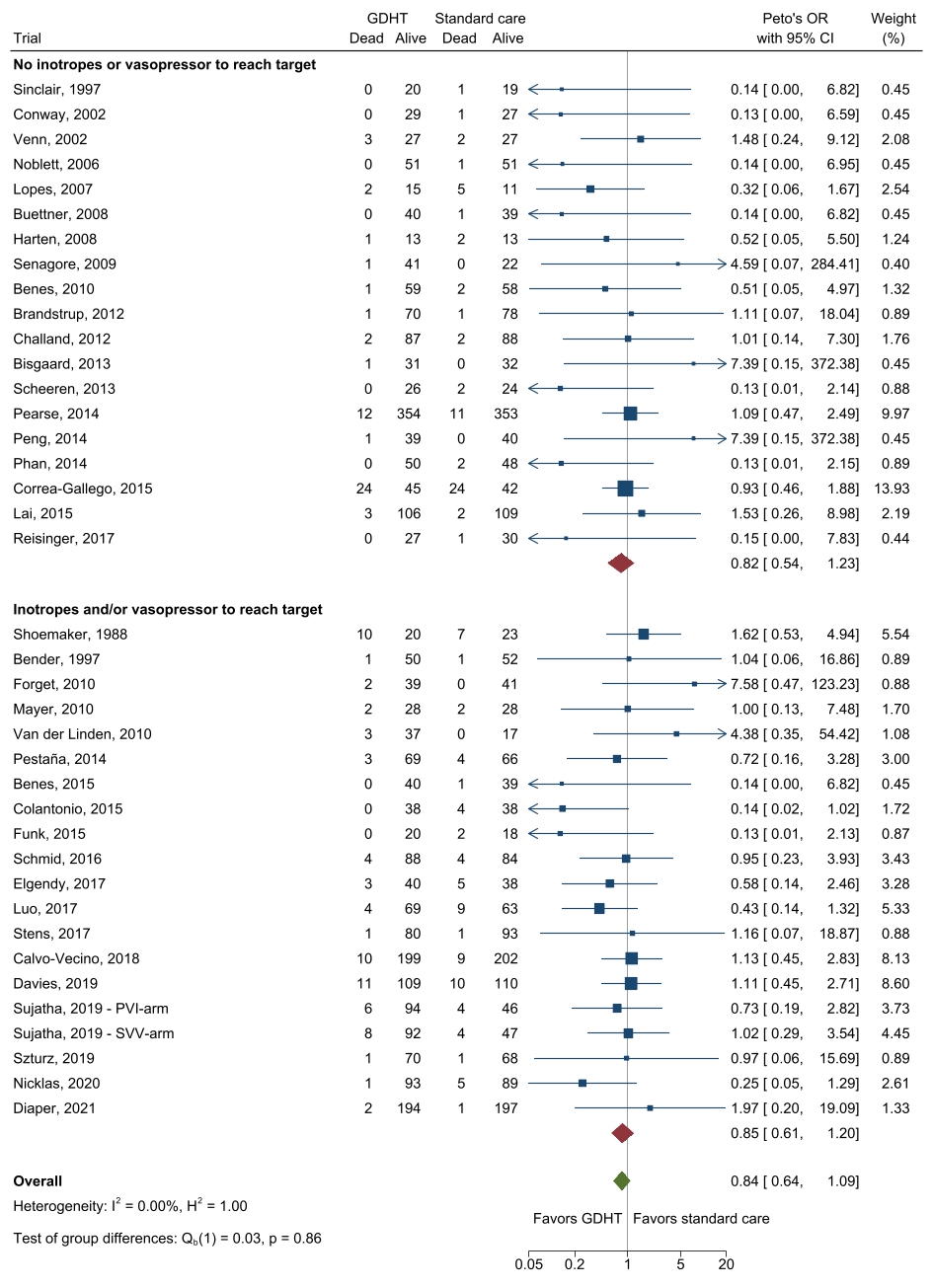
**

Fixed inverse-variance (Peto’s method for odds ratio) meta-analysis of GDHT compared to standard care for overall mortality. Trials are subgrouped according to the use of inotropes and/or vasopressors to reach targets. For definitions see section “Subgroup definitions”.

### eFigure 14: Mortality - Subgroup analysis 6: GDHT-protocols resulting in less vs. similar vs. more intraoperative fluid amounts than their standard care comparator


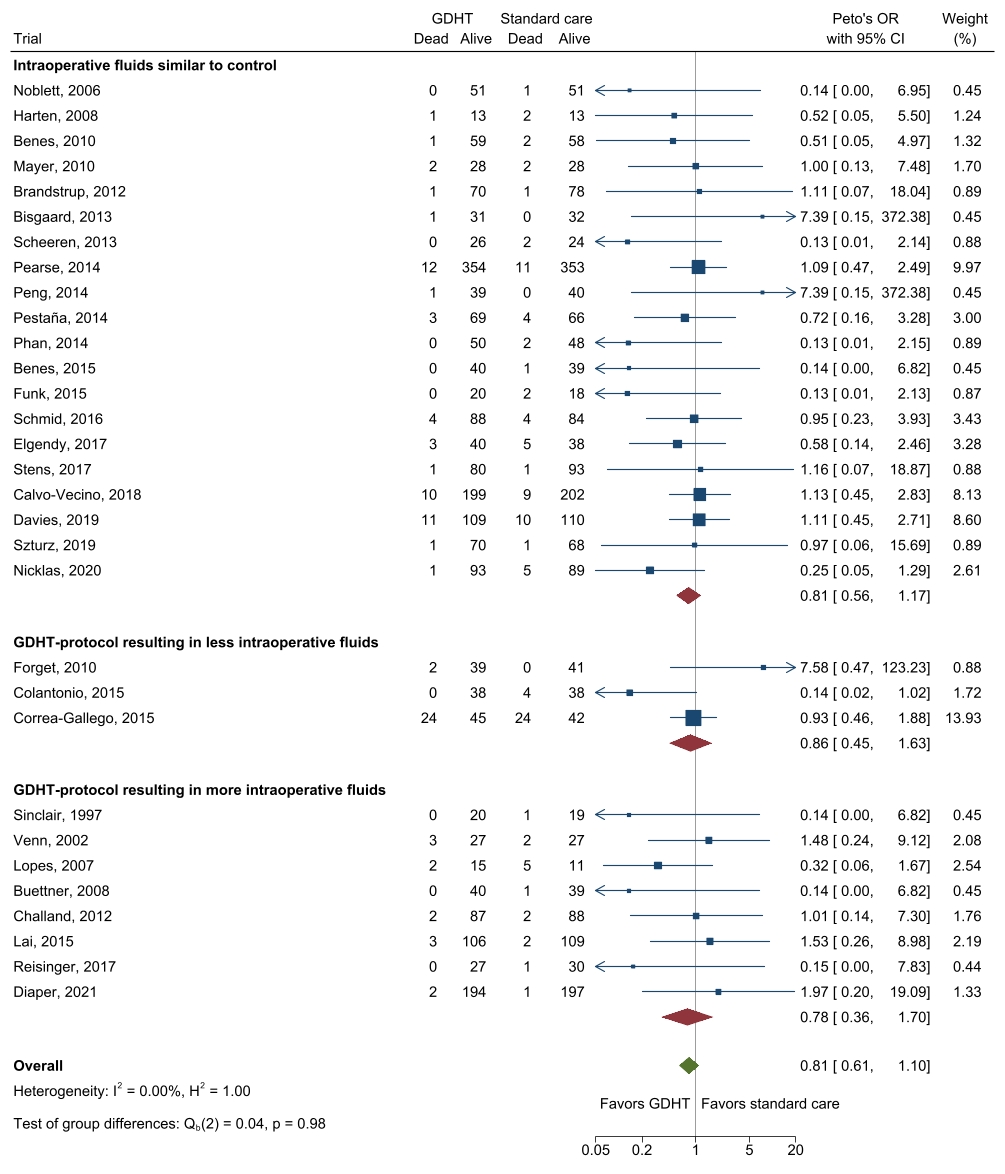


Fixed inverse-variance (Peto’s method for odds ratio) meta-analysis of GDHT compared to standard care for overall mortality. Trials are subgrouped according to intraoperative fluid volume differences in GDHT-arm vs standard care-arm. For definitions see section “Subgroup definitions”.

### eFigure 15: Mortality -Subgroup analysis 7: Type of device

**
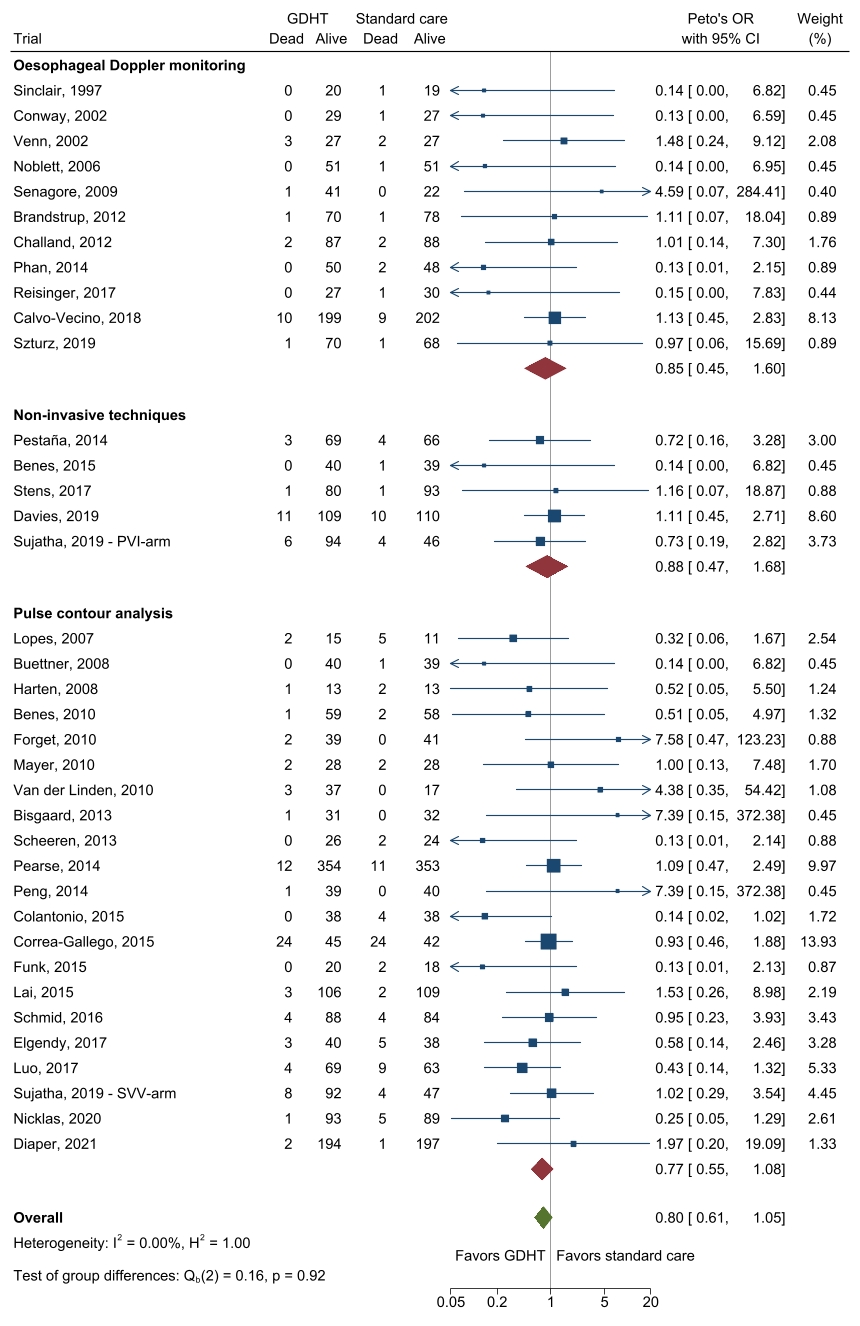
**

Fixed inverse-variance (Peto’s method for odds ratio) meta-analysis of GDHT compared to standard care for overall mortality. Trials are subgrouped according to type of device used to measure the GDHT-target. For definitions see section “Subgroup definitions”.

### eFigure 16: Mortality - Subgroup analysis 8: Type of fluid to reach target


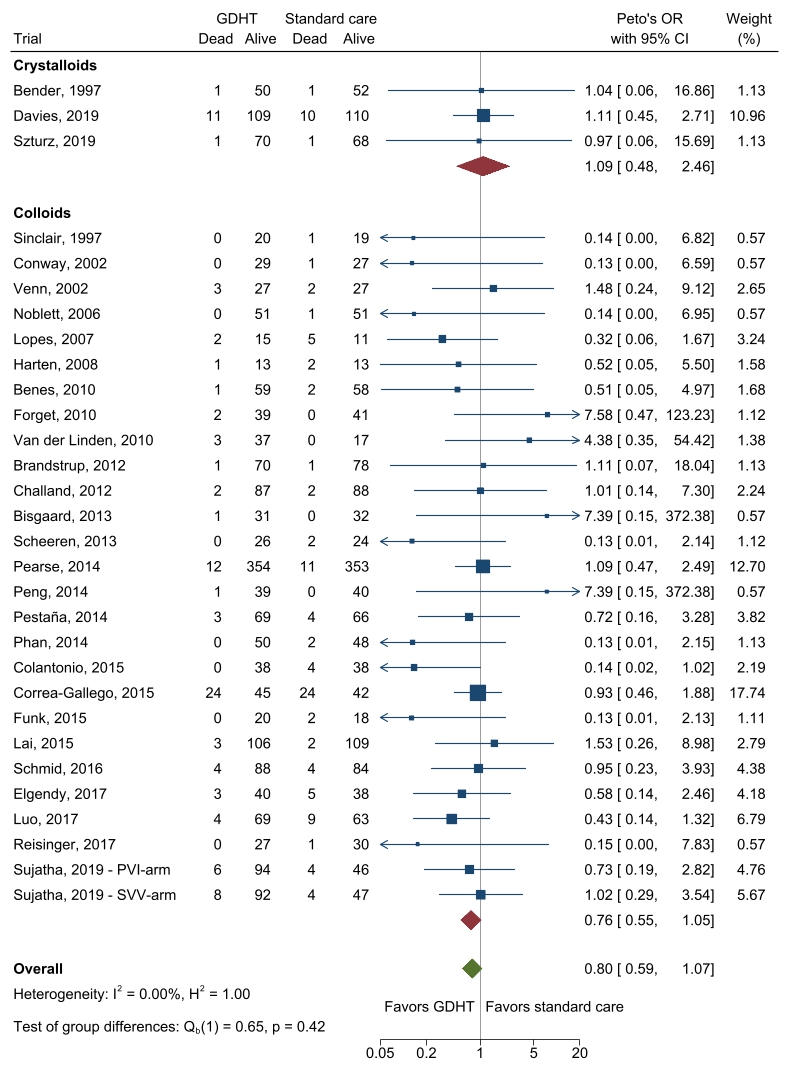


Fixed inverse-variance (Peto’s method for odds ratio) meta-analysis of GDHT compared to standard care for overall mortality. Trials are subgrouped according to type of fluids used to reach GDHT-target. For definitions see section “Subgroup definitions”.

### eFigure 17: Hospital length of stay - Subgroup analysis 1: Risk of surgery

**
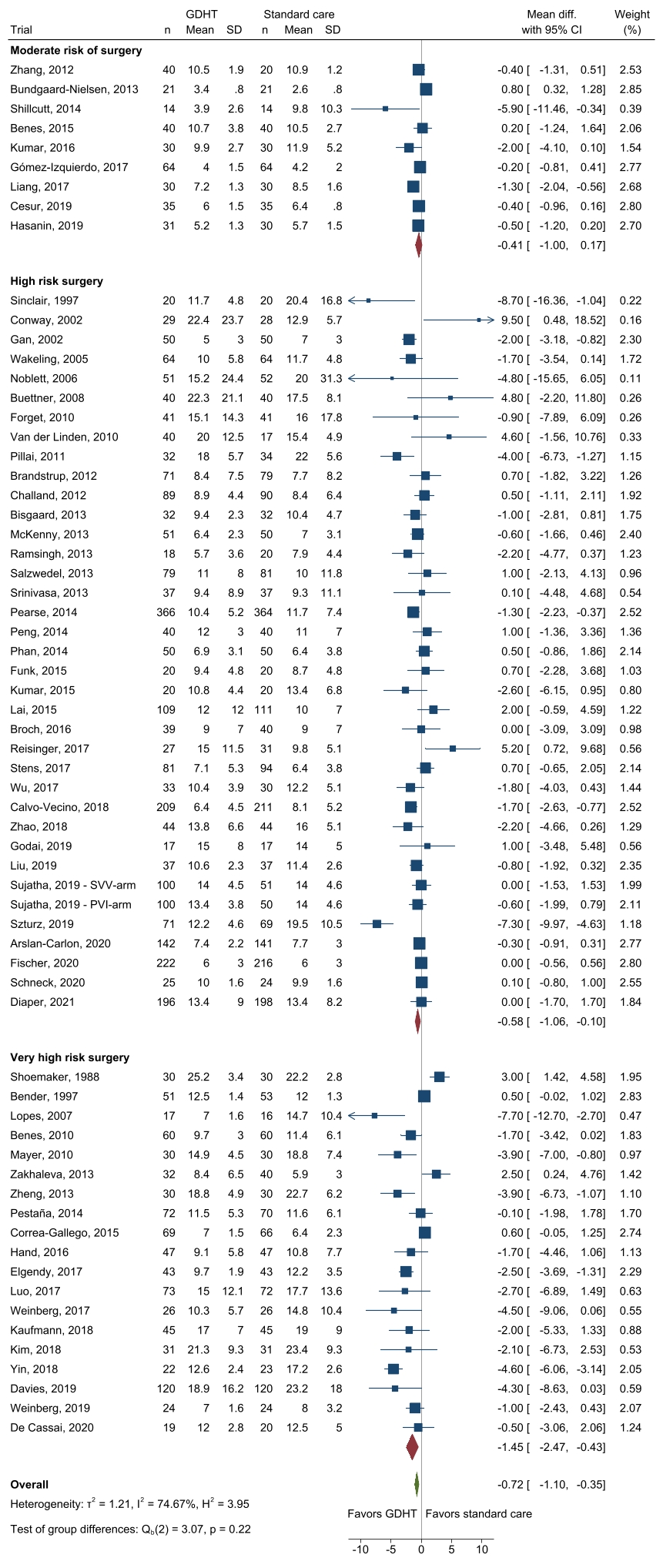
**

Random-effects meta-analysis of GDHT compared to standard care for hospital length of stay in days. Trials are subgrouped according to risk of surgery. For definitions see section “Subgroup definitions”.

### eFigure 18: Hospital length of stay - Subgroup analysis 2: Abdominal surgery vs. non-abdominal surgery

**
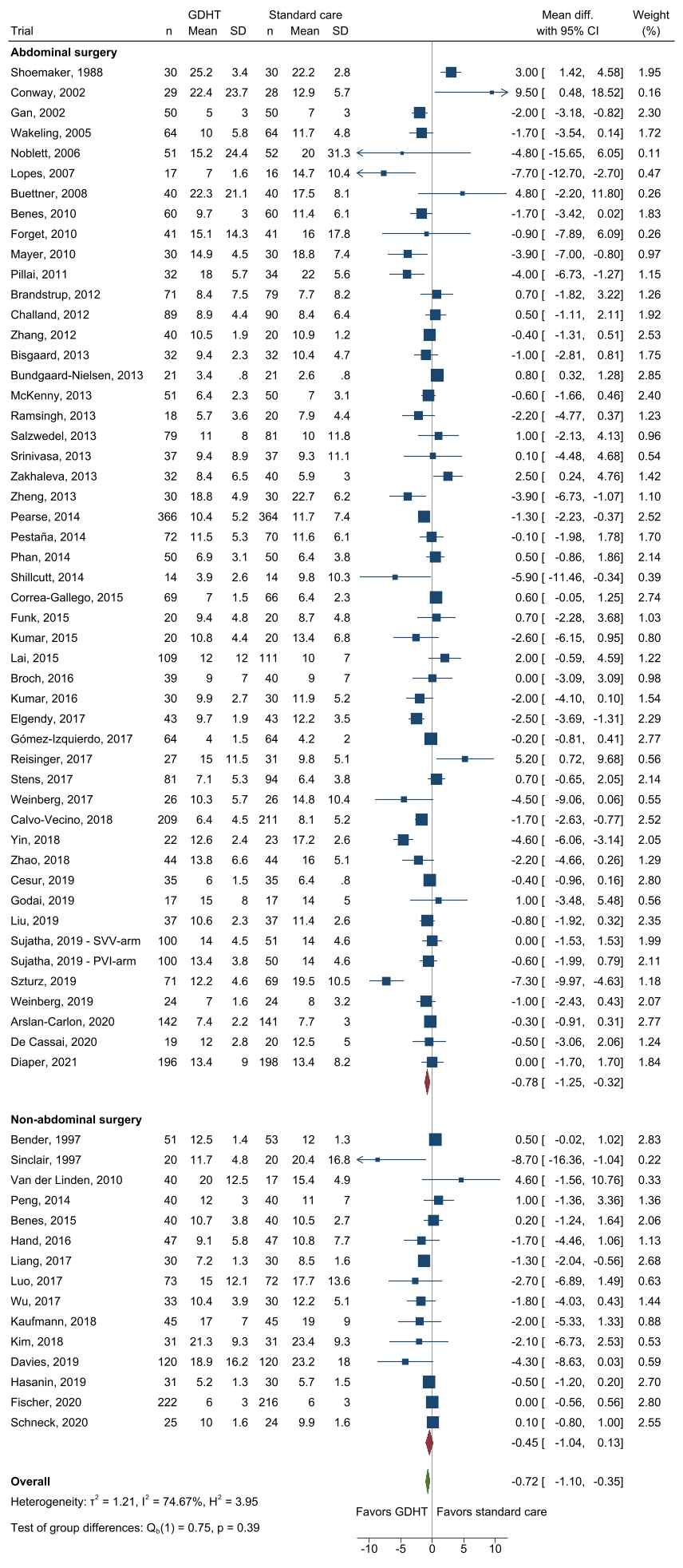
**

Random-effects meta-analysis of GDHT compared to standard care for hospital length of stay in days. Trials are subgrouped as abdominal vs. non-abdominal. For definitions see section “Subgroup definitions”.

### eFigure 19: Hospital length of stay - Subgroup analysis 3: Open surgery vs. laparoscopic surgery


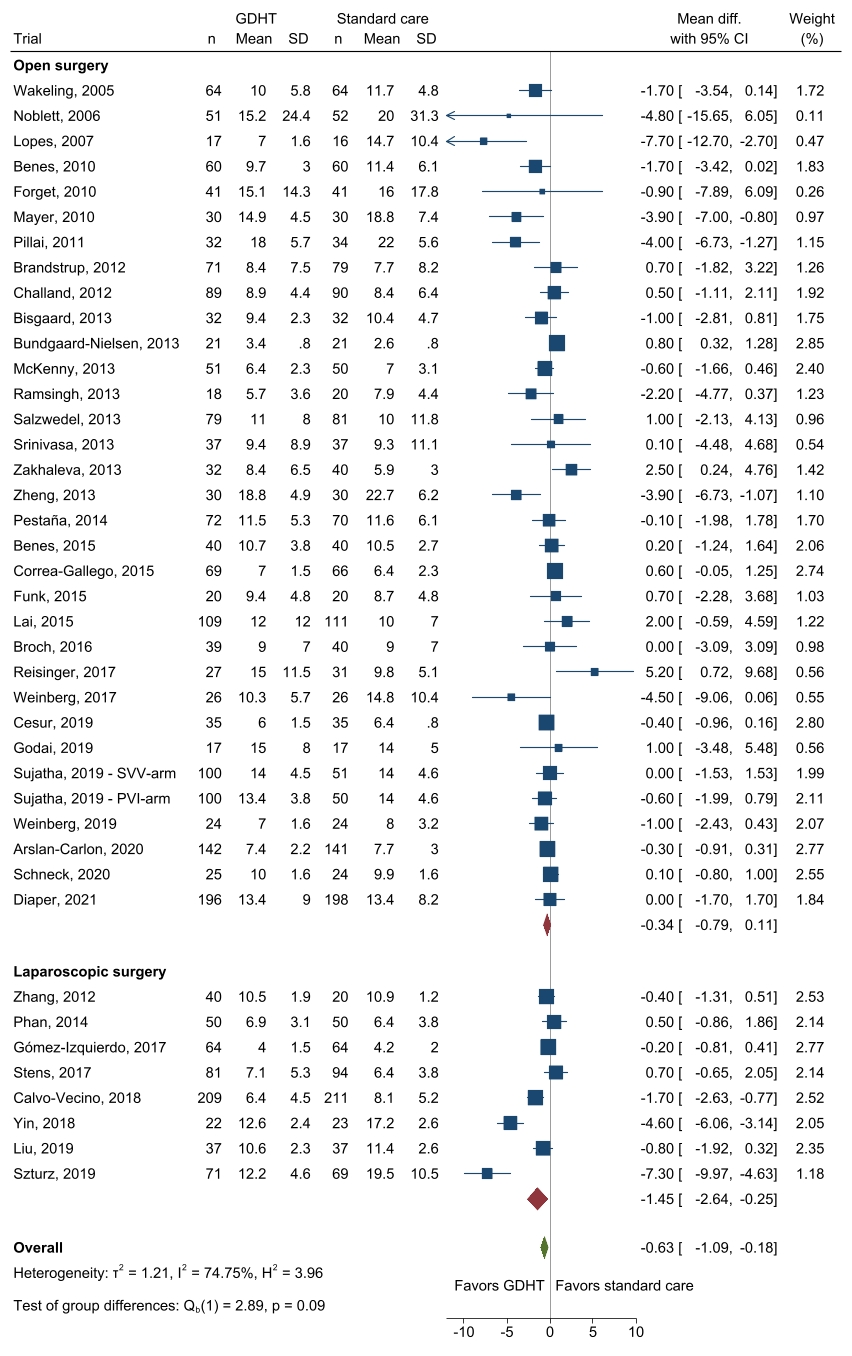


Random-effects meta-analysis of GDHT compared to standard care for hospital length of stay in days. Trials are subgrouped according to the surgical technique as open vs. laparoscopic. For definitions see section “Subgroup definitions”.

### eFigure 20: Hospital length of stay - Subgroup analysis 4: GDHT-targets based on preload variation by the respiratory cycle vs. fluid challenges


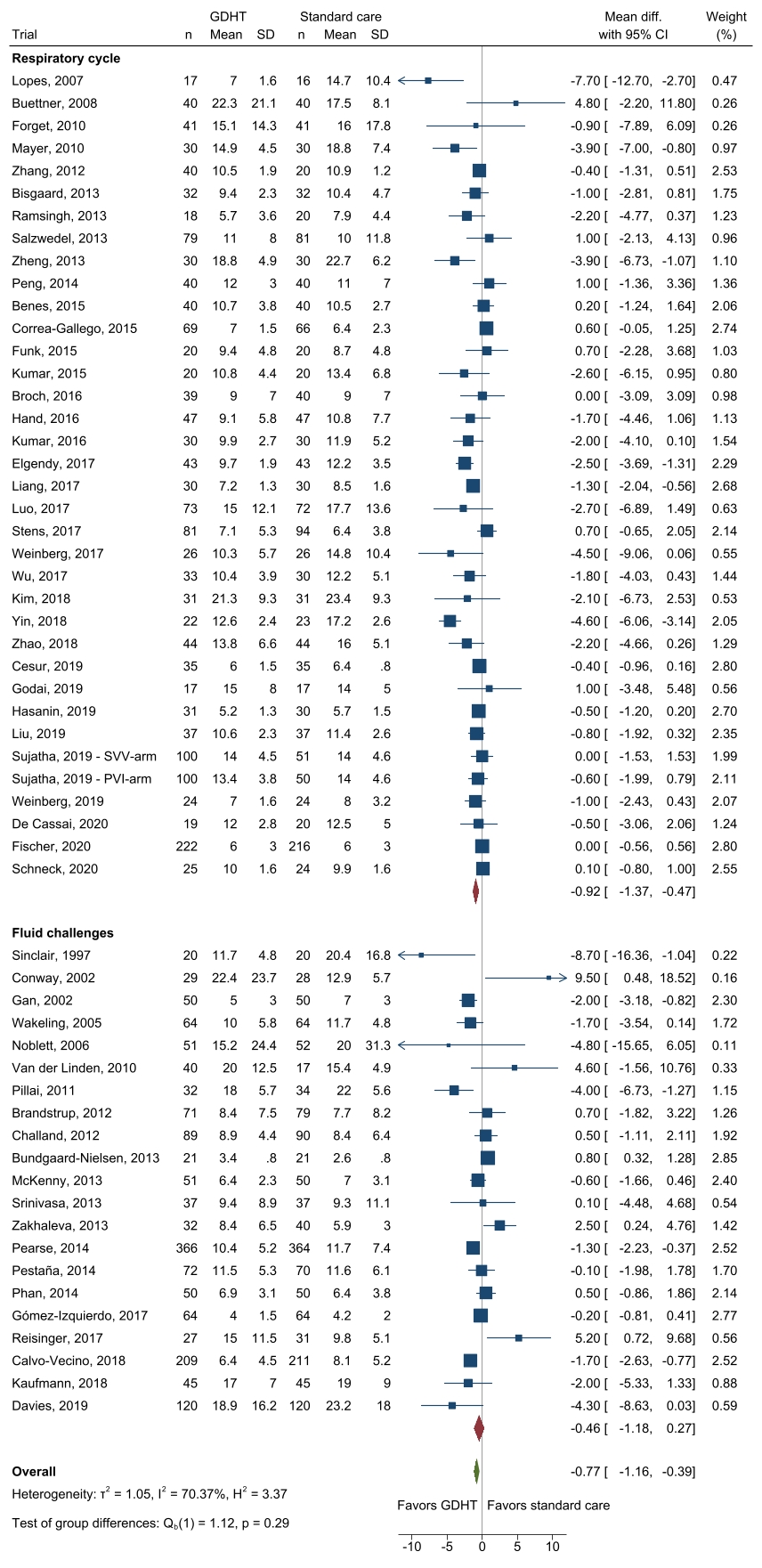


Random-effects meta-analysis of GDHT compared to standard care for hospital length of stay in days. Trials are subgrouped according to concept of preload variation. For definitions see section “Subgroup definitions”.

### eFigure 21: Hospital length of stay - Subgroup analysis 5: GDHT-protocols without vs. with use of inotropes or vasopressors


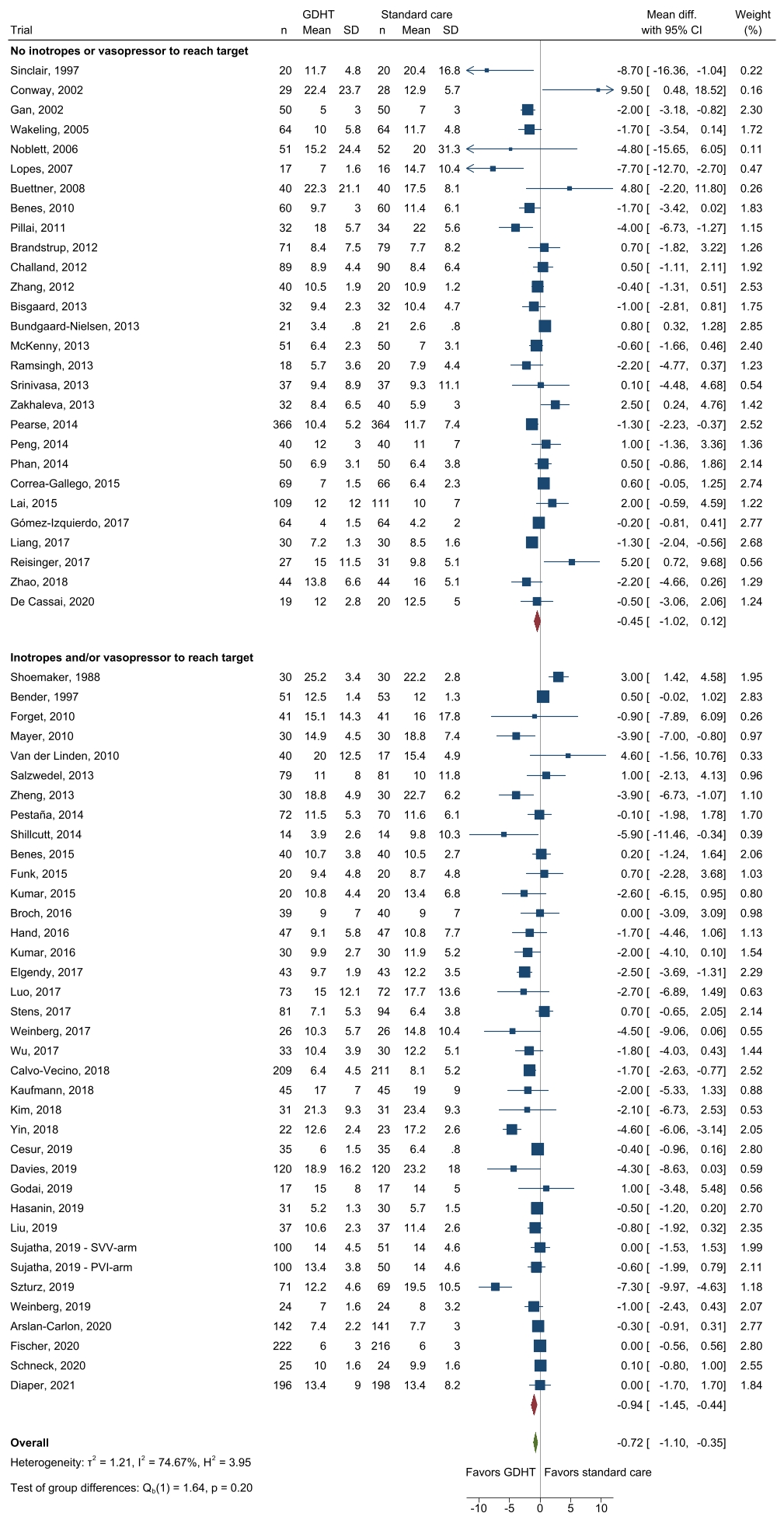


Random-effects meta-analysis of GDHT compared to standard care for hospital length of stay. Trials are subgrouped according to the use of inotropes and/or vasopressors to reach targets.

For definitions see section “Subgroup definitions”.

### eFigure 22: Hospital length of stay - Subgroup analysis 6: GDHT-protocols resulting in less vs. similar vs. more intraoperative fluid amounts than their standard care comparator


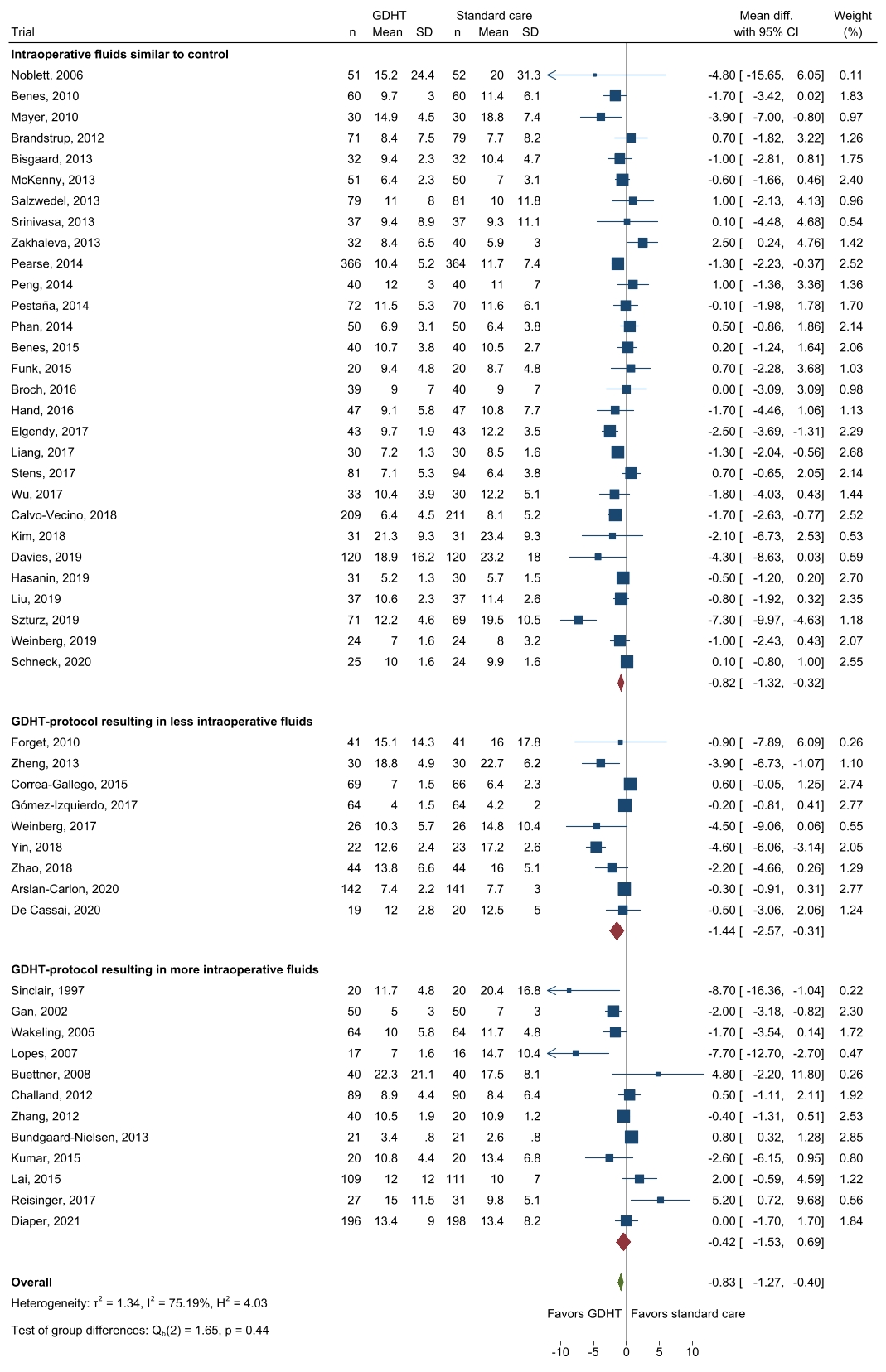


Random-effects meta-analysis of GDHT compared to standard care for hospital length of stay. Trials are subgrouped according to fluid volumes in GDHT-arm vs standard care-arm. For definitions see section “Subgroup definitions”.

### eFigure 23: Hospital length of stay - Subgroup analysis 7: Type of device

**
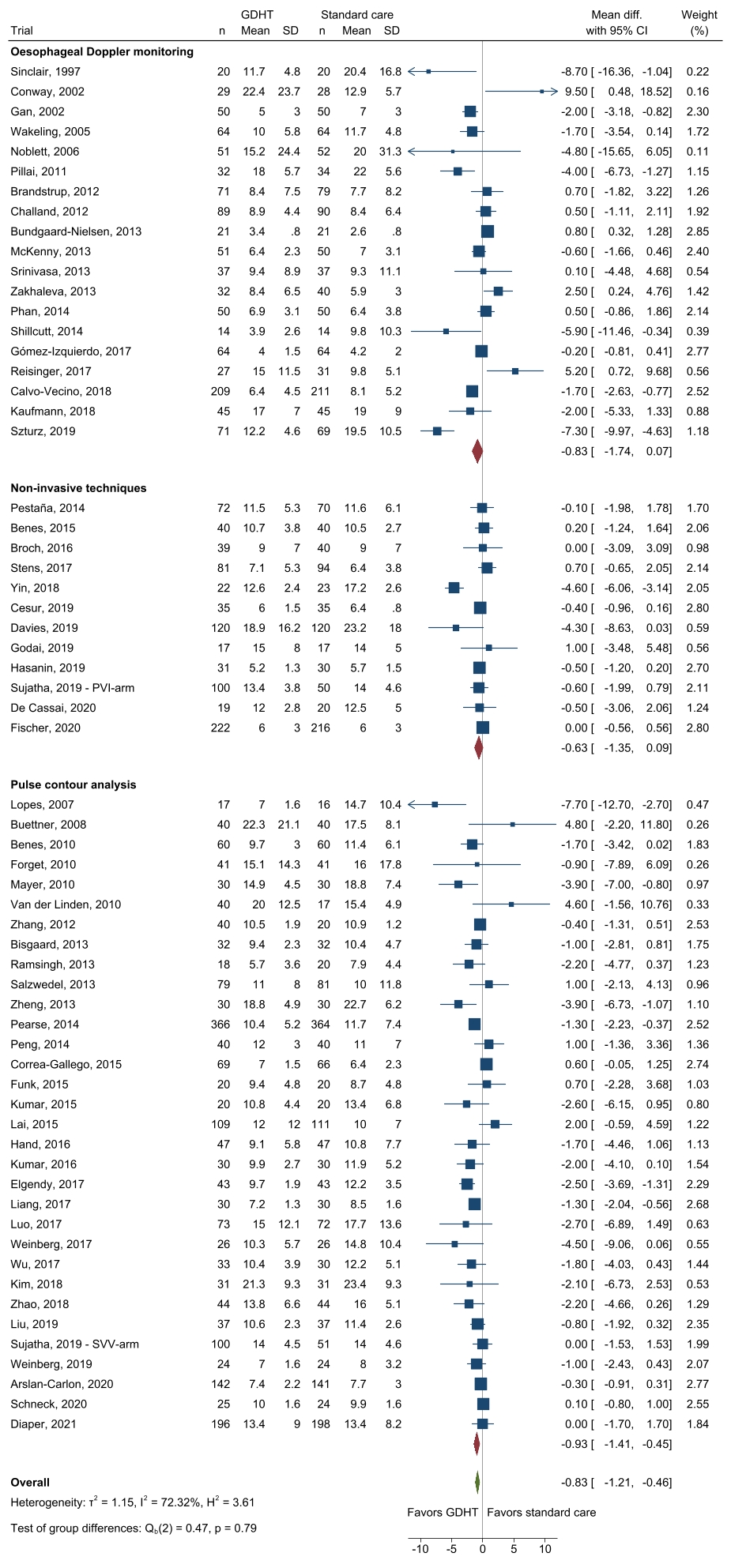
**

Random-effects meta-analysis of GDHT compared to standard care for hospital length of stay. Trials are subgrouped according type of device used to measure target/-s. For definitions see section “Subgroup definitions”.

### eFigure 24: Hospital length of stay - Subgroup analysis 8: Type of fluid to reach target


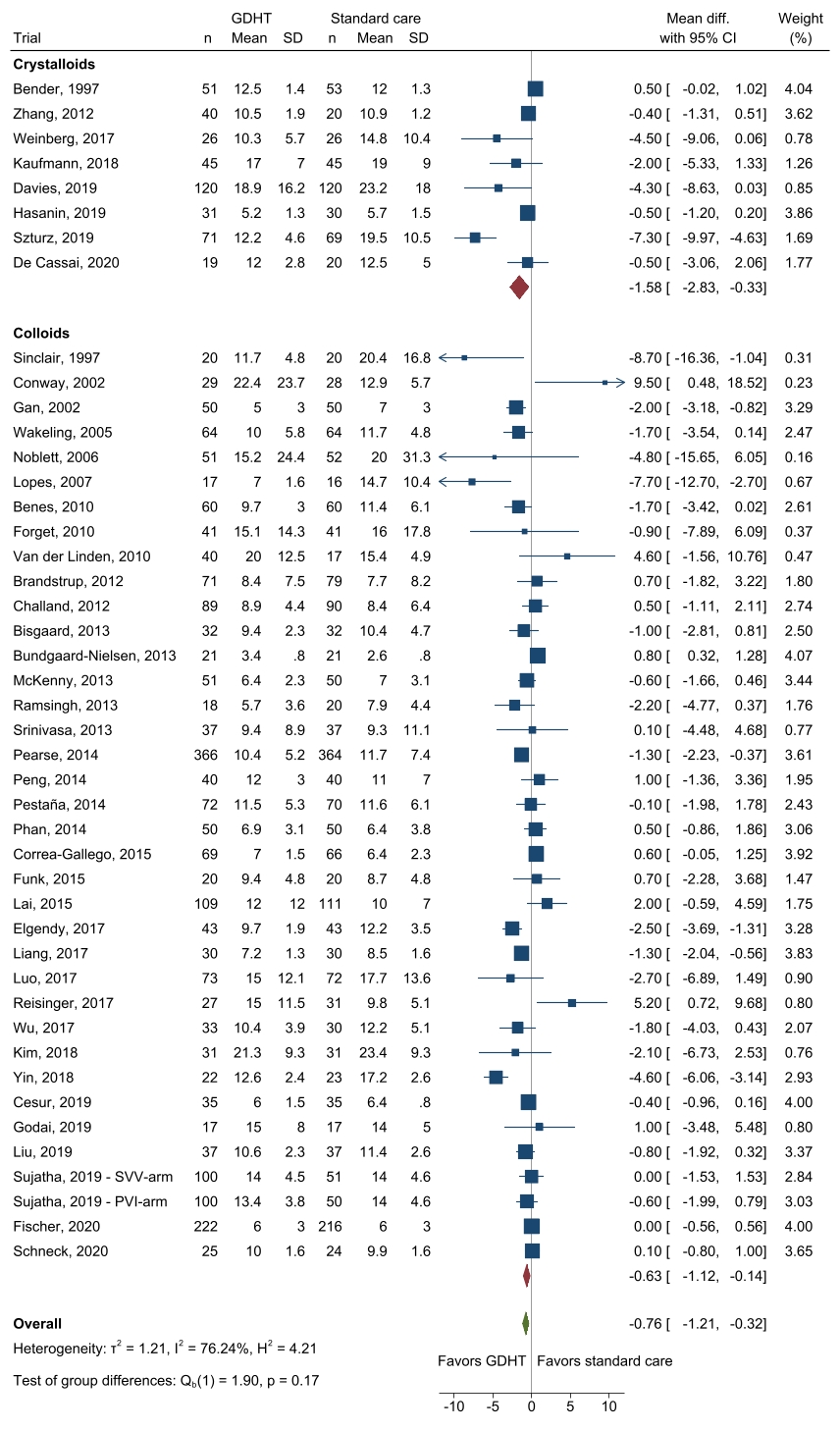


Random-effects meta-analysis of GDHT compared to standard care for hospital length of stay. Trials are subgrouped according type of fluids used to reach target/-s. For definitions see section “Subgroup definitions”.

### eFigure 25: Mortality - Meta-regression 1: Median year of patient inclusion

**
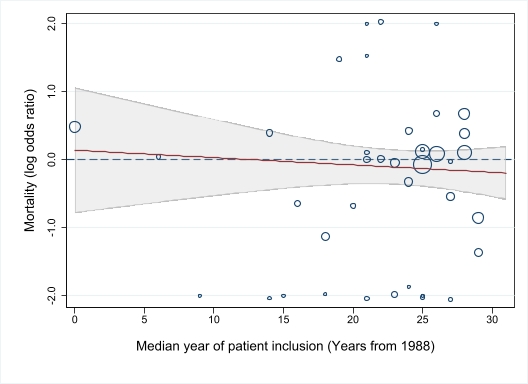
**

The Y-axis represents logarithmic odds ratios of the outcome. The X-axis represents the moderator, median year of patient inclusion in years from 1988. Each circle represents one trial. The size of the circles represents the inverse variance of the study effect sizes. The dashed line represents an odds ratio of 1.0. Values above the dashed line favors standard care and values below the dotted line favors GDHT. The shaded area represents the 95% confidence intervals. Slope, intercept, and P-value are provided in eTable 9.

### eFigure 26: Mortality - Meta-regression 2: Duration of surgery

**
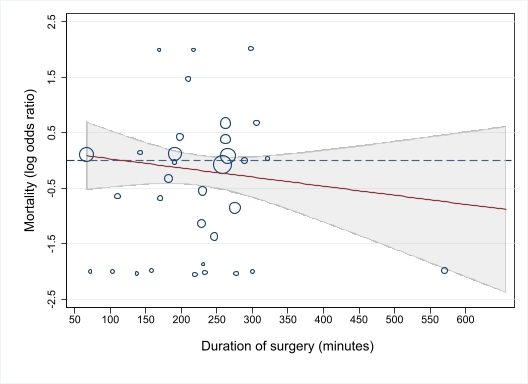
**

The Y-axis represents logarithmic odds ratios of the outcome. The X-axis represents the moderator, duration of surgery in minutes. Each circle represents one trial. The size of the circles represents the inverse variance of the study effect sizes. The dashed line represents an odds ratio of 1.0. Values above the dashed line favours standard care and values below the dotted line favours GDHT. The shaded area represents the 95% confidence intervals. Slope, intercept, and P-value are provided in eTable 9.

### eFigure 27: Mortality - Meta-regression 3: Sample size in trials


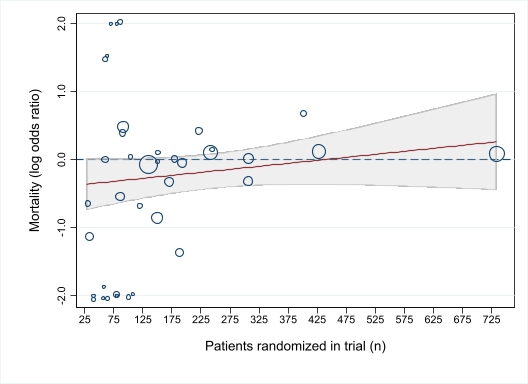


The Y-axis represents logarithmic odds ratios of the outcome. The X-axis represents the moderator, number of total patients randomized (i.e. sample size). Each circle represents one trial. The size of the circles represents the inverse variance of the study effect sizes. The dashed line represents an odds ratio of 1.0. Values above the dashed line favours standard care and values below the dotted line favours GDHT. The shaded area represents the 95% confidence intervals. Studies with LOS > 30 days in the control group are not shown in the bubble plot. Slope, intercept, and P-value are provided in eTable 9.

### eFigure 28: Mortality - Meta-regression 4: Mortality in the control group


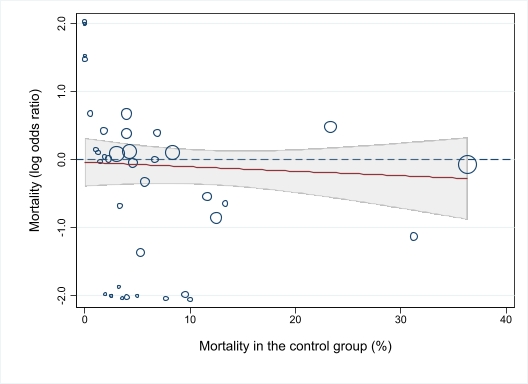


The Y-axis represents logarithmic odds ratios of the outcome. The X-axis represents the moderator, mortality in the control group in percentage. Each circle represents one trial. The size of the circles represents the inverse variance of the study effect sizes. The dashed line represents an odds ratio of 1.0. Values above the dashed line favours standard care and values below the dotted line favours GDHT. The shaded area represents the 95% confidence intervals. Slope, intercept, and P-value are provided in eTable 9.

### eFigure 29: Mortality - Meta-regression 5: Hospital length of stay in the control group


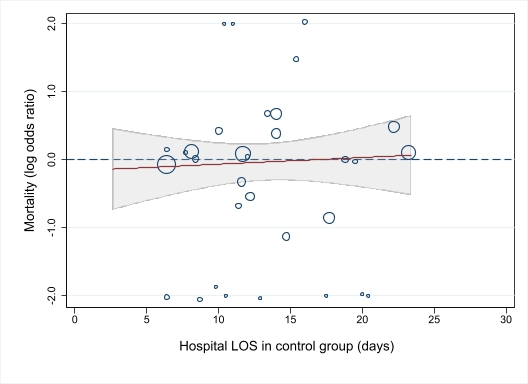


The Y-axis represents logarithmic odds ratios of the outcome. The X-axis represents the moderator, length of stay in the control group in days. Each circle represents one trial. The size of the circles represents the inverse variance of the study effect sizes. The dashed line represents an odds ratio of 1.0. Values above the dashed line favours standard care and values below the dotted line favours GDHT. The shaded area represents the 95% confidence intervals. Studies with LOS > 30 days in the control group are not shown in the bubble plot. Slope, intercept, and P-value are provided in eTable 9.

Abbreviations: **LOS:** Length of stay

### eFigure 30: Hospital length of stay - Meta-regression 1: Median year of patient inclusion

**
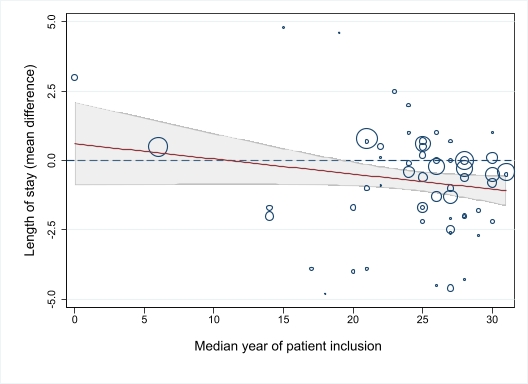
**

**Figure XX. Bubble plot for meta-regression of length of stay and median year of patient inclusion.** The Y-axis represents mean differences of the outcome. The X-axis represents the moderator, median year of patient inclusion in years after 1988. Each circle represents one trial. The size of the circles represents the inverse variance of the study effect sizes. The dashed line represents a mean difference of 0.00. The shaded area represents the 95% confidence intervals. Slope, intercept, and P-value are provided in eTable 9. To improve visualization, outliers (mean differences > 5 or <-5) are not shown in the bubble plot.

### eFigure 31: Hospital length of stay - Meta-regression 2: Duration of surgery


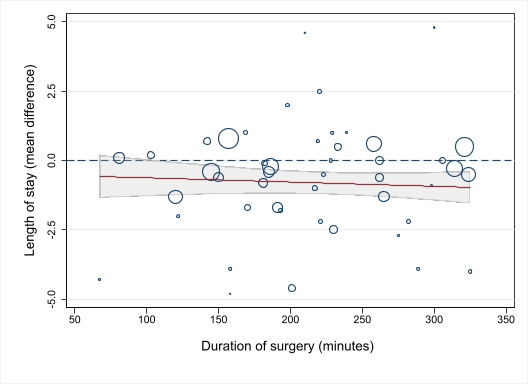


**Figure XX. Bubble plot for meta-regression of length of stay and duration of surgery.** The Y-axis represents mean differences of the outcome. The X-axis represents the moderator, median year of patient inclusion. Each circle represents one trial. The size of the circles represents the inverse variance of the study effect sizes. The dashed line represents a mean difference of 0.00. The shaded area represents the 95% confidence intervals. Slope, intercept, and P-value are provided in eTable 9. To improve visualization, outliers (mean differences > 5 or <-5 and duration of surgery > 350 min) are not shown in the bubble plot.

### eFigure 32: Hospital length of stay - Meta-regression 3: Sample size in trials


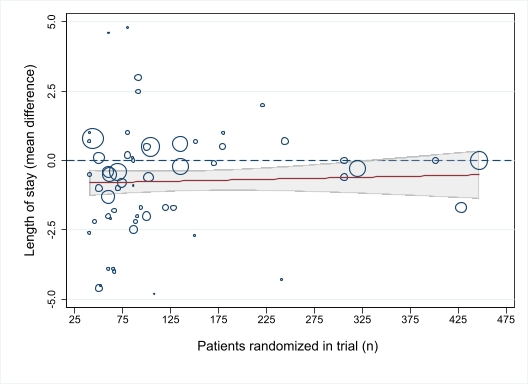


The Y-axis represents logarithmic odds ratios of the outcome. The X-axis represents the moderator, number of total patients randomized (i.e. sample size). Each circle represents one trial. The size of the circles represents the inverse variance of the study effect sizes. The dashed line represents an odds ratio of 1.0. Values above the dashed line favors standard care and values below the dotted line favors GDHT. The shaded area represents the 95% confidence intervals. Studies with LOS > 30 days in the control group are not shown in the bubble plot. Slope, intercept, and P-value are provided in eTable 9.

### eFigure 33: Hospital length of stay - Meta-regression 4: Mortality in the control group


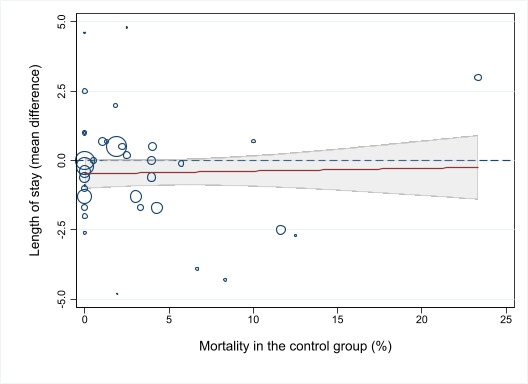


The Y-axis represents mean differences of the outcome. The X-axis represents the moderator, median year of patient inclusion. Each circle represents one trial. The size of the circles represents the inverse variance of the study effect sizes. The dashed line represents a mean difference of 0.00. The shaded area represents the 95% confidence intervals. Slope, intercept, and P-value are provided in eTable 9. To improve visualization, outliers (mean differences > 5 or < -5 and mortality in control group > 25%) are not shown in the bubble plot.

### eFigure 34: Hospital length of stay - Meta-regression 5: Hospital length of stay in the control group


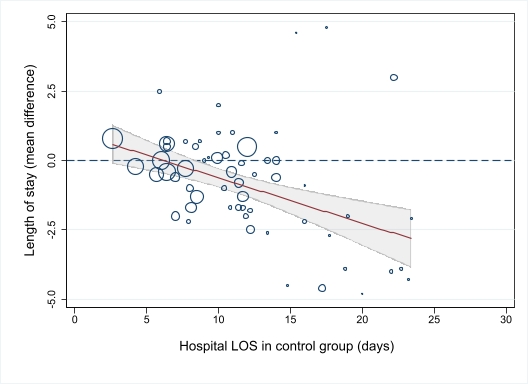


The Y-axis represents mean differences of the outcome. The X-axis represents the moderator, median year of patient inclusion. Each circle represents one trial. The size of the circles represents the inverse variance of the study effect sizes. The dashed line represents a mean difference of 0.00. The shaded area represents the 95% confidence intervals. Slope, intercept, and P-value are provided in eTable 9. To improve visualization, outliers (mean differences >5 or <-5 and length of stay in control group > 25 days) are not shown in the bubble plot.

The mean difference in length of stay increased with increasing length of stay in the standard care group.

### eFigure 35: Mortality - Funnel plot

**
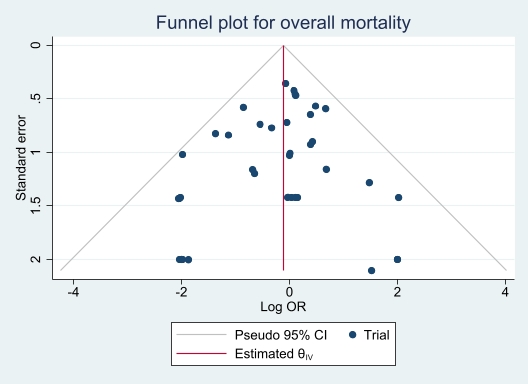
**

Funnel plot to assess the risk of publication bias for the outcome overall mortality. The x-axis represents the logarithmic odds ratio (i.e., the effect size) and the y-axis the standard error (i.e., a measure of precision and trial size). The red vertical line represents the overall effect size from the meta-analysis. Every blue dot represents one clinical trial.

### eFigure 36: Hospital length of stay - Funnel plot


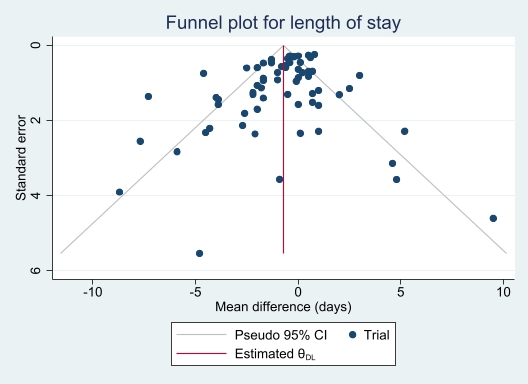


Funnel plot to assess the risk of publication bias. The x-axis represents the mean differences in length of stay (GDHT vs. standard care group) (i.e., the effect size) and the y-axis the standard error (i.e., a measure of precision and trial size). The red vertical line represents the overall effect size from the meta-analysis. Every blue dot represents one clinical trial.

## Pneumonia

### eFigure 37: Pneumonia - Primary analysis with abdominal surgery vs. non-abdominal surgery

**
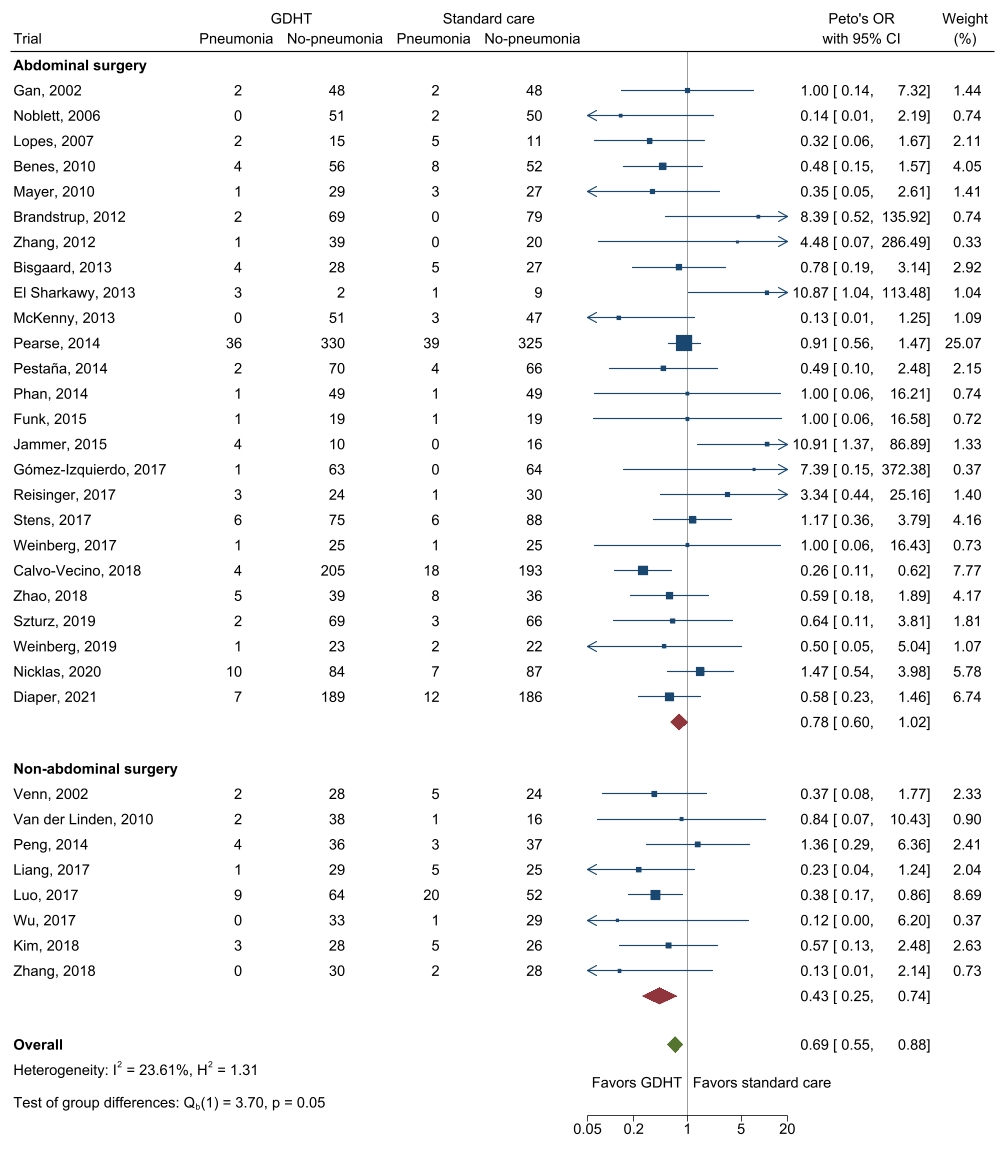
**

Fixed inverse-variance (Peto’s method for odds ratio) meta-analysis of GDHT compared to standard care for pneumonia. Trials are subgrouped according to surgery type. For definitions see section ”Outcomes: Definitions, data synthesis, and sensitivity analyses”.

### eFigure 38: Pneumonia - Sensitivity analysis 1: Excluding trials with high risk of bias


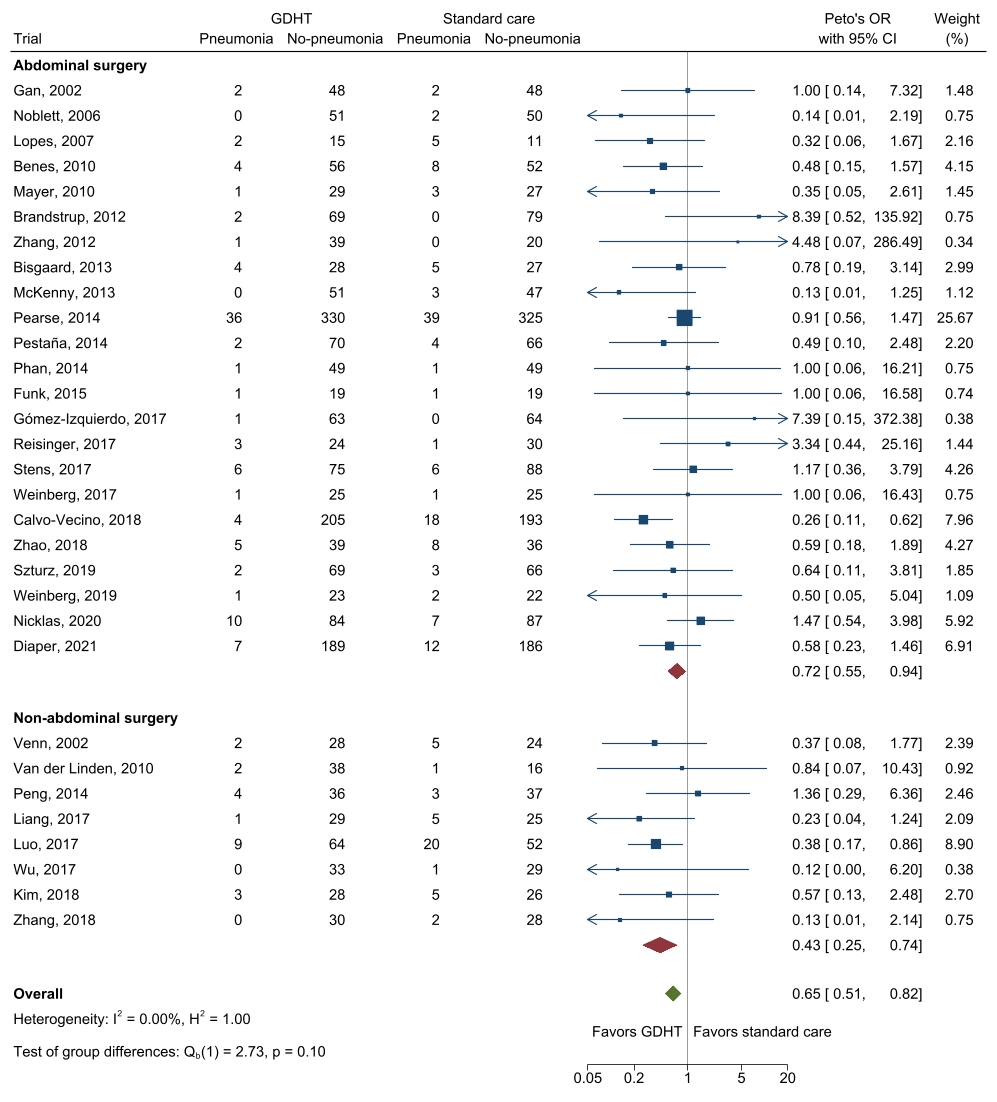


Fixed inverse-variance (Peto’s method for odds ratio) meta-analysis of GDHT compared to standard care for pneumonia without high risk of bias-trials. Trials with high-risk of bias are not included. Trials are subgrouped according to surgery type. For definitions see section ”Outcomes: Definitions, data synthesis, and sensitivity analyses”.

### eFigure 39: Pneumonia - Sensitivity analysis 2: Excluding trials without an outcome definition


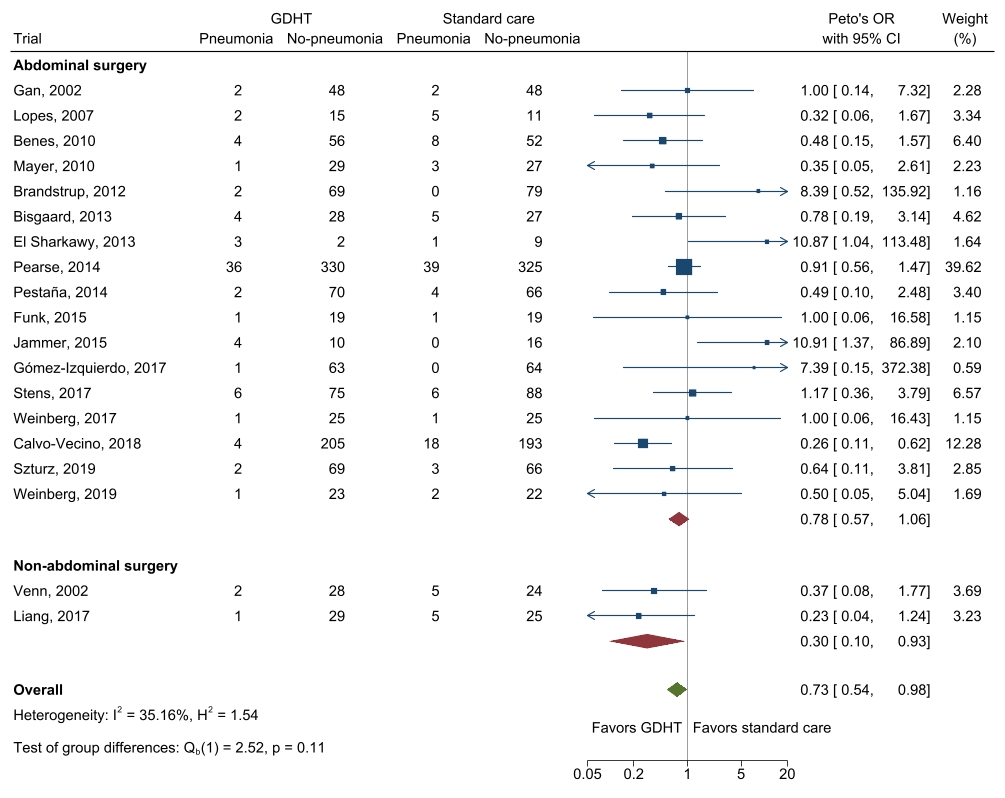


Fixed inverse-variance (Peto’s method for odds ratio) meta-analysis of GDHT compared to standard care for pneumonia. Trials using a clear definition of pneumonia are subgrouped according to surgery type. For definitions see section ”Outcomes: Definitions, data synthesis, and sensitivity analyses”.

All trials that reported an outcome definition had one or more events in either group.

### eFigure 40: Pneumonia - Sensitivity analysis 3: Only including trials with established definitions

**
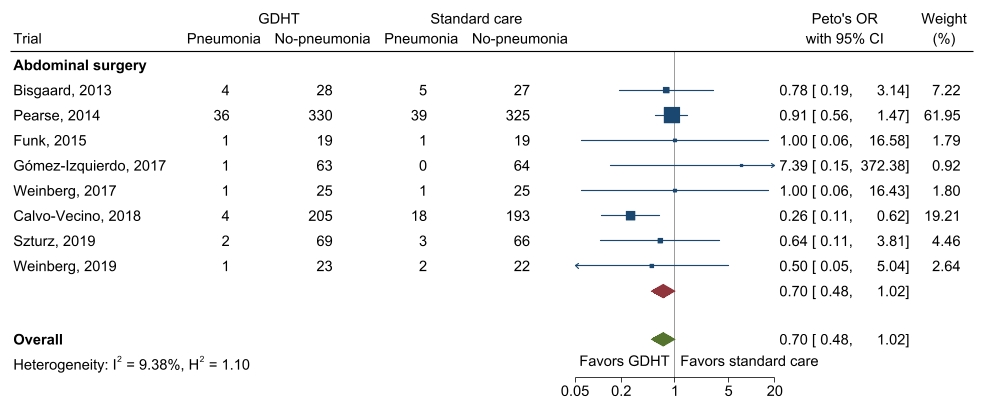
**

Fixed inverse-variance (Peto’s method for odds ratio) meta-analysis of GDHT compared to standard care for pneumonia. Trials using EPCO 2015^2^/CDC 2008^3^/NSQIP 2014^5^/ATS 2005^9^-definitions of pneumonia are subgrouped according to surgery type. For definitions see section ”Outcomes: Definitions, data synthesis, and sensitivity analyses”.

## Pulmonary oedema

### eFigure 41: Pulmonary oedema - Primary analysis


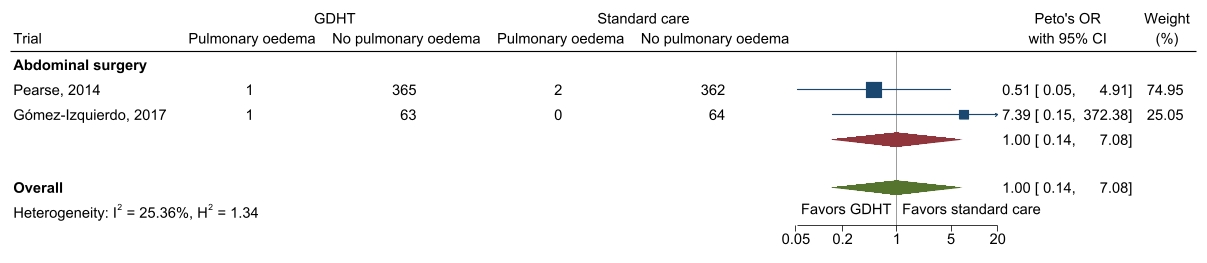


Fixed inverse-variance (Peto’s method for odds ratio) meta-analysis of GDHT compared to standard care for pulmonary oedema.

### eFigure 42: Pulmonary oedema - Sensitivity analysis 1: All trials reporting the outcome


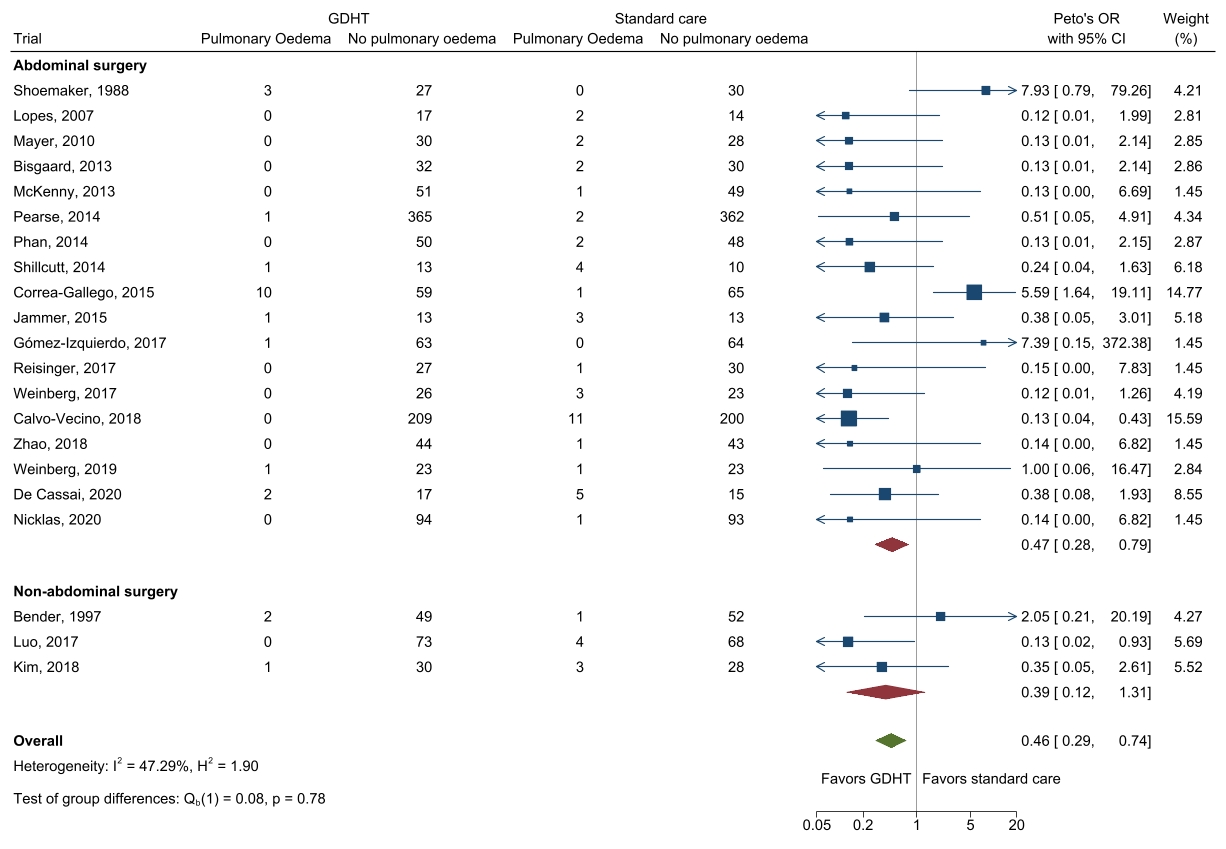


Fixed inverse-variance (Peto’s method for odds ratio) meta-analysis of GDHT compared to standard care for pulmonary oedema for all trials reporting the outcome. For definitions see section ”Outcomes: Definitions, data synthesis, and sensitivity analyses”.

## Pulmonary embolism

### eFigure 43: Pulmonary embolism - Primary analysis with abdominal surgery vs. non-abdominal surgery


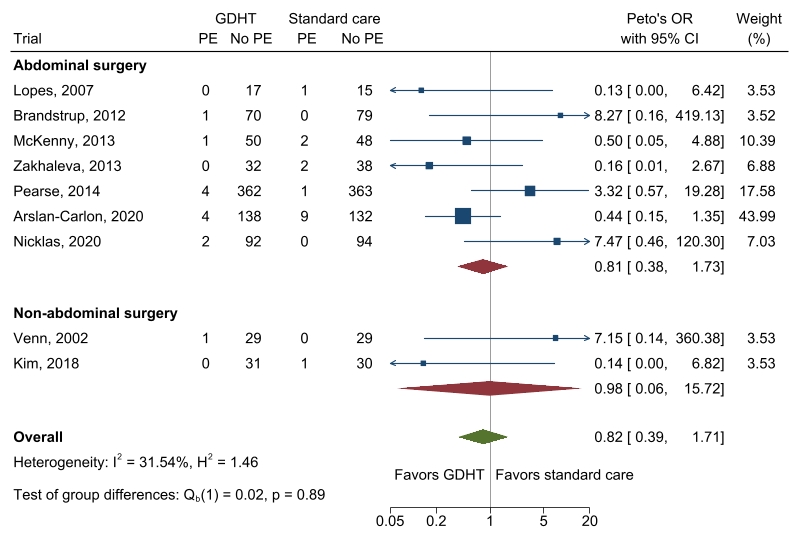


Fixed inverse-variance (Peto’s method for odds ratio) meta-analysis of GDHT compared to standard care for pulmonary embolism. Trials are subgrouped according to surgery type. For definitions see section ”Outcomes: Definitions, data synthesis, and sensitivity analyses”.

Abbreviations: PE: Pulmonary embolism

### eFigure 44: Pulmonary embolism - Sensitivity analysis 1: Excluding trials without an outcome definition


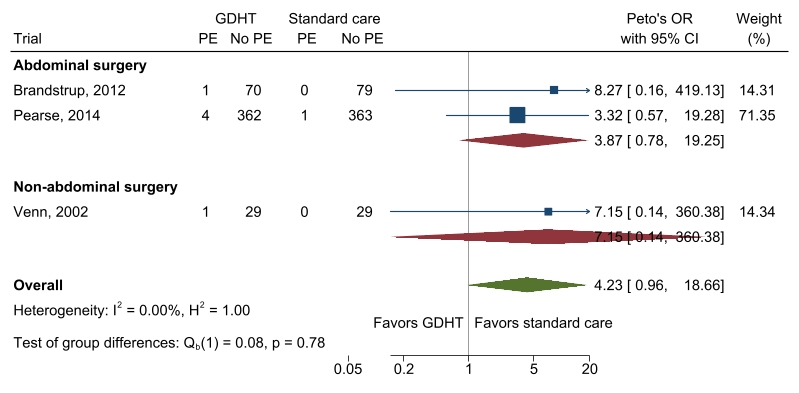


Fixed inverse-variance (Peto’s method for odds ratio) meta-analysis of GDHT compared to standard care for pulmonary embolism. Trials are subgrouped according to surgery type. For definitions see section ”Outcomes: Definitions, data synthesis, and sensitivity analyses”.

Six trials that reported an outcome definition had no events in either group and were therefore excluded from the analysis.

Abbreviations: **PE:** Pulmonary embolism

## Acute respiratory distress syndrome

### eFigure 45: Acute respiratory distress syndrome - Primary analysis


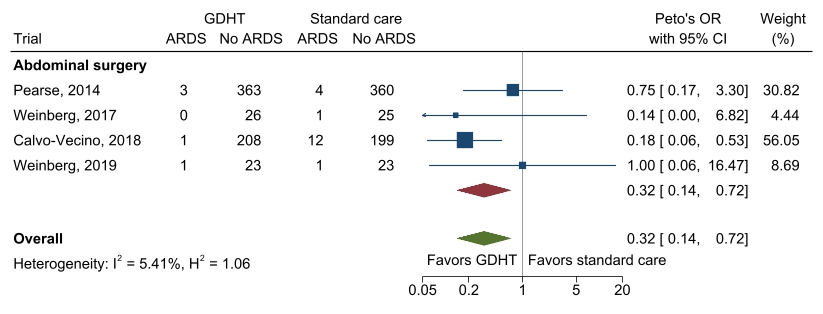


Fixed inverse-variance (Peto’s method for odds ratio) meta-analysis of GDHT compared to standard care for acute respiratory distress syndrome in trials using EPCO 2015^2^ / “Berlin”-definitions^10^ of outcome. Trials are subgrouped according to surgery type. For definitions see section ”Outcomes: Definitions, data synthesis, and sensitivity analyses”.

Abbreviations: **ARDS:** Acute respiratory distress syndrome

### eFigure 46: Acute respiratory distress syndrome – Sensitivity analysis 1: All trials reporting the outcome


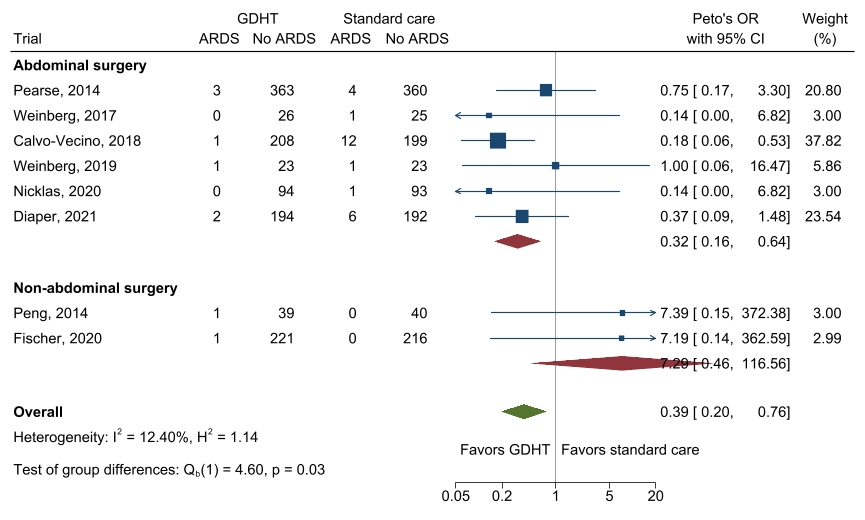


Fixed inverse-variance (Peto’s method for odds ratio) meta-analysis of GDHT compared to standard care for acute respiratory distress syndrome in trials using EPCO 2015^2^ / “Berlin”-definitions^10^ of the outcome. Trials are subgrouped according to surgery type. For definitions see section ”Outcomes: Definitions, data synthesis, and sensitivity analyses”.

Abbreviations: **ARDS:** Acute respiratory distress syndrome

## Myocardial infarction

### eFigure 47: Myocardial infarction - Primary analysis with abdominal surgery vs. non-abdominal surgery


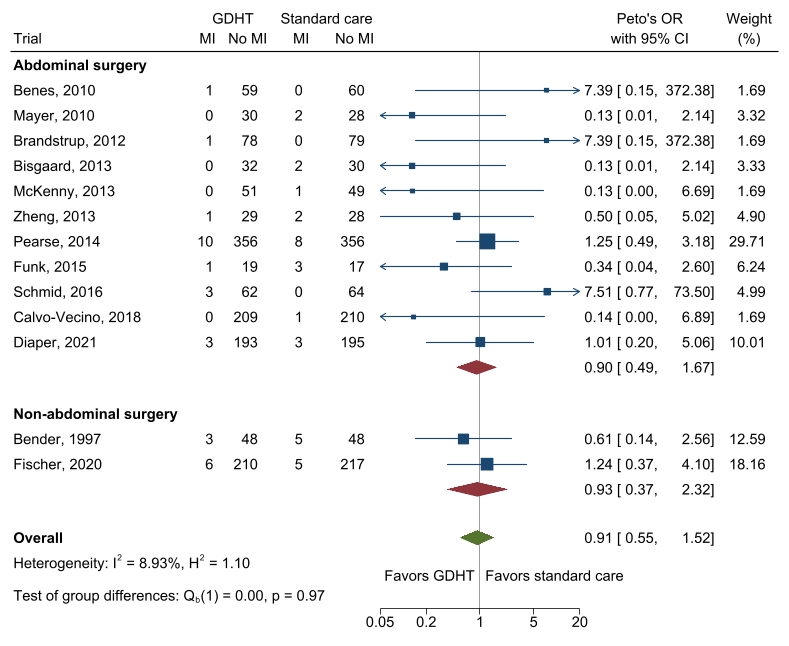


Fixed inverse-variance (Peto’s method for odds ratio) meta-analysis of GDHT compared to standard care for myocardial infarction. Trials are subgrouped according to surgery type. For definitions see section ”Outcomes: Definitions, data synthesis, and sensitivity analyses”.

Abbreviations: **MI:** Myocardial infarction

### eFigure 48: Myocardial infarction - Sensitivity analysis 1: Excluding trials without an outcome definition


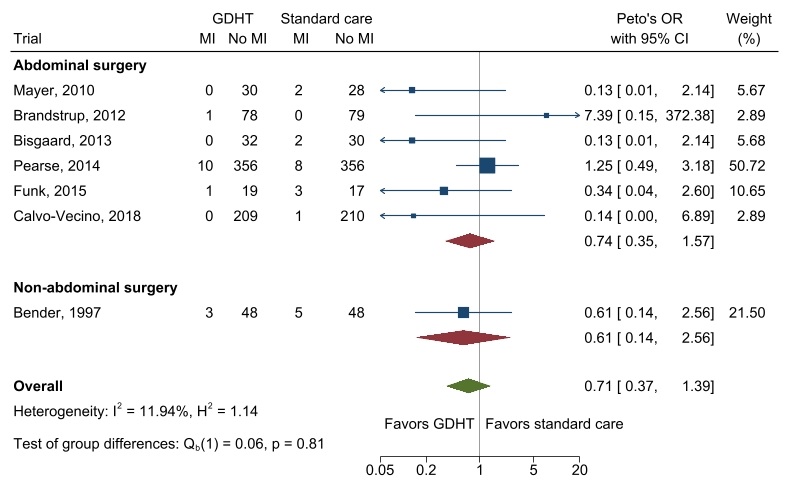


Fixed inverse-variance (Peto’s method for odds ratio) meta-analysis of GDHT compared to standard care for myocardial infarction. Trials with clear outcome definitions are subgrouped according to surgery type. For definitions see section ”Outcomes: Definitions, data synthesis, and sensitivity analyses”.

Three trials that reported an outcome definition had no events in either group and were therefore excluded from the analysis.

Abbreviations: **MI:** Myocardial infarction

## Arrhythmia

### eFigure 49: Arrhythmia - Primary analysis


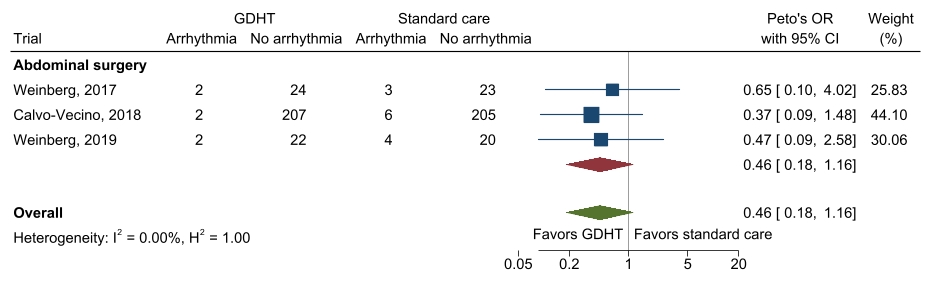


Fixed inverse-variance (Peto’s method for odds ratio) meta-analysis of GDHT compared to standard care for arrhythmia in trials using EPCO2015-definitions^2^. Trials are subgrouped according to surgery type. For definitions see section ”Outcomes: Definitions, data synthesis, and sensitivity analyses”.

### eFigure 50: Arrhythmia – Sensitivity analysis 1: Definition “requiring intervention”


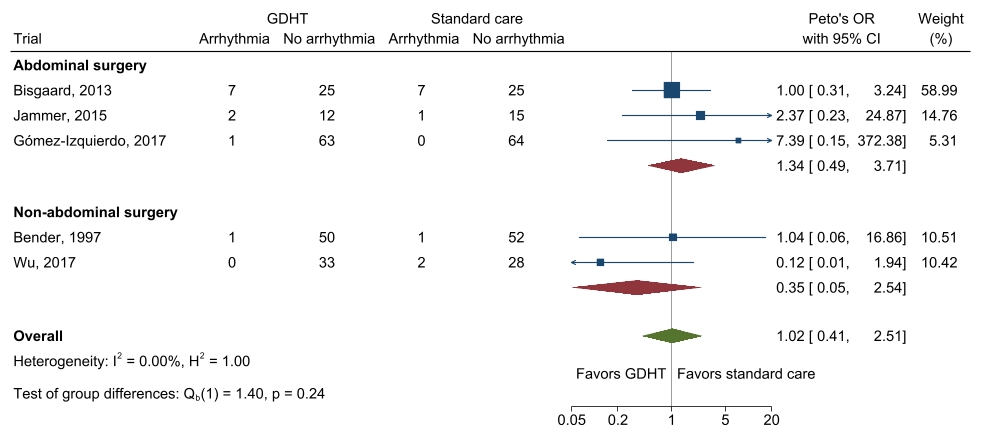


Fixed inverse-variance (Peto’s method for odds ratio) meta-analysis of GDHT compared to standard care for arrhythmia. Trials defined the outcome by a requirement of either medical or defibrillation intervention. Trials are subgrouped according to surgery type. For definitions see section ”Outcomes: Definitions, data synthesis, and sensitivity analyses”.

### eFigure 51: Arrhythmia - Sensitivity analysis 2: Definition “requiring intervention” excluding trials with high risk of bias


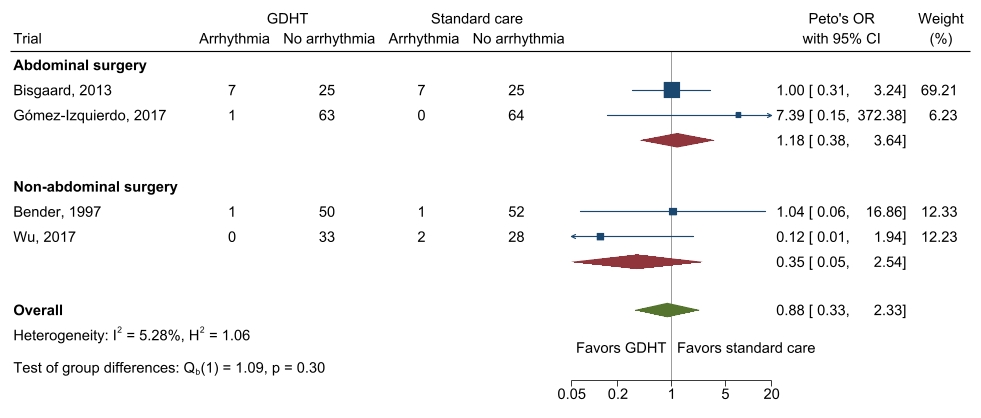


Fixed inverse-variance (Peto’s method for odds ratio) meta-analysis of GDHT compared to standard care for arrhythmia without high risk of bias-trials. Trials defined the outcome by a requirement of either medical or defibrillation intervention. Trials are subgrouped according to surgery type. For definitions see section ”Outcomes: Definitions, data synthesis, and sensitivity analyses”.

### eFigure 52: Arrhythmia - Sensitivity analysis 3: All trials reporting the outcome


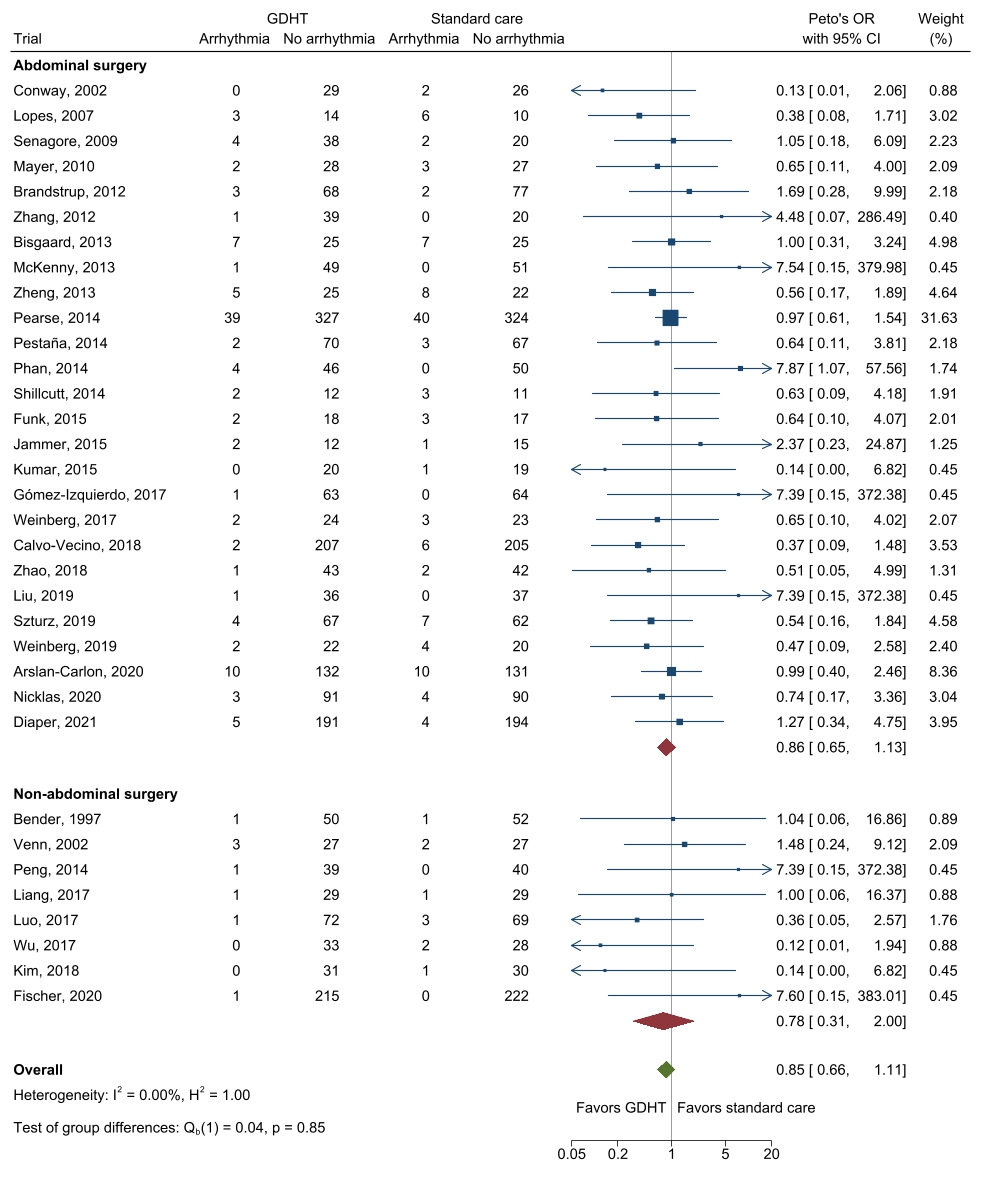


Fixed inverse-variance (Peto’s method for odds ratio) meta-analysis of GDHT compared to standard care for arrhythmia. All trials reporting the outcome are included. For definitions see section ”Outcomes: Definitions, data synthesis, and sensitivity analyses”.

## Acute kidney injury

### eFigure 53: Acute kidney injury - Primary analysis with abdominal surgery vs. non-abdominal surgery


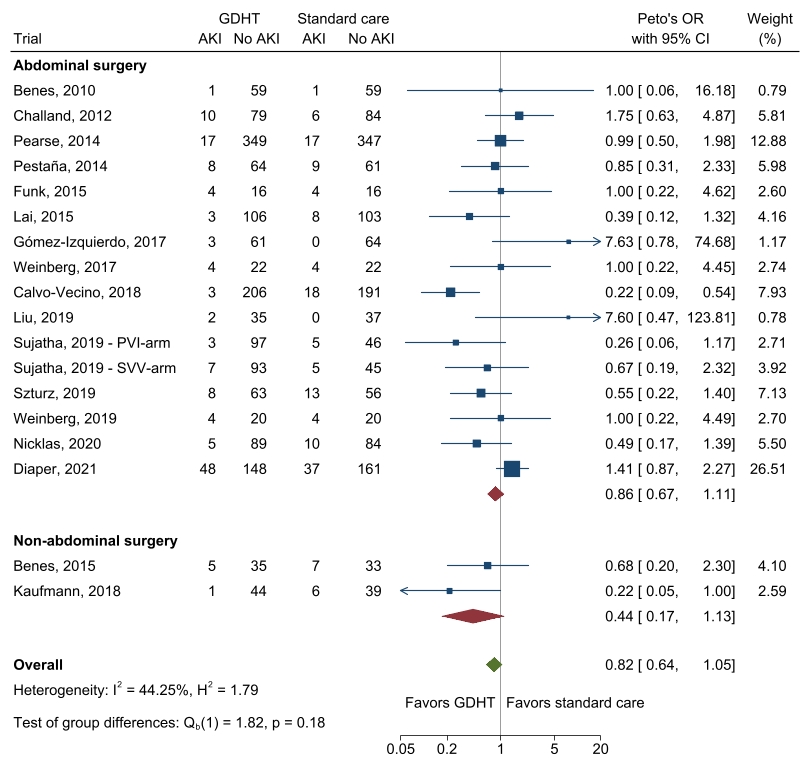


Fixed inverse-variance (Peto’s method for odds ratio) meta-analysis of GDHT compared to standard care for AKI. Included trials defined AKI by a postoperative increase in creatinine by at least 150%. Trials are subgrouped according to surgery type. For definitions see section ”Outcomes: Definitions, data synthesis, and sensitivity analyses”.

Abbreviations: **AKI:** Acute kidney injury

### eFigure 54: Acute kidney injury - Sensitivity analysis 1: All trials reporting the outcome


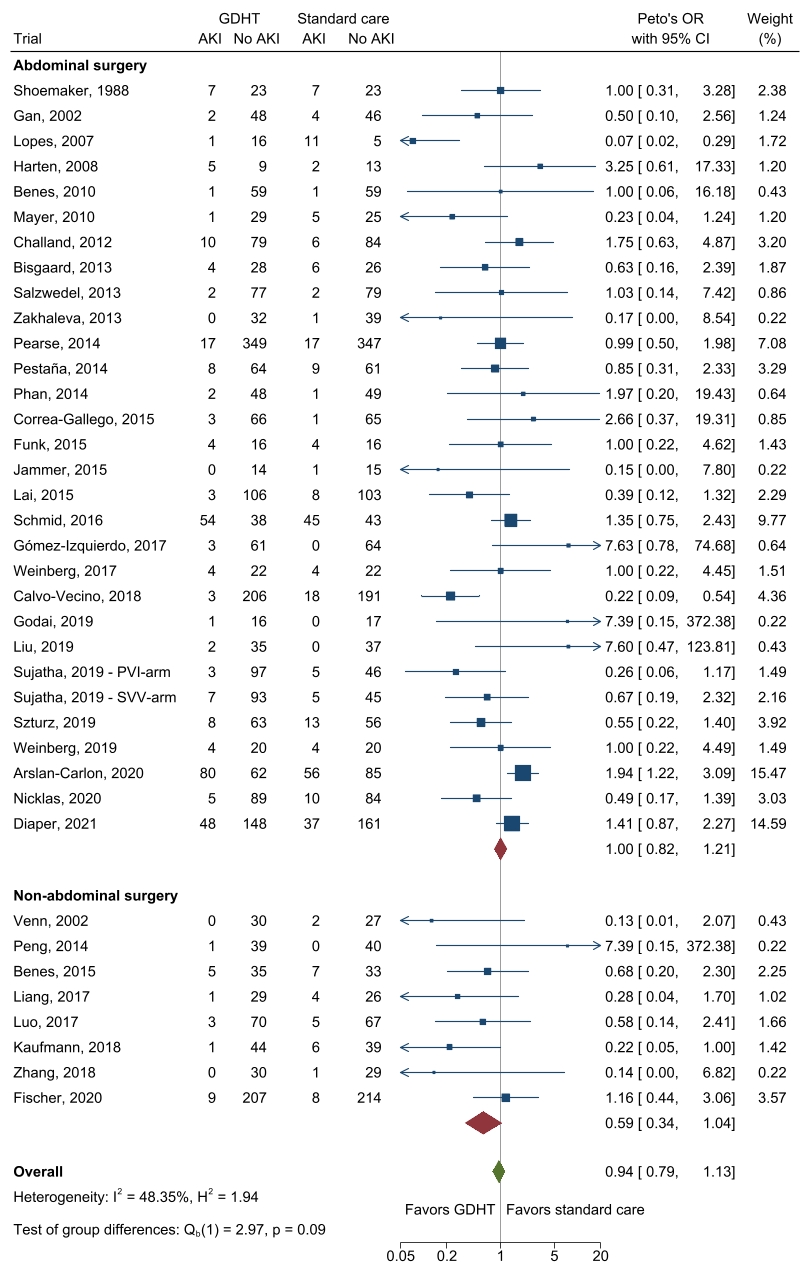


Fixed inverse-variance (Peto’s method for odds ratio) meta-analysis of GDHT compared to standard care for AKI. All trials that reported the outcome are included. Trials are subgrouped according to surgery type. For definitions see section ”Outcomes: Definitions, data synthesis, and sensitivity analyses”.

Abbreviations: **AKI:** Acute kidney injury

## Surgical site infection

### eFigure 55: Surgical site infection - Primary analysis with abdominal surgery vs. non-abdominal surgery


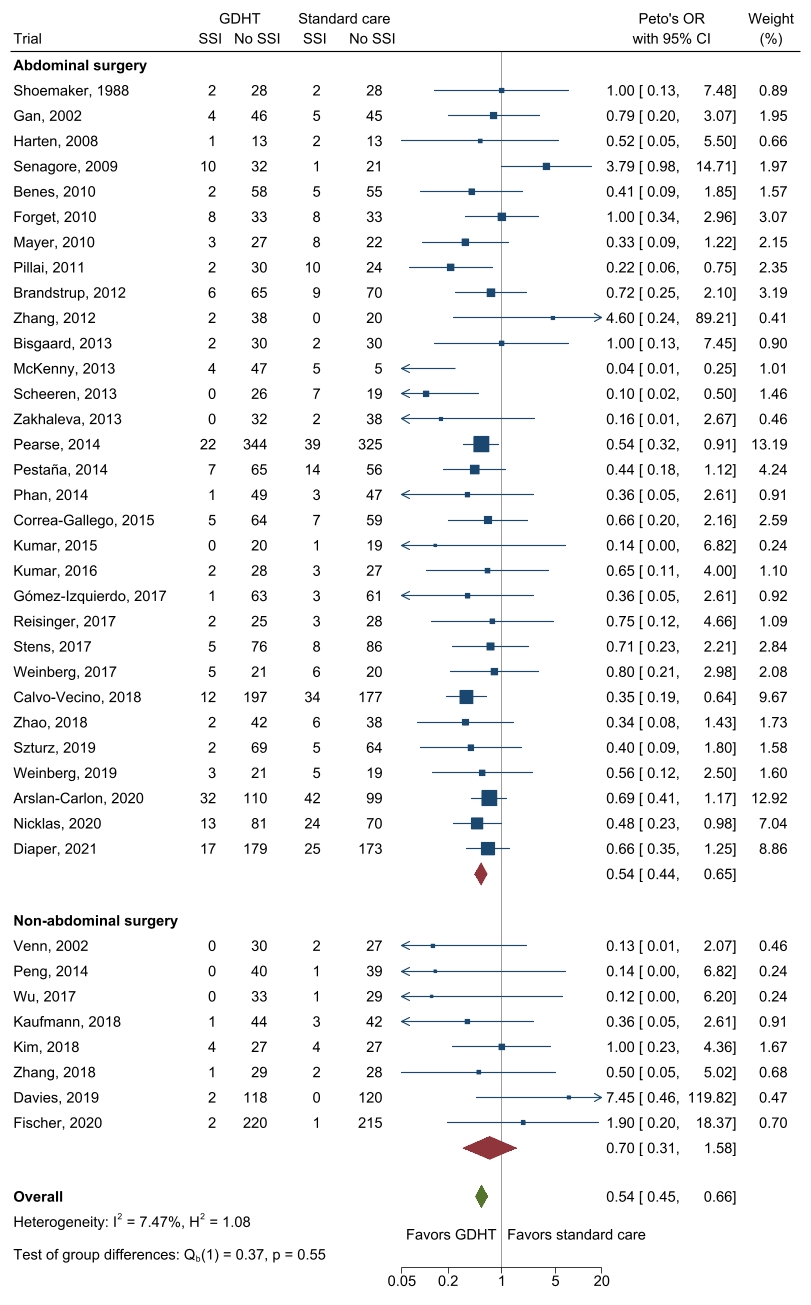


Fixed inverse-variance (Peto’s method for odds ratio) meta-analysis of GDHT compared to standard care for surgical site infections. Trials are subgrouped according to surgery type. For definitions see section ”Outcomes: Definitions, data synthesis, and sensitivity analyses”.

Abbreviations: **SSI:** Surgical site infection

### eFigure 56: Surgical site infection - Sensitivity analysis 1: Excluding trials with high risk of bias

**
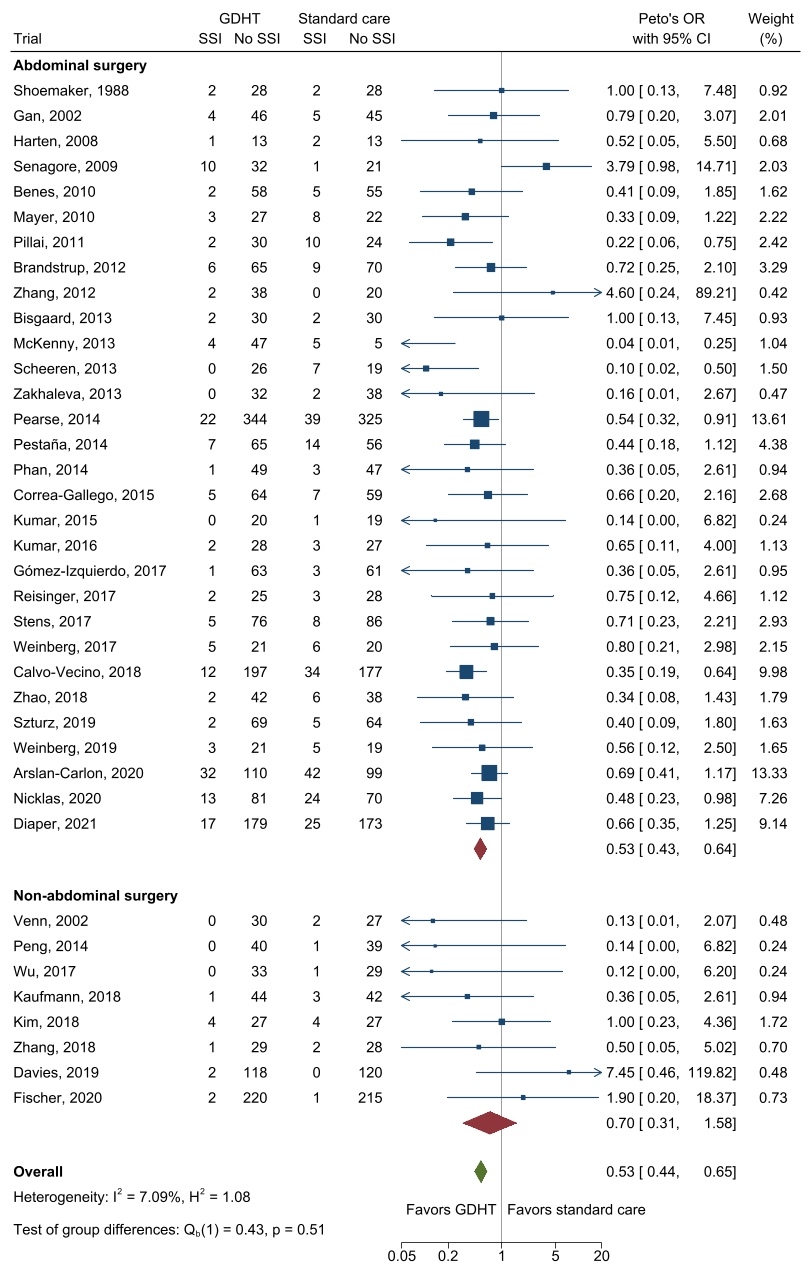
**

Fixed inverse-variance (Peto’s method for odds ratio) meta-analysis of GDHT compared to standard care for surgical site infections without high risk of bias-trials. Trials are subgrouped according to surgery type. For definitions see section ”Outcomes: Definitions, data synthesis, and sensitivity analyses”.

Abbreviations: **SSI:** Surgical site infection

### eFigure 57: Surgical site infection - Sensitivity analysis 2: Excluding trials without an outcome definition


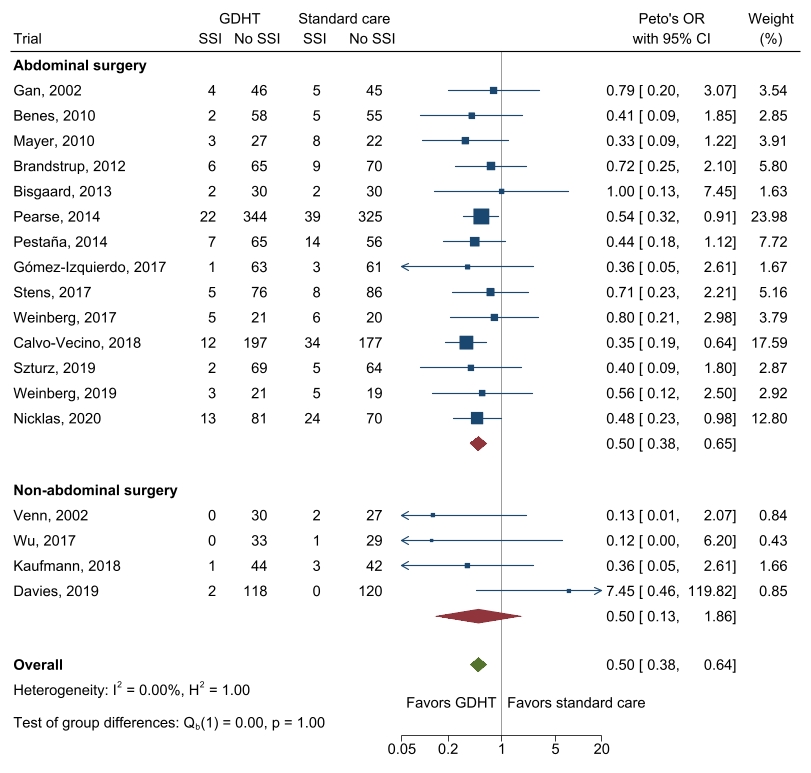


Fixed inverse-variance (Peto’s method for odds ratio) meta-analysis of GDHT compared to standard care for surgical site infections. Included trials reported a clear outcome definition. Trials are subgrouped according to surgery type. For definitions see section ”Outcomes: Definitions, data synthesis, and sensitivity analyses”.

One trial that reported an outcome definition had no events in either group and were therefore excluded from the analysis.

Abbreviations: **SSI:** Surgical site infection

### eFigure 58: Surgical site infection - Sensitivity analysis 3: Only including trials with established definitions


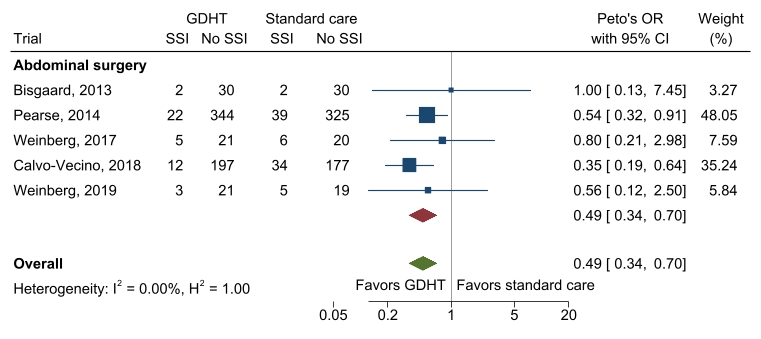


Fixed inverse-variance (Peto’s method for odds ratio) meta-analysis of GDHT compared to standard care for surgical site infections. Included trials used very similar guideline definitions. Trials are subgrouped according to surgery type. For definitions see section ”Outcomes: Definitions, data synthesis, and sensitivity analyses”.

Abbreviations: **SSI:** Surgical site infection

## Paralytic ileus

### eFigure 59: Paralytic ileus - Primary analysis


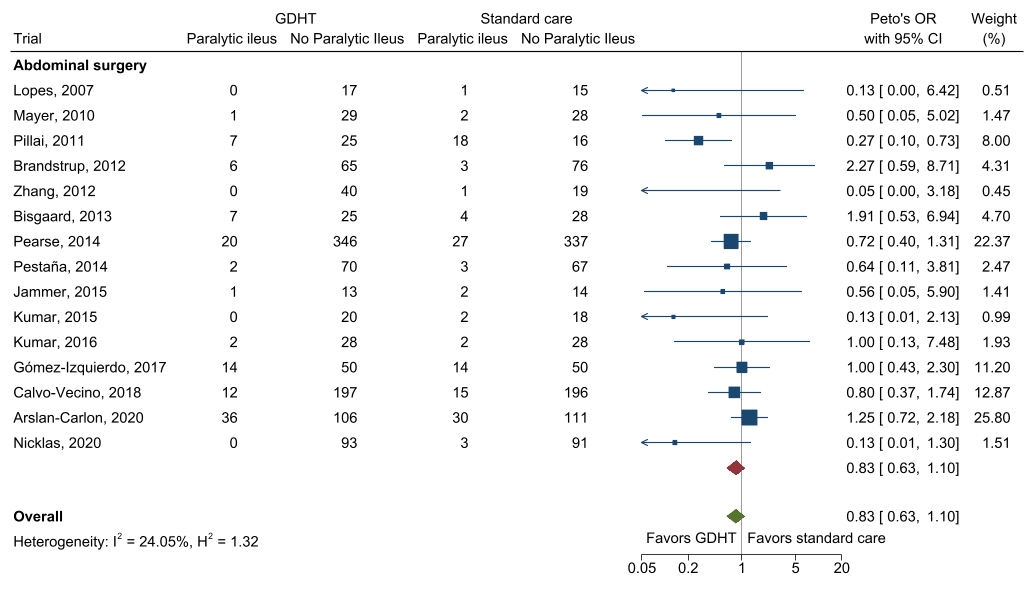


Fixed inverse-variance (Peto’s method for odds ratio) meta-analysis of GDHT compared to standard care for paralytic ileus. Only trials that specifically reported paralytic ileus are included. For definitions see section ”Outcomes: Definitions, data synthesis, and sensitivity analyses”.

### eFigure 60: Paralytic ileus - Sensitivity analysis 1: Excluding trials with high risk of bias


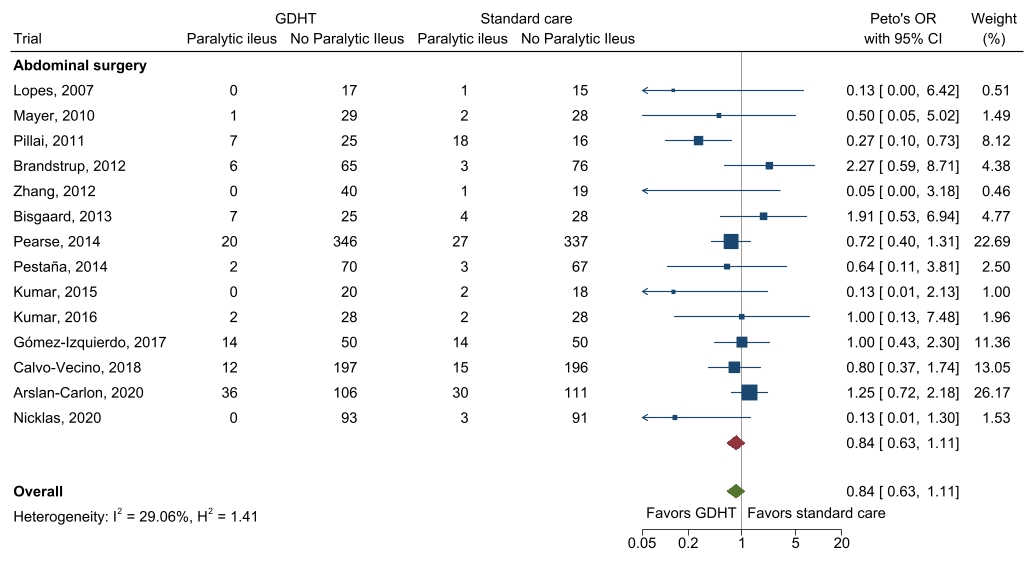


Fixed inverse-variance (Peto’s method for odds ratio) meta-analysis of GDHT compared to standard care for paralytic ileus without high risk of bias-trials. Only trials that specifically reported paralytic ileus are included. For definitions see section ”Outcomes: Definitions, data synthesis, and sensitivity analyses”.

### eFigure 61: Paralytic Ileus - Sensitivity analysis 2: All trials reporting the outcome


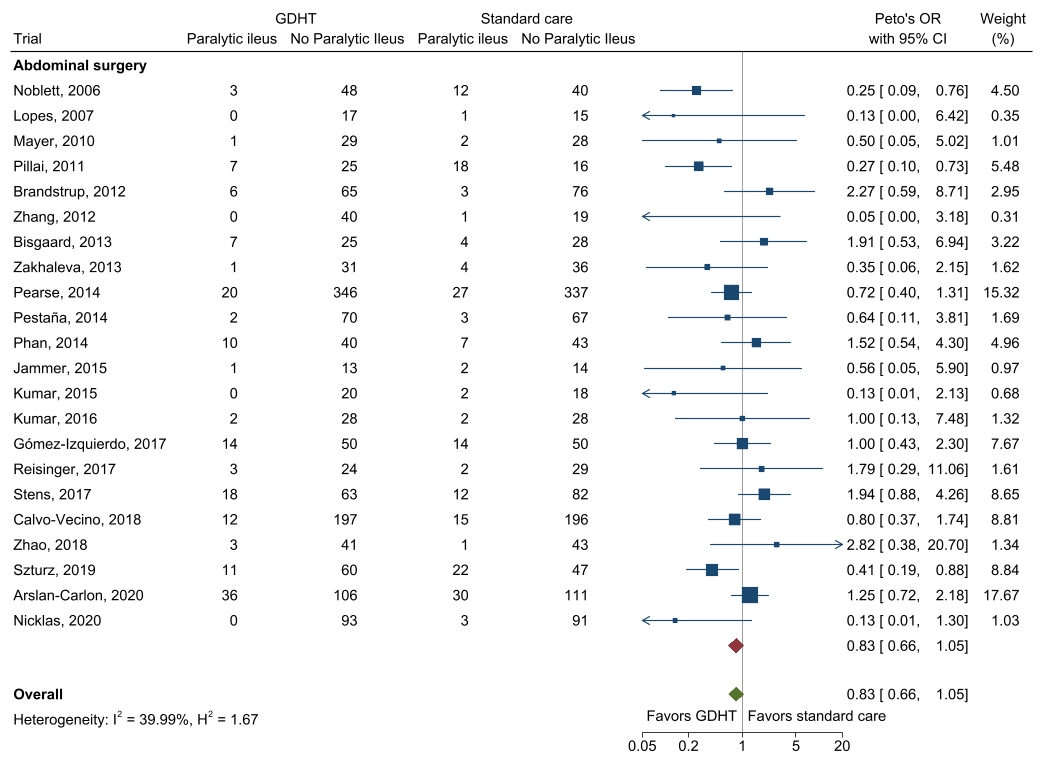


Fixed inverse-variance (Peto’s method for odds ratio) meta-analysis of GDHT compared to standard care for ileus. All trials that reported paralytic ileus or unspecified ileus are included. For definitions see section ”Outcomes: Definitions, data synthesis, and sensitivity analyses”.

## Anastomotic leakage

### eFigure 62: Anastomotic leakage - Primary analysis


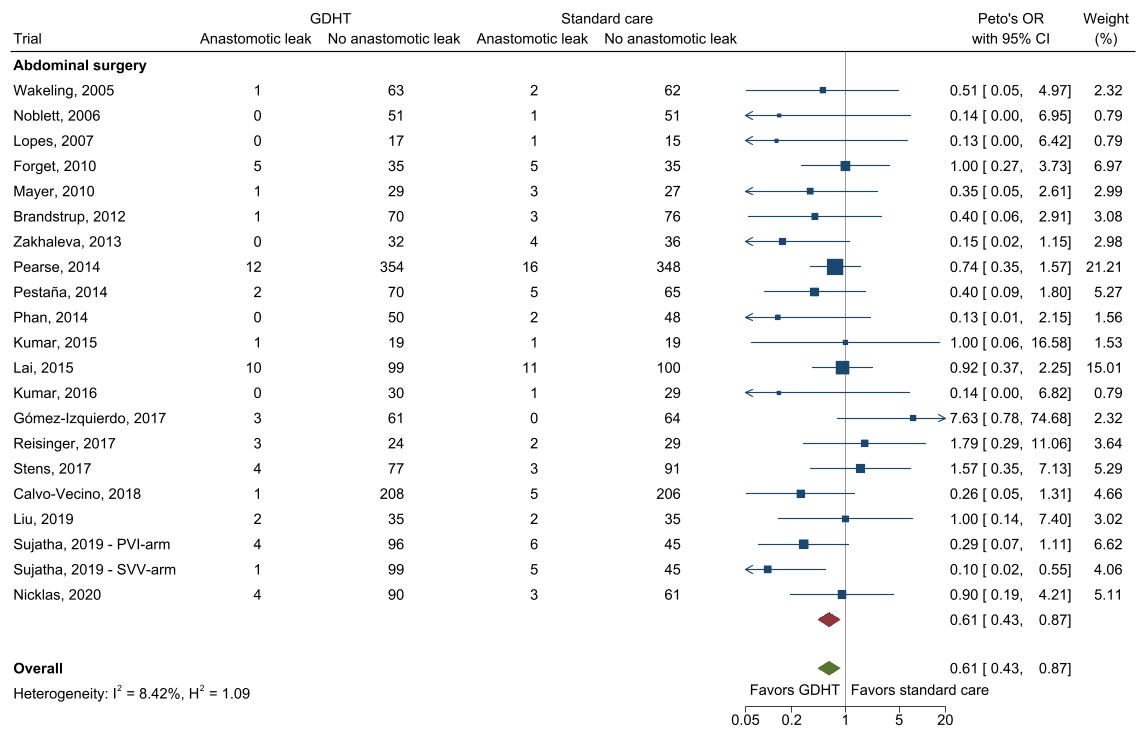


Fixed inverse-variance (Peto’s method for odds ratio) meta-analysis of GDHT compared to standard care for anastomotic leakage. All trials that reported the outcome are included. For definitions see section ”Outcomes: Definitions, data synthesis, and sensitivity analyses”.

### eFigure 63: Anastomotic leakage - Sensitivity analysis 1: Excluding trials with high risk of bias


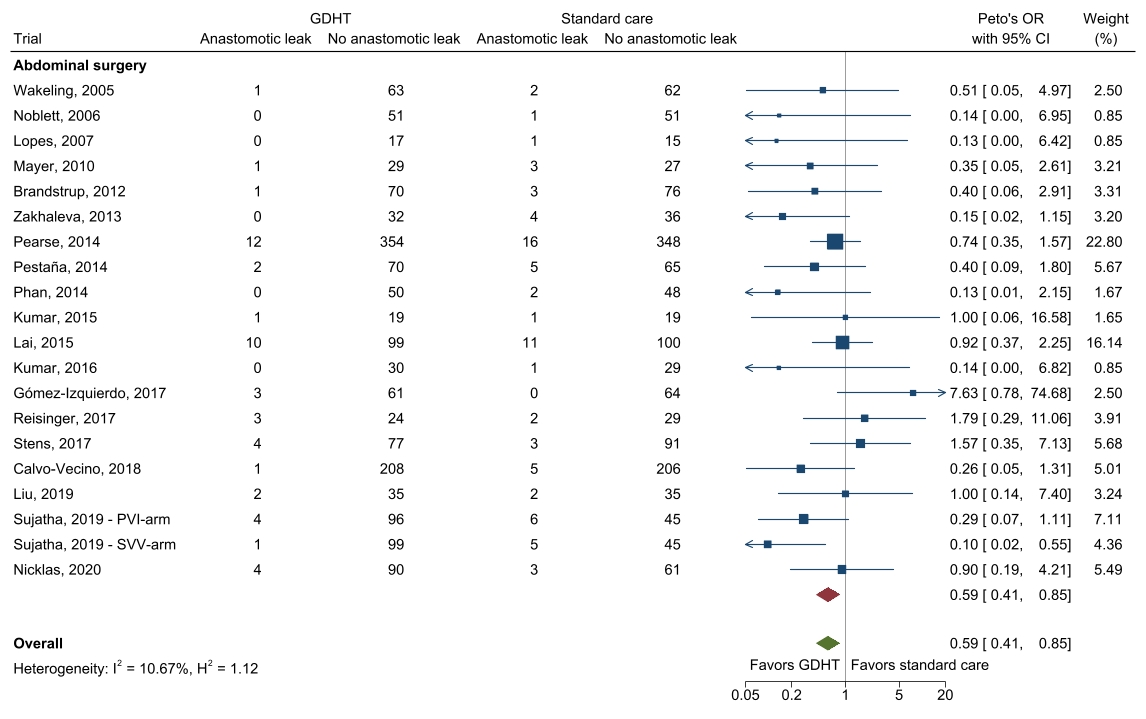


Fixed inverse-variance (Peto’s method for odds ratio) meta-analysis of GDHT compared to standard care for anastomotic leakage. Trials with a high risk bias are excluded. For definitions see section ”Outcomes: Definitions, data synthesis, and sensitivity analyses”.

### eFigure 64: Anastomotic leakage - Sensitivity analysis 2: Only including trials with a definition based on radiology


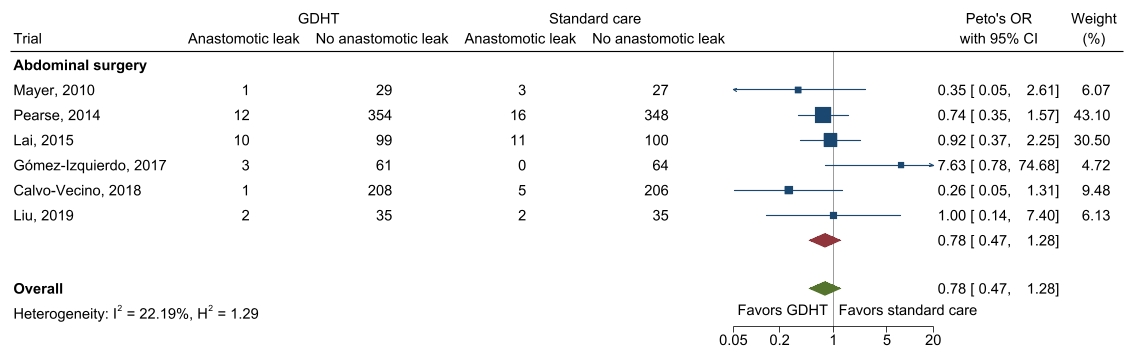


Fixed inverse-variance (Peto’s method for odds ratio) meta-analysis of GDHT compared to standard care for anastomotic leakage. Only trials that accepted radiologic evidence without requiring reoperation are included. For definitions see section ”Outcomes: Definitions, data synthesis, and sensitivity analyses”

## Delirium

### eFigure 65: Delirium - Primary analysis with abdominal surgery vs. non-abdominal surgery


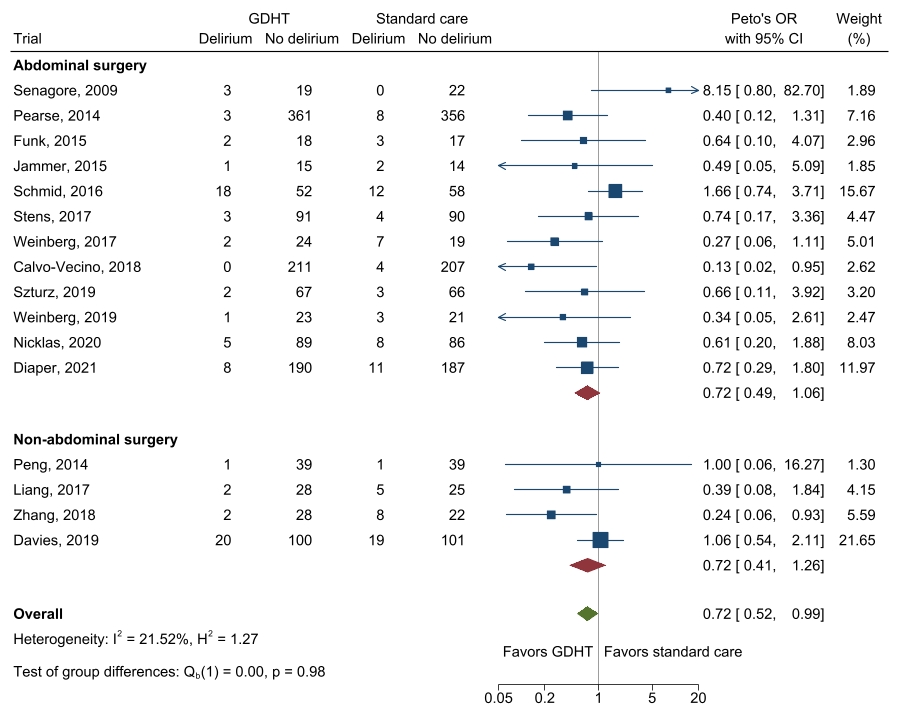


Fixed inverse-variance (Peto’s method for odds ratio) meta-analysis of GDHT compared to standard care for delirium. All trials that reported the outcome are included. Trials are subgrouped according to surgery type. For definitions see section ”Outcomes: Definitions, data synthesis, and sensitivity analyses”.

### eFigure 66: Delirium - Sensitivity analysis 1: Excluding trials with high risk of bias


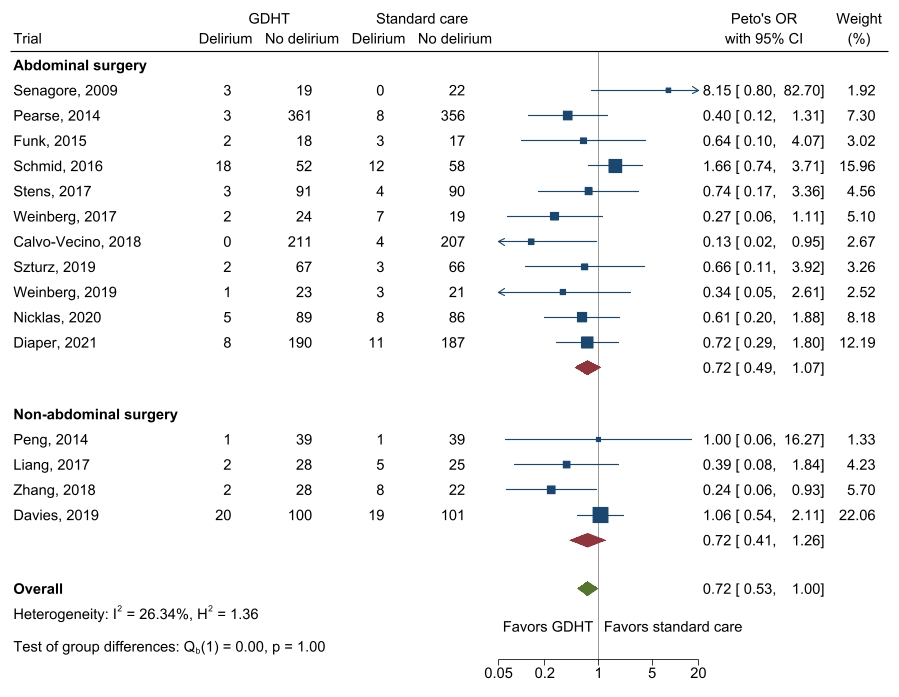


Fixed inverse-variance (Peto’s method for odds ratio) meta-analysis of GDHT compared to standard care for delirium without high risk of bias-trials. Trials are subgrouped according to surgery type. For definitions see section ”Outcomes: Definitions, data synthesis, and sensitivity analyses”.

### eFigure 67: Delirium - Sensitivity analysis 2: Only including trials with EPCO 2015-definition


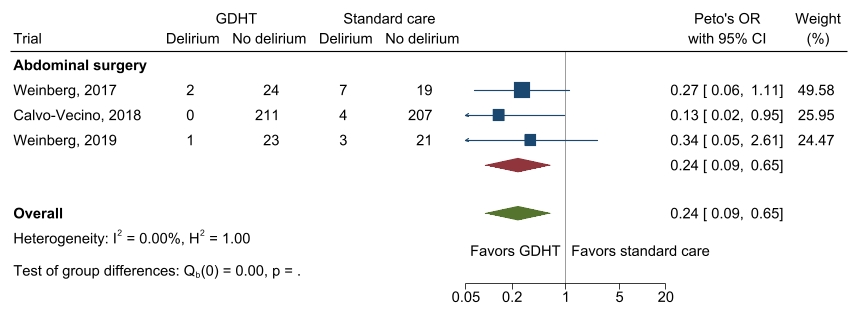


Fixed inverse-variance (Peto’s method for odds ratio) meta-analysis of GDHT compared to standard care for delirium in trials using EPCO-2015 definitions^2^. For definitions see section ”Outcomes: Definitions, data synthesis, and sensitivity analyses”.

# PRISMA-checklist

| **Section and Topic** | **Item #** | **Checklist item** | **Location where item is reported** |
| --- | --- | --- | --- |
| **TITLE** | | |  |
| Title | 1 | Identify the report as a systematic review. | Title, Page 1 |
| **ABSTRACT** | | |  |
| Abstract | 2 | See the PRISMA 2020 for Abstracts checklist. | Page 3 |
| **INTRODUCTION** | | |  |
| Rationale | 3 | Describe the rationale for the review in the context of existing knowledge. | Page 4 |
| Objectives | 4 | Provide an explicit statement of the objective(s) or question(s) the review addresses. | Page 4 |
| **METHODS** | | |  |
| Eligibility criteria | 5 | Specify the inclusion and exclusion criteria for the review and how studies were grouped for the syntheses. | Page 5 |
| Information sources | 6 | Specify all databases, registers, websites, organizations, reference lists and other sources searched or consulted to identify studies. Specify the date when each source was last searched or consulted. | Page 6 |
| Search strategy | 7 | Present the full search strategies for all databases, registers and websites, including any filters and limits used. | Supplement 1 |
| Selection process | 8 | Specify the methods used to decide whether a study met the inclusion criteria of the review, including how many reviewers screened each record and each report retrieved, whether they worked independently, and if applicable, details of automation tools used in the process. | Page 6 |
| Data collection process | 9 | Specify the methods used to collect data from reports, including how many reviewers collected data from each report, whether they worked independently, any processes for obtaining or confirming data from study investigators, and if applicable, details of automation tools used in the process. | Page 6 |
| Data items | 10a | List and define all outcomes for which data were sought. Specify whether all results that were compatible with each outcome domain in each study were sought (e.g. for all measures, time points, analyses), and if not, the methods used to decide which results to collect. | Page 6+7, Supplemental 2 |
|  | 10b | List and define all other variables for which data were sought (e.g. participant and intervention characteristics, funding sources). Describe any assumptions made about any missing or unclear information. | Page 6+7, Supplemental 2 |
| Study risk of bias assessment | 11 | Specify the methods used to assess risk of bias in the included studies, including details of the tool(s) used, how many reviewers assessed each study and whether they worked independently, and if applicable, details of automation tools used in the process. | Page 6, Supplemental 2 |
| Effect measures | 12 | Specify for each outcome the effect measure(s) (e.g. risk ratio, mean difference) used in the synthesis or presentation of results. | Page 7-8 |
| Synthesis methods | 13a | Describe the processes used to decide which studies were eligible for each synthesis (e.g. tabulating the study intervention characteristics and comparing against the planned groups for each synthesis (item #5)). | Page 6-7, Supplemental 2 |
|  | 13b | Describe any methods required to prepare the data for presentation or synthesis, such as handling of missing summary statistics, or data conversions. | Page 7-8 |
|  | 13c | Describe any methods used to tabulate or visually display results of individual studies and syntheses. | Page 7-8 |
|  | 13d | Describe any methods used to synthesize results and provide a rationale for the choice(s). If meta-analysis was performed, describe the model(s), method(s) to identify the presence and extent of statistical heterogeneity, and software package(s) used. | Page 7-8, Supplemental 2 |
|  | 13e | Describe any methods used to explore possible causes of heterogeneity among study results (e.g. subgroup analysis, meta-regression). | Page 7-8, Supplemental 2 |
|  | 13f | Describe any sensitivity analyses conducted to assess robustness of the synthesized results. | Page 7-8, Supplemental 2 |
| Reporting bias assessment | 14 | Describe any methods used to assess risk of bias due to missing results in a synthesis (arising from reporting biases). | Page 6, Supplemental 2 |
| Certainty assessment | 15 | Describe any methods used to assess certainty (or confidence) in the body of evidence for an outcome. | Page 8, Supplemental 2 |
| **RESULTS** | | |  |
| Study selection | 16a | Describe the results of the search and selection process, from the number of records identified in the search to the number of studies included in the review, ideally using a flow diagram. | Page 9, Figure in Supplemental 2 |
|  | 16b | Cite studies that might appear to meet the inclusion criteria, but which were excluded, and explain why they were excluded. | Page 9, Supplemental 2 |
| Study characteristics | 17 | Cite each included study and present its characteristics. | Tabel 1, Supplemental 2 |
| Risk of bias in studies | 18 | Present assessments of risk of bias for each included study. | Supplemental 2 |
| Results of individual studies | 19 | For all outcomes, present, for each study: (a) summary statistics for each group (where appropriate) and (b) an effect estimate and its precision (e.g. confidence/credible interval), ideally using structured tables or plots. | Page 9-11, Supplemental 2 |
| Results of syntheses | 20a | For each synthesis, briefly summarize the characteristics and risk of bias among contributing studies. | Page 9-11, Supplemental 2 |
|  | 20b | Present results of all statistical syntheses conducted. If meta-analysis was done, present for each the summary estimate and its precision (e.g. confidence/credible interval) and measures of statistical heterogeneity. If comparing groups, describe the direction of the effect. | Page 9-11, Supplemental 2 |
|  | 20c | Present results of all investigations of possible causes of heterogeneity among study results. | Page 9-11, Supplemental 2 |
|  | 20d | Present results of all sensitivity analyses conducted to assess the robustness of the synthesized results. | Page 9-11, Supplemental 2 |
| Reporting biases | 21 | Present assessments of risk of bias due to missing results (arising from reporting biases) for each synthesis assessed. | Page 9, Supplemental 2 |
| Certainty of evidence | 22 | Present assessments of certainty (or confidence) in the body of evidence for each outcome assessed. | Page 11, Supplemental 2 |
| **DISCUSSION** | | |  |
| Discussion | 23a | Provide a general interpretation of the results in the context of other evidence. | Page 12 |
|  | 23b | Discuss any limitations of the evidence included in the review. | Page 12-13 |
|  | 23c | Discuss any limitations of the review processes used. | Page 13-14 |
|  | 23d | Discuss implications of the results for practice, policy, and future research. | Page 12-14 |
| **OTHER INFORMATION** | | |  |
| Registration and protocol | 24a | Provide registration information for the review, including register name and registration number, or state that the review was not registered. | Page 2+5 |
|  | 24b | Indicate where the review protocol can be accessed, or state that a protocol was not prepared. | Page 2+5 |
|  | 24c | Describe and explain any amendments to information provided at registration or in the protocol. | Page 2+5 |
| Support | 25 | Describe sources of financial or non-financial support for the review, and the role of the funders or sponsors in the review. | Page 15 |
| Competing interests | 26 | Declare any competing interests of review authors. | Page 15 |
| Availability of data, code and other materials | 27 | Report which of the following are publicly available and where they can be found: template data collection forms; data extracted from included studies; data used for all analyses; analytic code; any other materials used in the review. | Supplemental 2 |

# REFERENCES

1. Higgins J, Sterne J, Savović J, et al. *A revised tool for assessing risk of bias in randomized trials. Cochrane Database of Systematic Reviews* 2016, Issue 10 (Suppl 1).

2. Jammer I, Wickboldt N, Sander M, et al. Standards for definitions and use of outcome measures for clinical effectiveness research in perioperative medicine: European Perioperative Clinical Outcome (EPCO) definitions: a statement from the ESA-ESICM joint taskforce on perioperative outcome measures. *Eur J Anaesthesiol.* 2015;32(2):88-105.

3. Horan TC, Andrus M, Dudeck MA. CDC/NHSN surveillance definition of health care-associated infection and criteria for specific types of infections in the acute care setting. *Am J Infect Control.* 2008;36(5):309-332.

4. Copeland GP, Jones D, Walters M. POSSUM: a scoring system for surgical audit. *Br J Surg.* 1991;78(3):355-360.

5. User Guide for the 2014 ACS NSQIP Participant Use Data File (PUF). *American College of Surgeons National Surgical Quality Improvement Program (ACS NSQIP: User Guide for the 2014 ACS NSQIP Participant Use Data File (PUF)* 2015. Accessed June 22nd 2021, 2015.

6. Shi J, Luo D, Weng H, et al. Optimally estimating the sample standard deviation from the five-number summary. *Res Synth Methods.* 2020;11(5):641-654.

7. Calvo-Vecino JM, Ripollés-Melchor J, Mythen MG, et al. Effect of goal-directed haemodynamic therapy on postoperative complications in low-moderate risk surgical patients: a multicentre randomised controlled trial (FEDORA trial). *Br J Anaesth.* 2018;120(4):734-744.

8. Buettner M, Schummer W, Huettemann E, Schenke S, van Hout N, Sakka SG. Influence of systolic-pressure-variation-guided intraoperative fluid management on organ function and oxygen transport. *Br J Anaesth.* 2008;101(2):194-199.

9. Guidelines for the management of adults with hospital-acquired, ventilator-associated, and healthcare-associated pneumonia. *Am J Respir Crit Care Med.* 2005;171(4):388-416.

10. Ranieri VM, Rubenfeld GD, Thompson BT, et al. Acute respiratory distress syndrome: the Berlin Definition. *JAMA.* 2012;307(23):2526-2533.

11. Thygesen K, Alpert JS, Jaffe AS, et al. Third universal definition of myocardial infarction. *Circulation.* 2012;126(16):2020-2035.

12. Bellomo R, Kellum JA, Ronco C. Defining and classifying acute renal failure: from advocacy to consensus and validation of the RIFLE criteria. *Intensive Care Med.* 2007;33(3):409-413.

13. Stevens PE, Levin A. Evaluation and management of chronic kidney disease: synopsis of the kidney disease: improving global outcomes 2012 clinical practice guideline. *Ann Intern Med.* 2013;158(11):825-830.

14. Guyatt GH, Oxman AD, Kunz R, et al. GRADE guidelines 6. Rating the quality of evidence--imprecision. *J Clin Epidemiol.* 2011;64(12):1283-1293.

15. Shoemaker WC, Appel PL, Kram HB, Waxman K, Lee TS. Prospective trial of supranormal values of survivors as therapeutic goals in high-risk surgical patients. *Chest.* 1988;94(6):1176-1186.

16. Sandham JD, Hull RD, Brant RF, et al. A randomized, controlled trial of the use of pulmonary-artery catheters in high-risk surgical patients. *N Engl J Med.* 2003;348(1):5-14.

17. Corbella D, Toppin PJ, Ghanekar A, et al. Cardiac output-based fluid optimization for kidney transplant recipients: a proof-of-concept trial. *Can J Anaesth.* 2018;65(8):873-883.

18. Nethan CL, S.; Cheepcharoenrat, N.; Lorsomradee S. Effects of the perioperative prevention of compensated shock during major abdominal surgery on the enhanced recovery after surgery. *Journal of Practical Shock.* 2018;2(1):61-64.

19. Wu QF, Kong H, Xu ZZ, Li HJ, Mu DL, Wang DX. Impact of goal-directed hemodynamic management on the incidence of acute kidney injury in patients undergoing partial nephrectomy: a pilot randomized controlled trial. *BMC Anesthesiol.* 2021;21(1):67.

20. Lobo SM, Salgado PF, Castillo VG, et al. Effects of maximizing oxygen delivery on morbidity and mortality in high-risk surgical patients. *Crit Care Med.* 2000;28(10):3396-3404.

21. Abdullah MH, Hasanin AS, Mahmoud FM. Goal directed fluid optimization using Pleth variability index versus corrected flow time in cirrhotic patients undergoing major abdominal surgeries. *Egyptian Journal of Anaesthesia.* 2012;28(1):23-28.

22. Wang P, Wang HW, Zhong TD. Effect of stroke volume variability- guided intraoperative fluid restriction on gastrointestinal functional recovery. *Hepatogastroenterology.* 2012;59(120):2457-2460.

23. Choi SS, Jun IG, Cho SS, Kim SK, Hwang GS, Kim YK. Effect of stroke volume variation-directed fluid management on blood loss during living-donor right hepatectomy: a randomised controlled study. *Anaesthesia.* 2015;70(11):1250-1258.

24. Feldheiser A, Pavlova V, Weimann K, et al. Haemodynamic Optimization by Oesophageal Doppler and Pulse Power Wave Analysis in Liver Surgery: A Randomised Controlled Trial. *PLoS One.* 2015;10(7):e0132715.

25. Pavlovic G, Diaper J, Ellenberger C, et al. Impact of early haemodynamic goal-directed therapy in patients undergoing emergency surgery: an open prospective, randomised trial. *J Clin Monit Comput.* 2016;30(1):87-99.

26. Hassan HF, Ali MZ, Refaat AI, et al. Pulse-induced Continuous Cardiac Output (PICCO) versus Trans-Esophageal Doppler Monitor (TED ) for optimization of fluid management in patients undergoing major abdominal surgery. a comparative study. *Anesthesia and Analgesia.* 2016;122(5):S344.

27. Kong YG, Kim JY, Yu J, Lim J, Hwang JH, Kim YK. Efficacy and Safety of Stroke Volume Variation-Guided Fluid Therapy for Reducing Blood Loss and Transfusion Requirements During Radical Cystectomy: A Randomized Clinical Trial. *Medicine (Baltimore).* 2016;95(19):e3685.

28. Seo H, Jun IG, Ha TY, Hwang S, Lee SG, Kim YK. High Stroke Volume Variation Method by Mannitol Administration Can Decrease Blood Loss During Donor Hepatectomy. *Medicine (Baltimore).* 2016;95(2):e2328.

29. Warnakulasuriya SR, Davies SJ, Wilson RJ, Yates DR. Comparison of esophageal Doppler and plethysmographic variability index to guide intraoperative fluid therapy for low-risk patients undergoing colorectal surgery. *J Clin Anesth.* 2016;34:600-608.

30. Wu CY, Lin YS, Tseng HM, et al. Comparison of two stroke volume variation-based goal-directed fluid therapies for supratentorial brain tumour resection: a randomized controlled trial. *Br J Anaesth.* 2017;119(5):934-942.

31. Bahlmann H, Hahn RG, Nilsson L. Pleth variability index or stroke volume optimization during open abdominal surgery: a randomized controlled trial. *BMC Anesthesiol.* 2018;18(1):115.

32. Coeckelenbergh S, Delaporte A, Ghoundiwal D, et al. Pleth variability index versus pulse pressure variation for intraoperative goal-directed fluid therapy in patients undergoing low-to-moderate risk abdominal surgery: a randomized controlled trial. *BMC Anesthesiol.* 2019;19(1):34.

33. Lee YH, Jang HW, Park CH, et al. Changes in plasma volume before and after major abdominal surgery following stroke volume variation-guided fluid therapy: a randomized controlled trial. *Minerva Anestesiol.* 2020;86(5):507-517.

34. Wang K, Du BX, Zhang Y, et al. The benefits of a variable svv threshold using cardiopulmonary ultrasound to guide perioperative fluid therapy in patients with ards. *International Journal of Clinical and Experimental Medicine.* 2020;13(4):2421-2430.

35. Venn R, Steele A, Richardson P, Poloniecki J, Grounds M, Newman P. Randomized controlled trial to investigate influence of the fluid challenge on duration of hospital stay and perioperative morbidity in patients with hip fractures. *Br J Anaesth.* 2002;88(1):65-71.

36. Donati A, Loggi S, Preiser JC, et al. Goal-directed intraoperative therapy reduces morbidity and length of hospital stay in high-risk surgical patients. *Chest.* 2007;132(6):1817-1824.

37. Szakmany T, Toth I, Kovacs Z, et al. Effects of volumetric vs. pressure-guided fluid therapy on postoperative inflammatory response: a prospective, randomized clinical trial. *Intensive Care Med.* 2005;31(5):656-663.

38. Cohn SM, Pearl RG, Acosta SM, et al. A prospective randomized pilot study of near-infrared spectroscopy-directed restricted fluid therapy versus standard fluid therapy in patients undergoing elective colorectal surgery. *Am Surg.* 2010;76(12):1384-1392.

39. Jammer I, Ulvik A, Erichsen C, Lødemel O, Ostgaard G. Does central venous oxygen saturation-directed fluid therapy affect postoperative morbidity after colorectal surgery? A randomized assessor-blinded controlled trial. *Anesthesiology.* 2010;113(5):1072-1080.

40. Mikor A, Trásy D, Németh MF, et al. Continuous central venous oxygen saturation assisted intraoperative hemodynamic management during major abdominal surgery: a randomized, controlled trial. *BMC Anesthesiol.* 2015;15:82.

41. Li B, Zhang L, Zhang S, Wang B. Application of ultrasound in goal-directed fluid management of anesthesia in elderly patients. *Biomedical Research (India).* 2017;2017(Special Issue HealthScienceandBioConvergenceTechnologyEdition-II):S482-S486.

42. Feng ZY, Xu X, Zhu SM, Bein B, Zheng SS. Effects of low central venous pressure during preanhepatic phase on blood loss and liver and renal function in liver transplantation. *World J Surg.* 2010;34(8):1864-1873.

43. Bender JS, Smith-Meek MA, Jones CE. Routine pulmonary artery catheterization does not reduce morbidity and mortality of elective vascular surgery: results of a prospective, randomized trial. *Ann Surg.* 1997;226(3):229-236; discussion 236-227.

44. Sinclair S, James S, Singer M. Intraoperative intravascular volume optimisation and length of hospital stay after repair of proximal femoral fracture: randomised controlled trial. *BMJ.* 1997;315(7113):909-912.

45. Conway DH, Mayall R, Abdul-Latif MS, Gilligan S, Tackaberry C. Randomised controlled trial investigating the influence of intravenous fluid titration using oesophageal Doppler monitoring during bowel surgery. *Anaesthesia.* 2002;57(9):845-849.

46. Gan TJ, Soppitt A, Maroof M, et al. Goal-directed intraoperative fluid administration reduces length of hospital stay after major surgery. *Anesthesiology.* 2002;97(4):820-826.

47. Wakeling HG, McFall MR, Jenkins CS, et al. Intraoperative oesophageal Doppler guided fluid management shortens postoperative hospital stay after major bowel surgery. *Br J Anaesth.* 2005;95(5):634-642.

48. Noblett SE, Snowden CP, Shenton BK, Horgan AF. Randomized clinical trial assessing the effect of Doppler-optimized fluid management on outcome after elective colorectal resection. *Br J Surg.* 2006;93(9):1069-1076.

49. Lopes MR, Oliveira MA, Pereira VO, Lemos IP, Auler JO, Jr., Michard F. Goal-directed fluid management based on pulse pressure variation monitoring during high-risk surgery: a pilot randomized controlled trial. *Crit Care.* 2007;11(5):R100.

50. Harten J, Crozier JE, McCreath B, et al. Effect of intraoperative fluid optimisation on renal function in patients undergoing emergency abdominal surgery: a randomised controlled pilot study (ISRCTN 11799696). *Int J Surg.* 2008;6(3):197-204.

51. Senagore AJ, Emery T, Luchtefeld M, Kim D, Dujovny N, Hoedema R. Fluid management for laparoscopic colectomy: a prospective, randomized assessment of goal-directed administration of balanced salt solution or hetastarch coupled with an enhanced recovery program. *Dis Colon Rectum.* 2009;52(12):1935-1940.

52. Benes J, Chytra I, Altmann P, et al. Intraoperative fluid optimization using stroke volume variation in high risk surgical patients: results of prospective randomized study. *Crit Care.* 2010;14(3):R118.

53. Forget P, Lois F, de Kock M. Goal-directed fluid management based on the pulse oximeter-derived pleth variability index reduces lactate levels and improves fluid management. *Anesth Analg.* 2010;111(4):910-914.

54. Mayer J, Boldt J, Mengistu AM, Röhm KD, Suttner S. Goal-directed intraoperative therapy based on autocalibrated arterial pressure waveform analysis reduces hospital stay in high-risk surgical patients: a randomized, controlled trial. *Crit Care.* 2010;14(1):R18.

55. Van der Linden PJ, Dierick A, Wilmin S, Bellens B, De Hert SG. A randomized controlled trial comparing an intraoperative goal-directed strategy with routine clinical practice in patients undergoing peripheral arterial surgery. *Eur J Anaesthesiol.* 2010;27(9):788-793.

56. Pillai P, McEleavy I, Gaughan M, et al. A double-blind randomized controlled clinical trial to assess the effect of Doppler optimized intraoperative fluid management on outcome following radical cystectomy. *J Urol.* 2011;186(6):2201-2206.

57. Brandstrup B, Svendsen PE, Rasmussen M, et al. Which goal for fluid therapy during colorectal surgery is followed by the best outcome: near-maximal stroke volume or zero fluid balance? *Br J Anaesth.* 2012;109(2):191-199.

58. Challand C, Struthers R, Sneyd JR, et al. Randomized controlled trial of intraoperative goal-directed fluid therapy in aerobically fit and unfit patients having major colorectal surgery. *Br J Anaesth.* 2012;108(1):53-62.

59. Zhang J, Qiao H, He Z, Wang Y, Che X, Liang W. Intraoperative fluid management in open gastrointestinal surgery: goal-directed versus restrictive. *Clinics (Sao Paulo).* 2012;67(10):1149-1155.

60. Bisgaard J, Gilsaa T, Rønholm E, Toft P. Optimising stroke volume and oxygen delivery in abdominal aortic surgery: a randomised controlled trial. *Acta Anaesthesiol Scand.* 2013;57(2):178-188.

61. Bundgaard-Nielsen M, Jans Ø, Müller RG, et al. Does goal-directed fluid therapy affect postoperative orthostatic intolerance?: A randomized trial. *Anesthesiology.* 2013;119(4):813-823.

62. El Sharkawy OA, Refaat EK, Ibraheem AE, et al. Transoesophageal Doppler compared to central venous pressure for perioperative hemodynamic monitoring and fluid guidance in liver resection. *Saudi J Anaesth.* 2013;7(4):378-386.

63. McKenny M, Conroy P, Wong A, et al. A randomised prospective trial of intra-operative oesophageal Doppler-guided fluid administration in major gynaecological surgery. *Anaesthesia.* 2013;68(12):1224-1231.

64. Ramsingh DS, Sanghvi C, Gamboa J, Cannesson M, Applegate RL, 2nd. Outcome impact of goal directed fluid therapy during high risk abdominal surgery in low to moderate risk patients: a randomized controlled trial. *J Clin Monit Comput.* 2013;27(3):249-257.

65. Salzwedel C, Puig J, Carstens A, et al. Perioperative goal-directed hemodynamic therapy based on radial arterial pulse pressure variation and continuous cardiac index trending reduces postoperative complications after major abdominal surgery: a multi-center, prospective, randomized study. *Crit Care.* 2013;17(5):R191.

66. Scheeren TW, Wiesenack C, Gerlach H, Marx G. Goal-directed intraoperative fluid therapy guided by stroke volume and its variation in high-risk surgical patients: a prospective randomized multicentre study. *J Clin Monit Comput.* 2013;27(3):225-233.

67. Srinivasa S, Taylor MH, Singh PP, Yu TC, Soop M, Hill AG. Randomized clinical trial of goal-directed fluid therapy within an enhanced recovery protocol for elective colectomy. *Br J Surg.* 2013;100(1):66-74.

68. Zakhaleva J, Tam J, Denoya PI, Bishawi M, Bergamaschi R. The impact of intravenous fluid administration on complication rates in bowel surgery within an enhanced recovery protocol: a randomized controlled trial. *Colorectal Dis.* 2013;15(7):892-899.

69. Zheng H, Guo H, Ye JR, Chen L, Ma HP. Goal-directed fluid therapy in gastrointestinal surgery in older coronary heart disease patients: randomized trial. *World J Surg.* 2013;37(12):2820-2829.

70. Pearse RM, Harrison DA, MacDonald N, et al. Effect of a perioperative, cardiac output-guided hemodynamic therapy algorithm on outcomes following major gastrointestinal surgery: a randomized clinical trial and systematic review. *JAMA.* 2014;311(21):2181-2190.

71. Peng K, Li J, Cheng H, Ji FH. Goal-directed fluid therapy based on stroke volume variations improves fluid management and gastrointestinal perfusion in patients undergoing major orthopedic surgery. *Med Princ Pract.* 2014;23(5):413-420.

72. Pestaña D, Espinosa E, Eden A, et al. Perioperative goal-directed hemodynamic optimization using noninvasive cardiac output monitoring in major abdominal surgery: a prospective, randomized, multicenter, pragmatic trial: POEMAS Study (PeriOperative goal-directed thErapy in Major Abdominal Surgery). *Anesth Analg.* 2014;119(3):579-587.

73. Phan TD, D'Souza B, Rattray MJ, Johnston MJ, Cowie BS. A randomised controlled trial of fluid restriction compared to oesophageal Doppler-guided goal-directed fluid therapy in elective major colorectal surgery within an Enhanced Recovery After Surgery program. *Anaesth Intensive Care.* 2014;42(6):752-760.

74. Shillcutt SK, Montzingo CR, Agrawal A, et al. Echocardiography-based hemodynamic management of left ventricular diastolic dysfunction: a feasibility and safety study. *Echocardiography.* 2014;31(10):1189-1198.

75. Benes J, Haidingerova L, Pouska J, et al. Fluid management guided by a continuous non-invasive arterial pressure device is associated with decreased postoperative morbidity after total knee and hip replacement. *BMC Anesthesiol.* 2015;15:148.

76. Colantonio L, Claroni C, Fabrizi L, et al. A randomized trial of goal directed vs. standard fluid therapy in cytoreductive surgery with hyperthermic intraperitoneal chemotherapy. *J Gastrointest Surg.* 2015;19(4):722-729.

77. Correa-Gallego C, Tan KS, Arslan-Carlon V, et al. Goal-Directed Fluid Therapy Using Stroke Volume Variation for Resuscitation after Low Central Venous Pressure-Assisted Liver Resection: A Randomized Clinical Trial. *J Am Coll Surg.* 2015;221(2):591-601.

78. Funk DJ, HayGlass KT, Koulack J, Harding G, Boyd A, Brinkman R. A randomized controlled trial on the effects of goal-directed therapy on the inflammatory response open abdominal aortic aneurysm repair. *Crit Care.* 2015;19(1):247.

79. Jammer I, Tuovila M, Ulvik A. Stroke volume variation to guide fluid therapy: is it suitable for high-risk surgical patients? A terminated randomized controlled trial. *Perioper Med (Lond).* 2015;4:6.

80. Kumar L, Kanneganti YS, Rajan S. Outcomes of implementation of enhanced goal directed therapy in high-risk patients undergoing abdominal surgery. *Indian J Anaesth.* 2015;59(4):228-233.

81. Lai CW, Starkie T, Creanor S, et al. Randomized controlled trial of stroke volume optimization during elective major abdominal surgery in patients stratified by aerobic fitness. *Br J Anaesth.* 2015;115(4):578-589.

82. Broch O, Carstens A, Gruenewald M, et al. Non-invasive hemodynamic optimization in major abdominal surgery: a feasibility study. *Minerva Anestesiol.* 2016;82(11):1158-1169.

83. Hand WR, Stoll WD, McEvoy MD, et al. Intraoperative goal-directed hemodynamic management in free tissue transfer for head and neck cancer. *Head Neck.* 2016;38 Suppl 1(Suppl 1):E1974-1980.

84. Kumar L, Rajan S, Baalachandran R. Outcomes associated with stroke volume variation versus central venous pressure guided fluid replacements during major abdominal surgery. *J Anaesthesiol Clin Pharmacol.* 2016;32(2):182-186.

85. Schmid S, Kapfer B, Heim M, et al. Algorithm-guided goal-directed haemodynamic therapy does not improve renal function after major abdominal surgery compared to good standard clinical care: a prospective randomised trial. *Crit Care.* 2016;20:50.

86. Elgendy MA, Esmat IM, Kassim DY. Outcome of intraoperative goal-directed therapy using Vigileo/FloTrac in high-risk patients scheduled for major abdominal surgeries: A prospective randomized trial. *Egyptian Journal of Anaesthesia.* 2017;33(3):263-269.

87. Gómez-Izquierdo JC, Trainito A, Mirzakandov D, et al. Goal-directed Fluid Therapy Does Not Reduce Primary Postoperative Ileus after Elective Laparoscopic Colorectal Surgery: A Randomized Controlled Trial. *Anesthesiology.* 2017;127(1):36-49.

88. Liang M, Li Y, Lin L, et al. Effect of goal-directed fluid therapy on the prognosis of elderly patients with hypertension receiving plasmakinetic energy transurethral resection of prostate. *International Journal of Clinical and Experimental Medicine.* 2017;10(1):1290-1296.

89. Luo J, Xue J, Liu J, Liu B, Liu L, Chen G. Goal-directed fluid restriction during brain surgery: a prospective randomized controlled trial. *Ann Intensive Care.* 2017;7(1):16.

90. Reisinger KW, Willigers HM, Jansen J, et al. Doppler-guided goal-directed fluid therapy does not affect intestinal cell damage but increases global gastrointestinal perfusion in colorectal surgery: a randomized controlled trial. *Colorectal Dis.* 2017;19(12):1081-1091.

91. Stens J, Hering JP, van der Hoeven CWP, et al. The added value of cardiac index and pulse pressure variation monitoring to mean arterial pressure-guided volume therapy in moderate-risk abdominal surgery (COGUIDE): a pragmatic multicentre randomised controlled trial. *Anaesthesia.* 2017;72(9):1078-1087.

92. Weinberg L, Ianno D, Churilov L, et al. Restrictive intraoperative fluid optimisation algorithm improves outcomes in patients undergoing pancreaticoduodenectomy: A prospective multicentre randomized controlled trial. *PLoS One.* 2017;12(9):e0183313.

93. Wu J, Ma Y, Wang T, Xu G, Fan L, Zhang Y. Goal-directed fluid management based on the auto-calibrated arterial pressure-derived stroke volume variation in patients undergoing supratentorial neoplasms surgery. *International Journal of Clinical and Experimental Medicine.* 2017;10(2):3106-3114.

94. Kaufmann KB, Baar W, Rexer J, et al. Evaluation of hemodynamic goal-directed therapy to reduce the incidence of bone cement implantation syndrome in patients undergoing cemented hip arthroplasty - a randomized parallel-arm trial. *BMC Anesthesiol.* 2018;18(1):63.

95. Kim HJ, Kim EJ, Lee HJ, et al. Effect of goal-directed haemodynamic therapy in free flap reconstruction for head and neck cancer. *Acta Anaesthesiol Scand.* 2018;62(7):903-914.

96. Yin K, Ding J, Wu Y, Peng M. Goal-directed fluid therapy based on noninvasive cardiac output monitor reduces postoperative complications in elderly patients after gastrointestinal surgery: A randomized controlled trial. *Pak J Med Sci.* 2018;34(6):1320-1325.

97. Zhang N, Liang M, Zhang DD, et al. Effect of goal-directed fluid therapy on early cognitive function in elderly patients with spinal stenosis: A Case-Control Study. *Int J Surg.* 2018;54(Pt A):201-205.

98. Zhao G, Peng P, Zhou Y, Li J, Jiang H, Shao J. The accuracy and effectiveness of goal directed fluid therapy in plateau-elderly gastrointestinal cancer patients: a prospective randomized controlled trial. *International Journal of Clinical and Experimental Medicine.* 2018;11(8):8516-8522.

99. Cesur S, Çardaközü T, Kuş A, Türkyılmaz N, Yavuz Ö. Comparison of conventional fluid management with PVI-based goal-directed fluid management in elective colorectal surgery. *J Clin Monit Comput.* 2019;33(2):249-257.

100. Davies SJ, Yates DR, Wilson RJT, et al. A randomised trial of non-invasive cardiac output monitoring to guide haemodynamic optimisation in high risk patients undergoing urgent surgical repair of proximal femoral fractures (ClearNOF trial NCT02382185). *Perioper Med (Lond).* 2019;8:8.

101. Godai K, Matsunaga A, Kanmura Y. The effects of hemodynamic management using the trend of the perfusion index and pulse pressure variation on tissue perfusion: a randomized pilot study. *JA Clin Rep.* 2019;5(1):72.

102. Hasanin A, Zanata T, Osman S, et al. Pulse Pressure Variation-Guided Fluid Therapy during Supratentorial Brain Tumour Excision: A Randomized Controlled Trial. *Open Access Maced J Med Sci.* 2019;7(15):2474-2479.

103. Liu F, Lv J, Zhang W, Liu Z, Dong L, Wang Y. Randomized controlled trial of regional tissue oxygenation following goal-directed fluid therapy during laparoscopic colorectal surgery. *Int J Clin Exp Pathol.* 2019;12(12):4390-4399.

104. Sujatha PP, Nileshwar A, Krishna HM, Prasad SS, Prabhu M, Kamath SU. Goal-Directed vs Traditional Approach to Intraoperative Fluid Therapy during Open Major Bowel Surgery: Is There a Difference? *Anesthesiol Res Pract.* 2019;2019:3408940.

105. Szturz P, Folwarczny P, Kula R, Neiser J, Ševčík P, Benes J. Multi-parametric functional hemodynamic optimization improves postsurgical outcome after intermediate risk open gastrointestinal surgery: a randomized controlled trial. *Minerva Anestesiol.* 2019;85(3):244-254.

106. Weinberg L, Ianno D, Churilov L, et al. Goal directed fluid therapy for major liver resection: A multicentre randomized controlled trial. *Ann Med Surg (Lond).* 2019;45:45-53.

107. Arslan-Carlon V, Tan KS, Dalbagni G, et al. Goal-directed versus Standard Fluid Therapy to Decrease Ileus after Open Radical Cystectomy: A Prospective Randomized Controlled Trial. *Anesthesiology.* 2020;133(2):293-303.

108. De Cassai A, Bond O, Marini S, et al. [Pulse pressure variation guided fluid therapy during kidney transplantation: a randomized controlled trial]. *Braz J Anesthesiol.* 2020;70(3):194-201.

109. Fischer MO, Lemoine S, Tavernier B, et al. Individualized Fluid Management Using the Pleth Variability Index: A Randomized Clinical Trial. *Anesthesiology.* 2020;133(1):31-40.

110. Iwasaki Y, Ono Y, Inokuchi R, Ishida T, Kumada Y, Shinohara K. Intraoperative fluid management in hepato-biliary-pancreatic operation using stroke volume variation monitoring: A single-center, open-label, randomized pilot study. *Medicine (Baltimore).* 2020;99(50):e23617.

111. Nicklas JY, Diener O, Leistenschneider M, et al. Personalised haemodynamic management targeting baseline cardiac index in high-risk patients undergoing major abdominal surgery: a randomised single-centre clinical trial. *Br J Anaesth.* 2020;125(2):122-132.

112. Schneck E, Schulte D, Habig L, et al. Hypotension Prediction Index based protocolized haemodynamic management reduces the incidence and duration of intraoperative hypotension in primary total hip arthroplasty: a single centre feasibility randomised blinded prospective interventional trial. *J Clin Monit Comput.* 2020;34(6):1149-1158.

113. Diaper J, Schiffer E, Barcelos GK, et al. Goal-directed hemodynamic therapy versus restrictive normovolemic therapy in major open abdominal surgery: A randomized controlled trial. *Surgery.* 2021;169(5):1164-1174.

114. Broch O, Carstens A Fau - Gruenewald M, Gruenewald M Fau - Nischelsky E, et al. Non-invasive hemodynamic optimization in major abdominal surgery: a feasibility study. (1827-1596 (Electronic)).
