# Supplementary material for: Goal-directed haemodynamic therapy during general anaesthesia for noncardiac surgery: a systematic review and meta-analysis
Source: Br J Anaesth. 2021 Dec 13;128(3):416–33. doi: 10.1016/j.bja.2021.10.046 (PMC8900265; doi:10.1016/j.bja.2021.10.046)
Supplement: Multimedia component 2 [file mmc2.pdf]

HEMODYNAMIC AND RESPIRATORY TARGETS DURING GENERAL ANESTHESIA: SYSTEMATIC REVIEW AND META-ANALYSIS

PROTOCOL

Version 2.0

August 19, 2020

TITLE

Hemodynamic and respiratory targets during general anesthesia: systematic review and meta-analysis

REGISTRATION

This version of the protocol was uploaded to figshare (figshare.com) on August 19, 2020. Version 1.0 was uploaded on June 11, 2020.

TEAM MEMBERS

Mathias Karlsson
Ida Riise Balleby
Jeppe Henriksen
Karol Dabrowski
Mark Eggertsen
Philip Caap
Maibritt Provstgaard Holm
Maria Bolther Pælestik
Caroline Boye
Lasse Vormfenne
Maria Høybye
Marie Jessen
Johanne Marie Holst
Niklas Schurmann Hansen
Andreas Magnussen
Peter Carøe Lind
Cecilie Munch Johannsen
Lara Roessler
Frederik Boe Hansen
Mikael Fink Vallentin
Mathias Holmberg
Thomas Hvid Jensen
Johannes Enevoldsen
Asger Granfeldt
Lars W. Andersen

CORRESPONDING AUTHOR

Lars W. Andersen
Research Center for Emergency Medicine
Department of Clinical Medicine
Aarhus University and Aarhus University Hospital
Email: lwandersen@clin.au.dk

AMENDMENTS

Any major modification to the protocol after registration, which may impact the conduct of the study including study eligibility criteria, information sources, search strategy, data management, selection and collection, outcomes, and data synthesis will be agreed upon by all authors listed and added as an amendment. Minor administrative corrections or clarification will require no formal documentation.

Amendments from version 1.0 to 2.0

- Changes to the search strategy
- Addition of team members
- Exclusion of trials including one-lung ventilation
- Exclusion of trials with very short duration of anesthesia
- Exclusion of trials involving cesarian section
- Exclusion of trials in interventional radiology

FINANCIAL SUPPORT

There is no funding.

CONFLICTS OF INTEREST

None of the authors have any conflicts of interest.

INTRODUCTION

Rationale

Each year, millions of patients throughout the world undergo general anesthesia for surgical procedures. During general anesthesia, the anesthetist provides medications or adjust ventilator settings to aim for certain hemodynamic (e.g. blood pressure) or respiratory (e.g. oxygen saturation) targets. Although the goal of this is to minimize complications and optimize the post-operative course, little is known about the optimal targets for a number of physiological parameters and limited guidance is provided by national and international societies. The goal of this systematic review is to identify and assess the literature on hemodynamic and respiratory targets during general anesthesia in order to provide guidance for clinicians and for future research.

Objectives (PICO question)

Population: Adults undergoing general anesthesia with invasive mechanical ventilation for non-cardiac surgery.

Interventions: A specific hemodynamic or respiratory target during general anesthesia.

Comparators: A different hemodynamic or respiratory target or no specific target during general anesthesia.

Outcomes: Clinical outcomes, including, but not necessarily limited to, intra- and post-operative complications (e.g. surgical site infections, pulmonary complication, delirium), intensive care unit admission, length of stay, and mortality.

The final outcomes will depend on the availability in the included trials.

Definitions

<u>Adults:</u> ≥ 18 years (or as defined in individual studies). Trials including both adults and children will be included if the majority of patients are adult.

<u>General anesthesia:</u> Administration of intravenous or inhalation medications to induce unconsciousness. Trials only including healthy volunteers (i.e. participants not undergoing some form of surgery) will not be included. If a trial includes both patients undergoing general anesthesia and other forms of anesthesia (i.e.

spinal anesthesia), the trial will be included if the majority of patients undergo general anesthesia. Trials involving very short duration of anesthesia (e.g. for electroconvulsive therapy) will not be included.

Invasive mechanical ventilation: Ventilation controlled or supported by a mechanical ventilator through an endotracheal tube, a tracheostomy, or a supraglottic airway.

Non-cardiac surgery: All surgery not involving the heart or the aorta. Any procedure with cardiopulmonary bypass will be considered cardiac surgery. Trials of patients receiving one-lung ventilation will also be excluded. Trials related to cesarian section or interventional radiology will be excluded.

Hemodynamic targets:

- Arterial blood pressure (diastolic, systolic, mean)
- Heart rate
- Cardiac output (including cardiac index)
- Stroke volume (including stroke volume variation and index)
- Pulse pressure or pulse pressure variation
- Combinations of various targets including goal-directed therapy

Trials comparing different fluid strategies (but without specific targets) will not be included.

Respiratory targets:

- Fraction of inspired oxygen (O_2), O_2 saturation, or partial pressure of O_2 in blood
- End-tidal carbon dioxide (CO_2) or partial pressure of CO_2 in blood
- Tidal or minute volume
- Respiratory rate
- Positive end expiratory pressure (PEEP)
- Other airway pressures (inspiratory, driving, peak)
- Recruitment strategies

Trials only testing various gas flows on the mechanical ventilator will not be included.

We recognize that individual trials might have used different definitions and whether or not individual trials are eligible for inclusion will be determined on a case-by-case basis.

Trials comparing various strategies prior to induction of general anesthesia (e.g. volume preloading or pre-oxygenation) will not be included.

METHODS

Eligibility criteria

Only randomized trials will be included. This will include quasi-randomized trials (e.g. assignment based on day of the week) as well as cluster-randomized trials. Randomized cross-over trials where the cross-over occurs within individual patients will not be included. Observational studies, case series, case reports, reviews, abstracts, editorials, comments, letters to the editor, or unpublished studies will not be included. Only English language articles will be included.

Information sources

We will search the following electronic bibliographic databases: PubMed and EMBASE. The bibliographies of included articles will be reviewed for potential additional articles. To identify ongoing trials, we will search the International Clinical Trials Registry Platform (http://www.who.int/ictrp/en/). There will be no search for unpublished studies.

Search strategy

<u>PubMed</u>

(Anesthesia, General[Mesh] OR universal anesthesia[Title/Abstract] OR universal anaesthesia[Title/Abstract] OR general anesthesia[Title/Abstract] OR general anaesthesia[Title/Abstract] OR General Surgery[Mesh] OR surgery[Title/Abstract] OR surgical[Title/Abstract] OR perioperative[Title/Abstract] OR “peri-operative”[Title/Abstract] OR intraoperative[Title/Abstract] OR “intra-operative”[Title/Abstract]) AND (((mean arterial[Title/Abstract] OR systolic[Title/Abstract] OR diastolic[Title/Abstract]) AND (pressure[Title/Abstract])) OR minute volume[Title/Abstract] OR PEEP[Title/Abstract] OR positive end expiratory pressure[Title/Abstract] OR inspiratory pressure[Title/Abstract] OR oxygen[Title/Abstract] OR CO2[Title/Abstract] OR carbon dioxide[Title/Abstract] OR hemodynamic*[Title/Abstract] OR goal directed[Title/Abstract] OR stroke volume[Title/Abstract] OR Blood Pressure[Mesh] OR Arterial Pressure[Mesh] OR Pulse[Mesh] OR Heart Rate[Mesh] OR Respiratory Rate[Mesh] OR Pulmonary Ventilation[Mesh] OR Tidal Volume[Mesh] OR Positive-Pressure Respiration[Mesh] OR Intermittent Positive-Pressure Ventilation[Mesh] OR Oximetry[Mesh] OR Cardiac Output[Mesh]) AND (randomized controlled trial[Publication Type] OR

controlled clinical trial[Publication Type] OR randomized[Title/Abstract] OR placebo[Title/Abstract] OR clinical trials as topic[Mesh:noexp] OR randomly[Title/Abstract] OR trial[Title]) NOT (animals[Mesh] NOT humans [Mesh]) AND (English[Language])

<u>EMBASE</u>
('anesthesia'/exp OR 'universal anesthesia':ti,ab OR 'universal anaesthesia':ti,ab OR 'general anesthesia':ti,ab OR 'general anaesthesia':ti,ab OR 'surgery':ti,ab OR 'surgical':ti,ab OR 'perioperative':ti,ab OR 'intraoperative':ti,ab) AND ('blood pressure'/exp OR 'arterial pressure'/exp OR 'pulse rate'/exp OR 'heart rate'/exp OR 'breathing rate'/exp OR 'lung ventilation'/exp OR 'tidal volume'/exp OR 'positive end expiratory pressure'/exp OR 'intermittent positive pressure ventilation'/exp OR 'oximetry'/exp OR 'heart output'/exp OR 'mean arterial pressure':ti,ab OR 'systolic pressure':ti,ab OR 'diastolic pressure':ti,ab OR 'minute volume':ti,ab OR 'peep':ti,ab OR 'inspiratory pressure':ti,ab OR 'oxygen':ti,ab OR 'end-tidal co2':ti,ab OR 'end-tidal carbon dioxide':ti,ab OR 'hemodynamic*':ti,ab OR 'goal directed':ti,ab OR 'stroke volume':ti,ab OR 'arterial oxygen pressure':ti,ab OR 'positive end expiratory pressure':ti,ab) AND ('randomized controlled trial'/exp OR 'controlled clinical trial'/exp OR randomly:ti,ab OR randomized:ti,ab OR placebo:ti,ab OR trial:ti,ab) NOT ('animal'/exp NOT 'human'/exp) AND ('article'/it OR 'article in press'/it) AND [english]/lim

Selection process
At least two reviewers, using pre-defined screening criteria, will independently screen all titles and abstracts retrieved from the systematic searches in duplicate. The reviewers will be blinded to authors and journal titles during this screening stage. Any disagreement regarding inclusion or exclusion will be resolved via discussion between the reviewers and with a third reviewer if needed. The Kappa-value for inter-observer variance will be calculated. In case of only minimal agreement between reviewers (i.e. a Kappa < 0.40[1]), a third reviewer will review all excluded titles and abstracts to ensure optimized sensitivity. At least two reviewers will then review, in duplicate, the full text-reports of all potentially relevant publications passing the first level of screening. Any disagreement regarding eligibility will be resolved via discussion and study authors will be contacted if pertinent. The final report will include a Preferred Reporting Items for Systematic Reviews and Meta-Analysis (PRISMA) diagram showing the number of studies remaining after each stage of the selection process. This will include reasons for exclusion of full text articles.

Data collection process

At least two reviewers using a pre-defined standardized data extraction form will extract data as pertinent to the PICO. Any discrepancy regarding the extracted data will be identified and resolved via discussion.

Data items

The following data will be extracted as relevant:

- General information
    - First author name
    - Year of publication
    - Geographical location of the study (country, continent)
    - Inclusion and exclusion criteria
    - Years of patient enrollment
    - Number of patients analyzed
    - Precise intervention and comparator
- Participants
    - Summary demographics
        - Age (mean/median)
        - Sex (proportion of females)
    - American Society of Anesthesiologist (ASA) classification
    - Type of anesthesia
    - Type of surgery
    - Length of surgery
- Relevant results

Depending on the data provided in the reviewed trials, specific data elements may be omitted or additional data elements may be extracted.

Outcomes

The final included outcomes will depend on the available data. The focus will be on clinically relevant outcomes i.e. outcomes that are relevant to both the provider and the patient. As such, trials only including surrogate outcomes or purely physiological outcomes will not be included.

Risk of bias in individual studies

At least two investigators will independently assess risk of bias for the included trials. Risk of bias will be assessed by use of the revised Cochrane risk-of-bias tool for randomized trials.[2] The assessment tool for individually-randomized parallel-group trials and the supplement for cluster-randomized parallel-group trial will be used as appropriate.

Heterogeneity, data synthesis and meta-regression

All analyses will be conducted separately for different hemodynamic and respiratory targets. Depending on the available data, subgroup analyses might be performed. These could for example be based on ASA classification or type of surgery.

Trials will be assessed for clinical (i.e. participants, interventions, and outcomes), methodological (i.e. study design or risk of bias), and potentially statistical heterogeneity.[3] If there is no substantial clinical or methodological heterogeneity, statistical heterogeneity will be assessed using forest plots, Chi-squared statistics, and I-squared statistics. A p-value of < 0.10 or I-squared statistic of >50% will indicate substantial statistical heterogeneity, and in such cases random-effects meta-analyses will be performed.[3] In the case of homogeneity, a fixed-effects model will be used. A narrative synthesis will be conducted if heterogeneity (i.e. clinical, methodological, or statistical) is deemed too substantial across studies to allow for meaningful meta-analyses. There will be no attempt to account for missing data within included trials. Number needed to treat (NNT) will be calculated based on the pooled risk ratios and various estimates of baseline risk if applicable.[4, 5]

In case of overlap in data between trials included in the meta-analyses, the risk of bias within the individual studies will be compared and the study with the lowest risk of bias will be included. If the risk of bias is similar, we will include the trial with the largest sample size.

Publication bias will be evaluated using funnel plots, the Egger test, the Begg test, and the Harbord test as appropriate, depending on the degree of heterogeneity observed.[3] However, these statistical tests will only be conducted if the number of trials is ≥ 10.[3]

In the case of substantial heterogeneity within a given meta-analysis, meta-regression will be considered to identify potential statistically significant determinants of heterogeneity in the pooled effect estimates at an alpha level of 0.05. Covariates will depend on the available data. If there is a sufficient ratio of trials to covariates, each covariate will be entered in a multivariable meta-regression model using a backward elimination approach at a p-value > 0.05. If this is not the case, only bivariable assessments will be made. Meta-regression will only be considered if the number of studies is ≥ 10 for any given comparison.[3]

If three or more interventions are compared within a single domain (e.g. three different PEEP strategies) across different trials, and the trials are otherwise homogenous (i.e. the assumption of transitivity is fulfilled), network meta-analyses will be considered. The goal of this type of analysis is to compare multiple interventions in a single analysis by combining both direct and indirect comparisons across multiple trials. In addition to estimating these direct and indirect effects, the network meta-analyses will be used to rank the interventions.

Confidence in cumulative evidence

The quality of the overall evidence will be assessed using the Grading of Recommendations Assessment, Development and Evaluation (GRADE) methodology ranging from very low quality of evidence to high quality of evidence.[6] Detailed assessment of overall risk of bias, inconsistency, indirectness, imprecision and other issues such as publication bias will be tabulated.

Data management

RevMan (The Cochrane Collaboration, 2014) will be used to perform conventional meta-analyses. SAS software, version 9.4 (SAS Institute, Cary, NC, USA) or other relevant software will be used for meta-regression and network meta-analyses if pertinent. GRADEpro (McMaster University, 2014) will be used for drafting of the GRADE tables.

Publication

Results will be reported according to appropriate PRISMA guidelines. The results will be published in a peer-reviewed journal. Depending on the results, one or more manuscripts will be published.

REFERENCES

1. McHugh, M.L., *Interrater reliability: the kappa statistic.* Biochem Med (Zagreb), 2012. **22**(3): p. 276-82.
2. Higgins, J., et al., *A revised tool for assessing risk of bias in randomized trials*, in *Cochrane Methods*, J. Chandler, et al., Editors. 2016, Issue 10 (Suppl 1): *Cochrane Database of Systematic Reviews*
3. Higgins, J. and S. Green. *Cochrane Handbook for Systematic Reviews of Interventions*. 2011. Version 5.1.0. [Updated March 2011].
4. Cates, C.J., *Simpson's paradox and calculation of number needed to treat from meta-analysis.* BMC Med Res Methodol, 2002. **2**: p. 1.
5. Altman, D.G. and J.J. Deeks, *Meta-analysis, Simpson's paradox, and the number needed to treat.* BMC Med Res Methodol, 2002. **2**: p. 3.
6. Guyatt, G.H., et al., *GRADE: an emerging consensus on rating quality of evidence and strength of recommendations.* BMJ, 2008. **336**(7650): p. 924-6.
